# Molecular Insights into Transcranial Direct Current Stimulation Effects: Metabolomics and Transcriptomics Analyses

**DOI:** 10.3390/cells13030205

**Published:** 2024-01-23

**Authors:** Bhanumita Agrawal, Soad Boulos, Soliman Khatib, Yonatan Feuermann, Julia Panov, Hanoch Kaphzan

**Affiliations:** 1 Sagol Department of Neurobiology, University of Haifa, Haifa 3103301, Israel; 2 Department of Biotechnology, Tel-Hai College, Upper Galilee 1220800, Israel; 3 Tauber Bioinformatics Research Center, University of Haifa, Haifa 3103301, Israel

**Keywords:** tDCS, neurostimulation, energy production, glycolysis, mitochondria, calcium signaling

## Abstract

Introduction: Transcranial direct current stimulation (tDCS) is an evolving non-invasive neurostimulation technique. Despite multiple studies, its underlying molecular mechanisms are still unclear. Several previous human studies of the effect of tDCS suggest that it generates metabolic effects. The induction of metabolic effects by tDCS could provide an explanation for how it generates its long-term beneficial clinical outcome. Aim: Given these hints of tDCS metabolic effects, we aimed to delineate the metabolic pathways involved in its mode of action. Methods: To accomplish this, we utilized a broad analytical approach of co-analyzing metabolomics and transcriptomic data generated from anodal tDCS in rat models. Since no metabolomic dataset was available, we performed a tDCS experiment of bilateral anodal stimulation of 200 µA for 20 min and for 5 consecutive days, followed by harvesting the brain tissue below the stimulating electrode and generating a metabolomics dataset using LC-MS/MS. The analysis of the transcriptomic dataset was based on a publicly available dataset. Results: Our analyses revealed that tDCS alters the metabolic profile of brain tissue, affecting bioenergetic-related pathways, such as glycolysis and mitochondrial functioning. In addition, we found changes in calcium-related signaling. Conclusions: We conclude that tDCS affects metabolism by modulating energy production-related processes. Given our findings concerning calcium-related signaling, we suggest that the immediate effects of tDCS on calcium dynamics drive modifications in distinct metabolic pathways. A thorough understanding of the underlying molecular mechanisms of tDCS has the potential to revolutionize its applicability, enabling the generation of personalized medicine in the field of neurostimulation and thus contributing to its optimization.

## 1. Introduction

Transcranial direct current stimulation (tDCS) is a non-invasive neuromodulation method that has gained increasing popularity in the treatment of neuropsychiatric disorders [1]. By applying a sub-threshold electrical current to specific cortical regions, tDCS modifies neuronal activity [2,3,4,5], leading to the modulation of short- and long-term neuronal plasticity [2,6,7]. However, despite the growing interest and application of tDCS in therapeutic settings, the molecular mechanisms underlying its effects are poorly understood.

One of the suggested mechanisms of action of tDCS is its effects on the metabolism of brain cells. A study performed in humans showed that anodal tDCS causes neuronal excitation, leading to an energetic depletion observed by an initial drop in the phosphocreatine (Cre/CreP) ratio compared to the sham condition [8]. This effect of tDCS on bioenergetics was also linked with ATP production and ATP homeostasis. Studies show that tDCS causes the breakdown and synthesis of ATP through mechanisms governed by mitochondria [9]. Moreover, it was suggested that the neuroprotective effect of tDCS in a mouse model of Parkinson’s disease is due to the regulation of mitochondrial dynamics [10]. Taken together, these studies indicate that tDCS modulates energy metabolism by affecting mitochondrial function and dynamics.

Since not much is known about the molecular mechanisms of tDCS, we aimed to perform broad exploratory analyses that have the potential to capture the involved metabolic and molecular processes. LC-MS/MS metabolomics and whole mRNA transcriptomics sequencing can serve as two powerful unsupervised methods for such an attempt. To the best of our knowledge, no study performed a broad metabolomics investigation of tDCS effects on brain tissue. Moreover, only four studies investigated the transcriptomic effects of tDCS. One of these studies utilized blood samples [11], and only three studies used brain tissue as the source for generating the transcriptomic data [12,13,14]. Interestingly, two of these studies emphasized that tDCS induced a differential expression of immune response-related genes, while none of them focused on metabolism-related genes.

After thorough scrutiny of these three studies, we found a single transcriptome dataset that was appropriate for our analyses as it utilized a sham control and an anodal-tDCS with no additional intervention. In addition, the tDCS protocol involved the administration of a lower current density, which better simulates the stimulations in humans.

Due to the lack of a metabolomics dataset, we generated LC-MS/MS metabolomics data in rats treated with anodal tDCS or sham. Following this, we analyzed both datasets and employed bioinformatics and machine learning approaches to investigate the impact of tDCS at metabolomic and transcriptomic levels. This comprehensive analysis enables us to explore the changes in molecular profiles associated with tDCS.

The finding of the metabolic and molecular underpinnings of tDCS is of critical importance as it would provide a better understanding of tDCS’s biological effects, which will enable its optimization and suggest means to augment tDCS efficacy. For example, pharmacological therapies that could align with the biological processes involved in tDCS could strengthen its effects. Compared to other non-invasive neurostimulation techniques, such as transcranial magnetic stimulation (TMS), tDCS stands out as a more user-friendly and cost-effective option in clinical settings [15]. Additionally, unlike TMS, which increases brain activity in the area of stimulation by triggering neurons to fire action potentials, tDCS acts by modulating ongoing activity, favoring it for excitation or inhibition, and not initiating neuronal firing, thereby reducing the risk of adverse effects and neurological complications such as seizures and other physiological challenges [16]. Moreover, despite multiple studies of tDCS in the treatment of various indications, its use is still quite limited, and it is not considered an FDA-approved method. Part of the hesitant and limited use of tDCS is derived from the ambiguity concerning its biological effects. Better knowledge of the affected biological pathways will enable the tailoring of tDCS for treating distinct disorders with known mechanisms or optimizing tDCS operational parameters for particular patients. Altogether, we believe that a full comprehension of the biological processes underlying tDCS would contribute to its dissemination in the clinical arena.

## 2. Materials and Methods

### 2.1. Animals

Six male Sprague Dawley rats were used for metabolomic experiments. All experimental protocols were performed in accordance with the guidelines of the US National Institutes of Health and approved by the Institutional Animal Care and Use Committee of the University of Haifa under the ethics number 718/20. The rats were maintained throughout the experiment in a 12 h light/dark cycle with 22 ± 2 °C ambient temperature, with food and water ad libitum. Experiments were consistently conducted during the light phase of the cycle.

### 2.2. Surgery and tDCS Treatment

**Figure 1:**
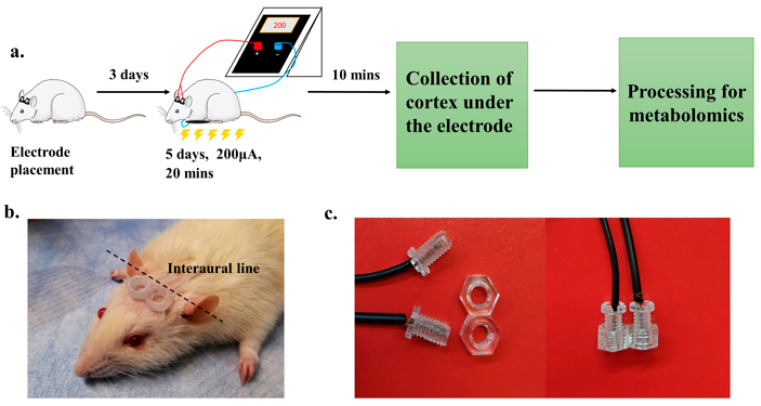
Experimental design of the tDCS for metabolomic analyses. (**a**). A total of 3 days after adhesion of electrode holders on the scalp of 2-month-old rats, tDCS (200 µA, 20 min) was administered for 5 days. Cortical tissue beneath the electrode was harvested 10 min after the last (5th) stimulation. Tissue samples were analyzed using LC-MS/MS. (**b**). Picture depicting the position of the two electrode holders along the interaural line. (**c**). A picture of the hollow electrode holders (in experiment filled with electroconductive gel) and the screws holding the stimulation wires, separate and assembled (on the right).

Before tDCS application, the rats were briefly anesthetized using isoflurane via inhalation, and their heads were shaved. Two custom-made hollow plastic electrodes (Figure 1b,c) were attached to rat heads above the parietal cortex, along the interauricular line, without any surgical procedures using cyanoacrylate-based glue (MitreBond, Unika, Newcastle, UK) only at their circumference. The two hollow electrodes were filled with electroconductive gel and were used to stimulate the rat cortices bilaterally. The rats were then allowed to recover three days before daily administration of tDCS/sham stimulations for 20 min each for 5 consecutive days (Figure 1a). The rats were awake during the stimulation procedure to avoid the effect of anesthesia on the stimulations. Then, 10 min after the last stimulation, the parietal cortices beneath the electrodes were quickly extracted on ice, immediately immersed in liquid nitrogen, and then stored at −80 °C for further processing.

### 2.3. LC-MS/MS Metabolomic Analysis

A total of 10 mg of brain tissue were homogenized and extracted with 1 mL of methanol, acetonitrile and DDW in the ratio 2:2:1. The samples were then vortexed for 5 min and sonicated with ice for 30 min. They were then frozen and thawed thereafter. The thawed samples were centrifuged at 12,000 rpm for 15 min at 4 °C. The pellet was discarded while the supernatant was filtered through 0.22 µm PTFE syringe filters (Membrane Solutions, Auburn, WA, USA) and injected into one HPLC vial for quality control (QC) and MS^2^ fragmentation.

LC-MS/MS analysis was performed with a heated electrospray ionization (HESI-II, Thermo Scientific^TM^, Waltham, MA, USA ) source connected to a Q Exactive ^TM^ Plus Hybrid Quadrupole-Orbitrap ^TM^ Mass spectrometer Thermo Scientific^TM^ (Waltham, MA, USA). ESI capillary voltage was set to 3500 V, capillary temperature was set to 300 °C, gas temperature to 350 °C, and gas flow to 10 mL/min. The mass spectra (*m*/*z* 100–1500) were acquired in positive and negative ion modes. In the mode with high resolution, FWHM = 70,000. For MS^2^ analysis, collision energy was set to 15, 50, and 100 EV.

Peak determination and peak area integration were performed with Compound Discoverer 3.3 (Thermo Xcalibur, Version 3.1.0.305). The compound’s identification was performed based on MzCloud database using MS^2^ data and the ChemSpider database using HRMS.

The table of the abundance of annotated peaks across all samples was quantile normalized and transformed to a natural logarithmic scale. Significant differences in the abundance of metabolites between groups were calculated with a two-tailed *t*-test (*p*-value < 0.05).

### 2.4. RNA-Seq Analysis

Public domain RNA-seq dataset [12] was used for gene expression analysis following tDCS stimulation. In the original study, two-month-old male Sprague Dawley rats were stimulated for 20 min, under sedated state, with different intensities of tDCS current/sham stimulation (250, 500, 2000 µA). The cerebral cortex directly under the electrode was harvested immediately after the end of the stimulation, and total RNA-seq data were generated from these tDCS-stimulated and control (sham-stimulated) samples.

For our analysis, we used only the 250 µA stimulated and sham-stimulated control samples. The decision to limit our study to that intensity was based on the assumption that this intensity is more relevant to the low-current density of tDCS applied in humans. The commonly used intensity of tDCS in humans is ~2 mA [17], which is closer to 200–250 µA stimulation than the 500–2000 µA applied in the rats, given the levels of current density. In addition, this intensity is closer to the intensity used in our metabolomics study.

Raw demultiplexed fastQ files were received from the authors of the original dataset [12]. The raw files were aligned to the rat genome (mRatBN7.2, INSDC Assembly) using a splice-aware HiSat2 algorithm [18]. Gene expression profiles were calculated using the RSEM algorithm [19]. Gene expression normalization and differential gene expression analysis were performed utilizing DeSeq2 [20]. We considered genes with *p*-value < 0.01 as significantly differentially expressed in tDCS-stimulated samples compared to sham control samples. Functional enrichment analysis of the differentially expressed genes was performed utilizing the DAVID database [21]. GSEA analysis was performed using the ‘fgsea’ R package version 3.18 [22]. Leading edge genes were extracted using the ‘fgsea’ R package. The ‘leading edge’ genes are defined as a subset of genes in the ranked list L at, or before, the point where the running sum reaches its maximum deviation from zero.

In order to determine whether a chosen pathway was overall up- or downregulated, we utilized a ‘pathway score’, similar to what was previously described [23].

$$\frac{\Sigma {log}_{2}\left(Fold\;Change\;upregulated\;genes\right)-\Sigma {log}_{2}\left(\left|Fold\;Change\;downregulated\;genes\right|\right)}{\sqrt{\Sigma {log}_{2}\left(\left|Fold\;Change\;all\;genes\right|\right)}}$$

The meaning of such a scoring system is to define whether, overall, the genes involved in a distinct pathway are upregulated or downregulated or if there is a balanced effect. The score reflects the deviation of up- or downregulation from a balanced state in SD units. Since the initial assumption is that log2FoldChange values for all genes are normally distributed, a score greater than 2 or smaller than −2 can be considered significant, meaning that the pathway is overall up- (score > 2) or downregulated (score < −2). Of note, this ‘pathway score’ does not entail a direct biological meaning concerning the final outcome of that pathway activity, as genes involved in that pathway can either promote or inhibit the pathway’s final effects. Nonetheless, the reason for this scoring is to show that tDCS effects are not random and that they do not induce an overall activation of transcription, but there is a differential effect on the various pathways.

Heat maps were generated with an online Heatmapper tool (http://heatmapper.ca/) [24], using complete linkage and Pearson distance measure to cluster the genes.

## 3. Results

In the current study, we conducted an integrative analysis of molecular mechanisms underpinning tDCS by combining a metabolomics LC-MS/MS experiment with publicly available mRNA sequencing data from rats treated with tDCS. The objective of this analysis was to uncover the molecular modifications associated with tDCS.

### 3.1. tDCS Stimulation Alters Metabolite Expression in Parietal Cortices of Rats

To examine the impact of tDCS on metabolite abundance in the parietal cortices of rats, we performed an analysis of LC-MS/MS metabolomics data obtained from rats subjected to two different conditions: 200 µA of anodal tDCS or sham stimulation (see Section 2: Materials and Methods). These stimulations were administered daily over a five-day period, and tissue samples were collected 10 min after the completion of the final stimulation (Figure 1).

**Figure 2:**
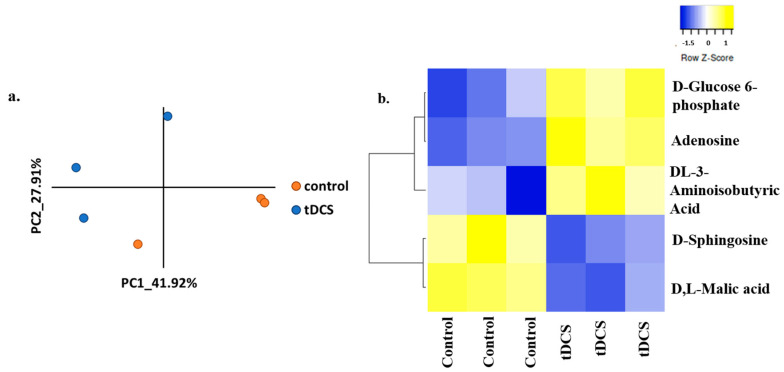
LC-MS/MS metabolomic analysis following tDCS reveals differentially expressed metabolites. (**a**). Principle component analysis of tDCS- and sham-stimulated samples based on normalized metabolite expression values. The x- and y-axis correspond to the first two principle components (PCs). Orange dots indicate control (sham) samples, while the blue dots represent the tDCS samples. (**b**). Heat map of the expression of differentially expressed metabolites following tDCS (*p*-value < 0.05). Columns represent samples taken from the sham control rat brains and the tDCS-treated rat brains, and rows represent the differentially expressed metabolites. Yellow indicates expression values larger than the average expression, while blue indicates expression values lower than the average expression.

In our analysis, we successfully quantified the expression levels of 72 high-quality metabolites (Supplementary Table S1). A principal component analysis (PCA) of the abundance matrix clearly demonstrated a differentiation between the control (sham) group of animals and the tDCS-treated group (Figure 2a). Furthermore, upon comparing the metabolite abundances in the cortices of tDCS-treated rats with control samples, we observed the differential expression of four metabolites. Specifically, three metabolites, adenosine (*p* = 0.002; FC = 3.25), Glucose 6-phosphate (G6P) (*p* = 0.009; FC = 2.77), and D,L-3-Aminoisobutyric acid (known as 3-BAIBA) (*p* = 0.048; FC = 2.75), were found to be upregulated, while two metabolites, d-sphingosine (*p* = 0.02; FC = 2.6) and D,L-Malic acid (*p* = 0.003; FC = 1.66), were downregulated in the tDCS-treated samples (Figure 2b). Of note, the mass spectrometry that we applied does not enable the separation of enantiomers.

Metabolomic data most probably reflect underlying transcriptomic changes following tDCS. Hence, the investigation of transcriptomic alterations due to tDCS can serve as an additional validation of the metabolomic findings and enhance the depth of information originally obtained from metabolomics.

### 3.2. tDCS Stimulation Alters Gene Expression in Cerebral Cortex of Rats

**Figure 3:**
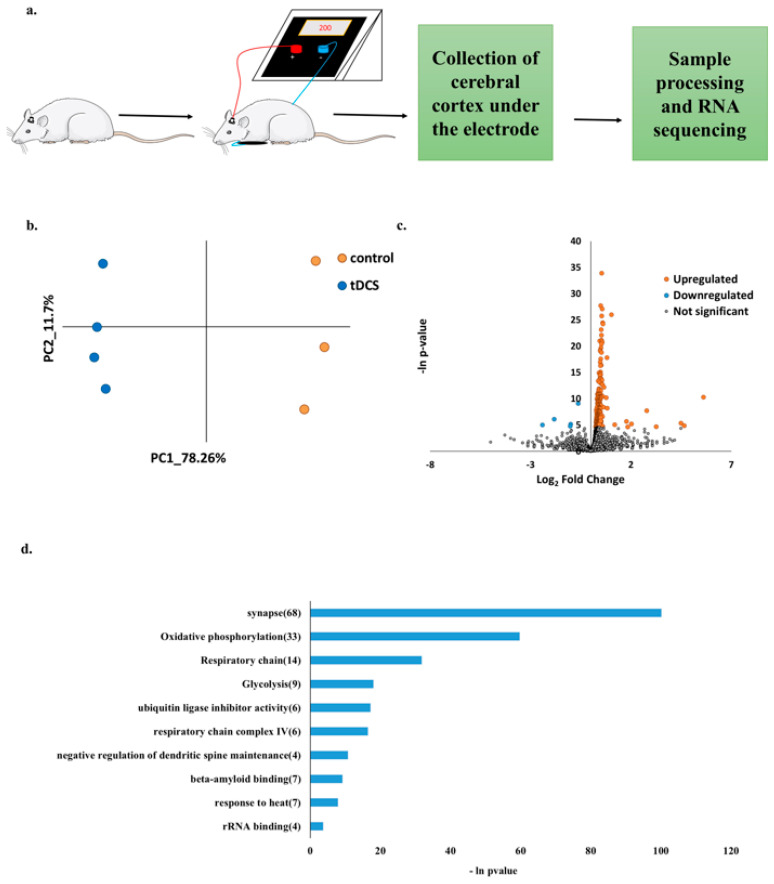
Analysis of gene expression data shows effects of tDCS on the transcriptome. (**a**). Schematic representation of experimental protocol used by Holmes et al. [12]. Rats were administered anodal tDCS (250 µA, 20 min) once. Tissue samples under the area of stimulation were collected immediately following stimulation, and total RNA was sequenced. (**b**). Principle component analysis of normalized gene expression levels in tDCS and sham control samples. The x- and y-axis represent the first two principle components (PCs). The dots colored in orange represent sham samples, and the dots colored in blue represent tDCS samples. (**c**). Volcano plot where log_2_ Fold Change (log_2_FC) (x-axis) is plotted against the –ln(*p*-values) (y-axis) for all expressed genes. Significant upregulated genes (*p*-value < 0.01; log_2_FC > 1) are highlighted in orange, and significant downregulated genes (*p*-value < 0.01; log_2_FC < −1) are highlighted in blue. (**d**). Functional pathway enrichment analysis of differentially expressed genes following tDCS showing the 10 most significantly enriched pathways (enrichment score > 2.5). The x-axis indicates the –ln(*p*-value) of the enrichment of a given pathway. In parenthesis is the number of differentially expressed genes in each pathway.

To support the metabolomics findings, we analyzed the publicly available transcriptomic dataset generated by Holmes et al. [12]. This study used an experimental setup, which was similar to our setup used for generating the metabolomics data (Figure 3a). Shortly, male rats underwent tDCS or sham treatment, and their cortices were extracted immediately after. Following, total RNA libraries were prepared and sequenced. We re-analyzed the raw sequencing data of eight samples, four sham control samples, and four samples stimulated with 250 µA intensity tDCS. Reads were aligned to the rat reference genome, and gene expressions were quantified (see Section 2: Materials and Methods).

PCA of the normalized expression profiles revealed that one of the samples was an outlier in the control group (Supplementary Figure S1). After removing the outlier, the tDCS and sham control groups were clearly separated on the PC1-PC2 plane, indicating the overall difference in transcription profiles following tDCS (Figure 3b). A differential gene expression analysis showed 251 upregulated genes and 5 downregulated genes in the tDCS-treated group compared to sham controls (Figure 3c). A pathway enrichment analysis of differentially expressed genes showed that several biological processes, including ‘synapse’, ‘oxidative phosphorylation’, ‘respiratory chain’, ‘glycolysis’, and ‘respiratory chain complex IV’, were affected by tDCS (Figure 3d).

A differential gene expression analysis focuses on each gene in isolation without considering the interconnectedness of genes within biological processes. As a result, only large statistically significant changes in gene expressions are considered in this type of examination. However, minor changes in the expression of many genes related to a single biological process can have a cumulative effect on the entire process. Thus, investigating the effect of tDCS treatment on the whole pathway should be considered. To accomplish this, we applied supervised analysis methods to study the effect of tDCS on key biological processes revealed by the unsupervised analysis.

PCA-based pathway analysis was previously applied in several transcriptomics studies [25,26] to reveal the overall effect of intervention on the whole pathway. In this analysis, only genes of a given pathway are considered as features affecting the distance between samples on the PC plane.

In addition, we utilized Gene Set Enrichment Analysis (GSEA) [27], in which genes are rank-ordered according to the fold/change ratio between the two groups of samples. If a large fraction of genes in a gene set appears near the top or the bottom of the ordered list, it will receive a high score measured by the Kolmogorov–Smirnov test. A positive score indicates an upregulated process, while a negative score indicates a downregulated process.

Given our metabolomics and transcriptomics results, we applied these supervised methods to further investigate the effect of tDCS on the ‘glycolysis’, ‘mitochondrial functioning’, ‘calcium signaling’, and ‘TCA cycle’ genes.

#### 3.2.1. tDCS Affects Glycolysis Pathway

Our finding of the significant change in levels of G6P, a key metabolite in the glycolysis pathway (Figure 2b), and differentially expressed genes associated with ‘glycolysis’ (Figure 3d) suggest that tDCS may have a broad influence on glycolysis-related genes. Thus, we conducted PCA-based pathway analysis utilizing genes from the ‘glycolysis’ pathway (see Section 2: Materials and Methods).

**Figure 4:**
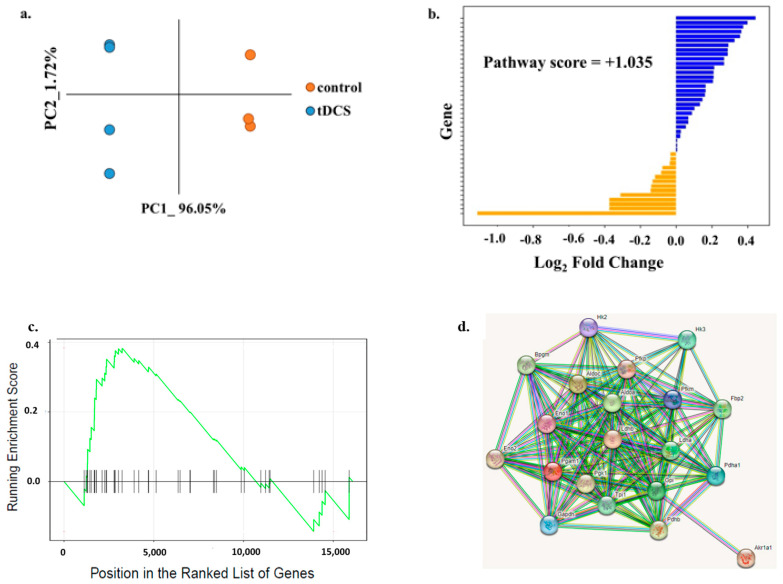
Transcriptome analysis shows tDCS effects on the glycolytic pathway. (**a**). Principle component analysis of glycolysis pathway gene expression in tDCS and sham control samples. The x- and y-axis represent the first two principle components (PCs). The dots in orange represent sham, and the dots in blue represent tDCS samples. (**b**). Bar plot indicating the log_2_ Fold Change (log_2_FC) values for expression levels of genes in ‘glycolysis’ pathway. The bars in blue represent upregulated genes (log2FC > 0), and the bars in orange represent the downregulated genes (log2FC < 0) in tDCS samples. The calculated pathway score (1.035) shows that tDCS has an overall upregulating effect on glycolytic pathway. (**c**). Gene Set Enrichment Analysis (GSEA) plot for glycolysis pathway. The y-axis represents enrichment score (ES), and the x-axis represents genes (vertical black lines) in glycolysis pathway. The green line is a running sum of the ES. (**d**). STRING protein–protein interaction network queried with ‘leading edge’ genes of the glycolysis pathway of GSEA. Colored lines between the proteins indicate various types of interaction evidence. The STRING network indicates that the ‘leading edge’ genes form a highly interconnected hub of proteins.

The tDCS-treated and control samples were clearly separated on a PC plane, indicating the overall involvement of glycolysis-related genes in tDCS (Figure 4a). In order to determine whether genes in the ‘glycolysis’ pathway were up- or downregulated following tDCS, we used the ‘pathway score’ (see Section 2: Materials and Methods). We found that the glycolysis pathway was overall upregulated following tDCS (Figure 4b, Supplementary Table S2).

Additionally, we performed GSEA on the same set of ‘glycolysis’ genes. GSEA showed that the ‘glycolysis’ pathway did not reach statistical significance (Figure 4c, Supplementary Table S3). However, using the STRING software, version 12.0 [28], we showed that the 20 ‘leading edge’ most affected genes of the pathway were inter-connected in a protein–protein interaction network (Figure 4d, Supplementary Table S3). This tight interconnectedness suggests that even slight changes in the expression levels of ‘glycolysis’ genes may lead to big changes in overall phenotype.

#### 3.2.2. tDCS Affects Mitochondrial Functioning

The metabolomic analysis revealed that tDCS affects 3-BAIBA and sphingosine, both of which are related to mitochondrial functioning [29,30,31] (Figure 2b). A gene expression analysis revealed that tDCS affects genes related to ‘oxidative phosphorylation’ and ‘respiratory chain reaction’, which are key processes taking place in the mitochondria [15,18] (Figure 3d). Thus, we next investigated genes associated with ‘mitochondrial functioning’ utilizing the MitoCarta database [32,33].

**Figure 5:**
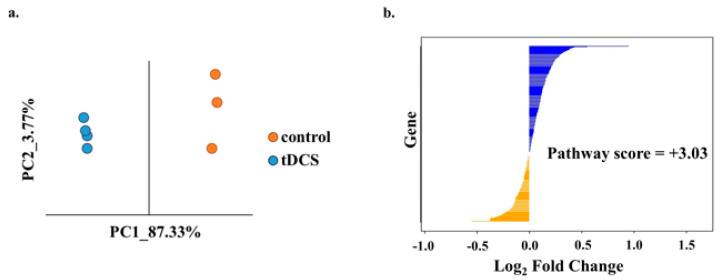
Transcriptome analysis shows tDCS effects on the mitochondria-related genes. (**a**). Principle component analysis of the expression of mitochondria-related genes (MitoCarta) in tDCS and sham control samples. The x- and y-axes represent the first two principle compinents (PCs). The dots in orange represent sham and the dots in blue represent tDCS samples. (**b**) Bar plot indicating Fold Change (log2FC) values for expression levels of mitochondria-related genes. The bars in blue represent upregulated genes (log2FC > 0), and the bars in orange represent the downregulated genes (log2FC < 0) in tDCS samples. The calculated pathway score (3.03) shows that tDCS has an overall upregulating effect on mitochondrial genes.

The PCA-based pathway analysis using 788 genes from the MitoCarta found in our data revealed a clear separation of samples on the PC plane (Figure 5a), indicating that overall mitochondria-related genes are affected by tDCS stimulation. Utilizing the overall ‘pathway score’, we found that the mitochondrial genes were upregulated following tDCS treatment (Figure 5b, Supplementary Table S4).

**Figure 6:**
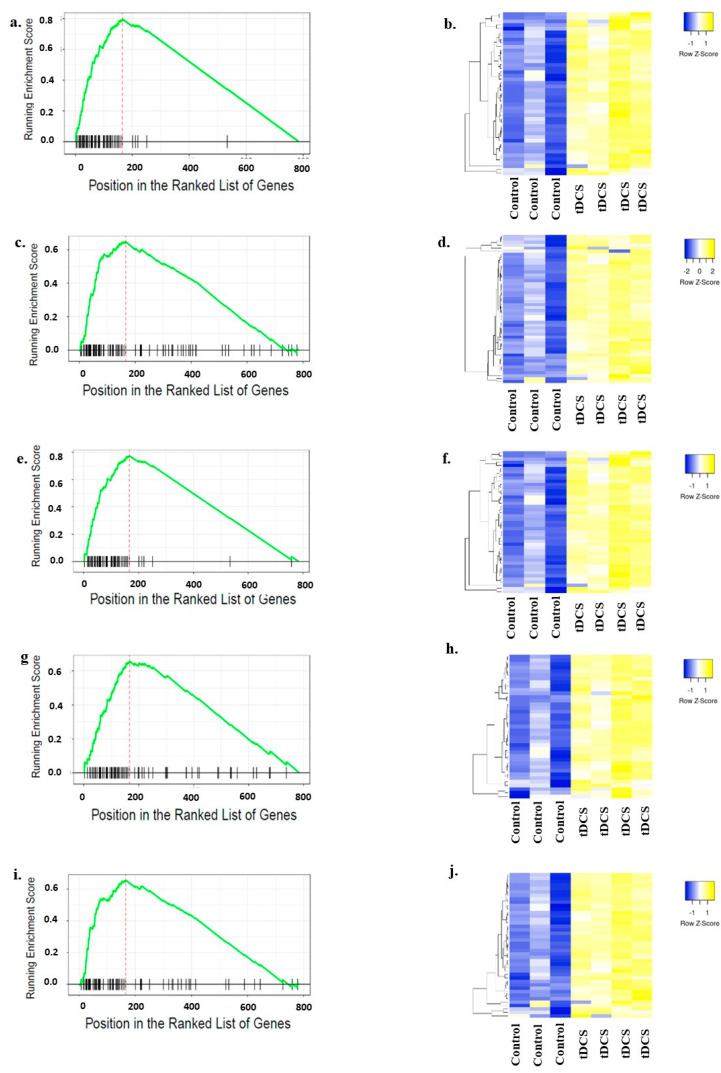
Gene Set Enrichment Analysis (GSEA) of mitochondria-related Gene Ontology (GO) terms. GSEA plots for five most significantly enriched mitochondria-related GO terms. The y-axis represents enrichment score (ES), and the x-axis represents genes (vertical black lines) in each GO term. The green line is a running sum of the ES. The expression levels of the ‘leading edge’ genes for each of the GO terms were used to generate heat maps. Columns represent samples, and rows represent genes. Yellow indicates expression values larger than the average expression, while blue indicates expression values lower than the average expression. (**a**). GSEA plot for ‘mitochondrial respirasome’ GO term. (**b**). Heat map representing expression levels of ‘leading edge’ genes in the ‘mitochondrial respirasome’ GO term. (**c**). GSEA plot for ‘ATP metabolic process’ GO term. (**d**). Heat map representing expression levels of ‘leading edge’ genes in the ‘ATP metabolic process’ GO term. (**e**). GSEA plot for ‘respiratory chain complex’ GO term. (**f**). Heat map representing expression levels of ‘leading edge’ genes in the ‘respiratory chain complex’ GO term. (**g**). GSEA plot for ‘oxidoreductive complex’ GO term. (**h**). Heat map representing expression levels of ‘leading edge’ genes in the ‘oxidoreductive complex’ GO term. (**i**). GSEA plot for ‘oxidative phosphorylation’ GO term. (**j**). Heat map representing expression levels of ‘leading edge’ genes in the ‘oxidative phosphorylation’ GO term. The name of the genes used for generating these heatmaps are shown in Supplementary Table S3.

The large number of genes from the MitoCarta database might hinder a clear understanding of the molecular pathways that play a significant role following tDCS. Thus, for the GSEA, we separated these 788 genes into known pathways using Gene Ontology (GO) terms. Performing GSEA on each GO term individually, we found that ‘mitochondrial respirasome’, ‘ATP metabolic process’, ‘respiratory chain complex’, ‘oxidoreductase complex’, and ‘oxidative phosphorylation’ were the most significant GO terms (Figure 6; Supplementary Table S3). Interestingly, all mitochondria-related pathways were upregulated.

**Figure 7:**
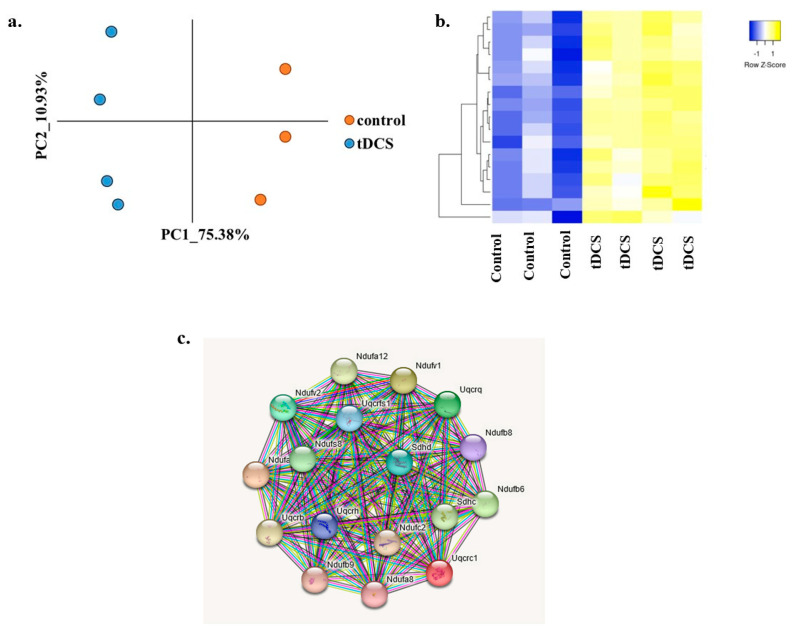
Mitochondria-related ‘leading edge’ genes are highly interconnected. (**a**). Principle component analysis of expression of 17 ‘leading edge’ genes from the five most significant mitochondria-related GO terms. The x- and y-axis represent the first two principle components (PCs). The dots in orange represent sham, and the dots in blue represent tDCS samples. (**b**). Heat map of expression of ‘leading edge’ genes from the mitochondria-related GO terms. Columns represent samples, and rows represent genes. Yellow indicates expression values larger than average expression, while blue indicates expression values lower than average expression. (**c**). The STRING protein–protein interaction network queried with 17 ‘leading edge’ genes from mitochondria-related GO terms, indicating that these genes are highly interconnected. Colored lines between the proteins indicate the various types of interaction evidence. The name of the genes used for generating these heatmaps are shown in Supplementary Table S3.

We combined all genes identified as ‘leading edge’ genes in each of the analyzed GO terms and found 17 genes that were common in all of the evaluated GO terms (Figure 7a, Supplementary Table S5). To validate that these 17 genes are affected by tDCS, we performed PCA using only these 17 shared genes and observed a clear separation between the control and tDCS-treated samples (Figure 7b), further indicating the importance of these 17 shared genes in the molecular mechanisms of tDCS. In addition, the protein–protein STRING interaction analysis revealed that these 17 genes are highly interconnected and can be considered hub genes of mitochondrial functioning that are affected by tDCS (Figure 7c).

#### 3.2.3. TCA Cycle Is Affected by tDCS

Our metabolomics and transcriptomics results indicate that both mitochondrial functioning and glycolysis are affected by tDCS. The key pathway that connects these two processes is the tricarboxylic acid (TCA) cycle. In addition, the metabolite malic acid that was significantly reduced by tDCS (Figure 2, Supplementary Table S1) is part of the TCA cycle. The TCA cycle is a crucial part of energy production and cellular respiration. It also serves as an important connecting link between glycolysis, which takes place in the cytosol, and oxidative phosphorylation, which takes place in the mitochondria [34]. In neurons, TCA plays an important role in the production of neurotransmitters [35] and in calcium signaling [36].

**Figure 8:**
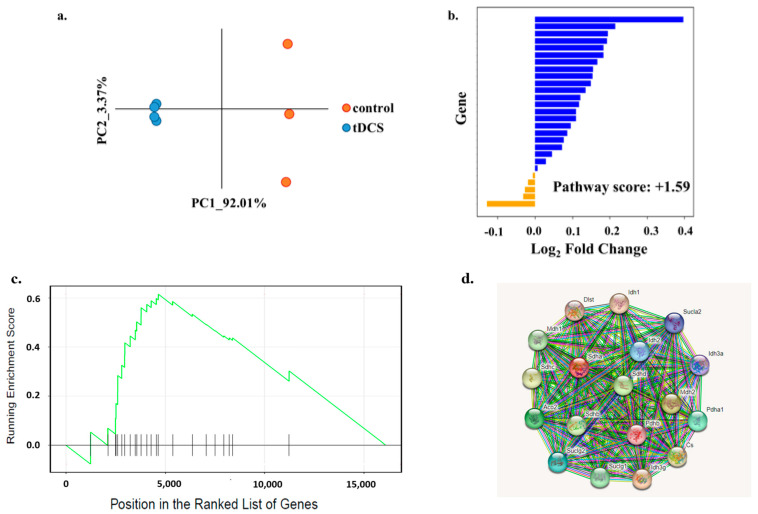
tDCS affects ‘TCA cycle’ pathway. (**a**). Principle component analysis of expression of genes from the ‘TCA cycle’ pathway in tDCS and sham control samples. The x- and y-axis represent the first two principle components (PCs). The dots in orange represent sham-stimulated samples, and the dots in blue represent tDCS samples. (**b**). Bar plot indicating the log2 Fold Change (log2FC) values for expression levels of ‘TCA cycle’ genes. The bars in blue represent upregulated genes (log2FC > 0), and the bars in orange represent the downregulated genes (log2FC < 0) in tDCS samples. The calculated pathway score (1.59) shows that tDCS has an overall upregulating effect on the ‘TCA cycle’ pathway. (**c**). Gene Set Enrichment Analysis (GSEA) plot for ‘TCA cycle’ pathway. The y-axis represents enrichment score (ES), and the x-axis represents genes (vertical black lines) in ‘TCA cycle’ pathway. The green line is a running sum of the ES. (**d**). The STRING protein–protein interaction network queried with ‘leading edge’ genes of the ‘TCA cycle’ pathway of GSEA indicates that all 18 ‘leading edge’ genes form a highly interconnected hub of proteins. Colored lines between the proteins indicate the various types of interaction evidence.

We found that 27 genes from the TCA cycle pathway were expressed in our dataset. A PCA-based pathway analysis showed a clear separation of the tDCS-treated and sham groups, indicating an effect of tDCS on the TCA cycle genes (Figure 8a). Utilizing the overall ‘pathway score’, we further observed that these genes were overall upregulated following tDCS (Figure 8b, Supplementary Table S6).

GSEA showed that the ‘TCA cycle’ pathway was significantly affected (Figure 8c) and that the 18 ‘leading edge’ genes were highly interconnected in the protein–protein interaction network (Figure 8d, Supplementary Table S3).

#### 3.2.4. tDCS Affects Calcium Signaling

Calcium dynamics is a major signaling pathway that directly modulates mitochondrial functioning via the regulation of mitochondrial respiration enzymatic activity and ATP synthesis [37,38]. Additionally, as aforementioned, in the functional enrichment analysis of the differentially expressed genes, ‘synapse’ came out as the most significantly enriched GO term in tDCS-treated samples (Figure 3d), and it is well established that synaptic transmission is calcium-dependent. Moreover, in the metabolomic analysis, we found upregulated levels of adenosine and downregulated levels of sphingosine, both involved in cellular calcium signaling [39,40]. Altogether, these facts indicate that calcium signaling is involved in tDCS. Hence, we further investigated the effect of tDCS on calcium-related genes utilizing the CaGeDB database, which is a curated collection of all calcium-related genes [41].

**Figure 9:**
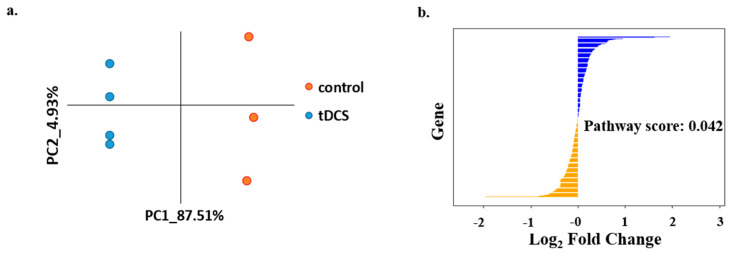
tDCS affects expression of calcium-related genes. (**a**). Principle component analysis of expression of calcium-related genes from the CaGeDB database. The x- and y-axis represent the first two principle components (PCs). The dots in orange represent sham, and the dots in blue represent tDCS samples. (**b**). Bar plot indicating the log2 Fold Change (log2FC) values for expression levels of CaGeDB calcium-related genes. The bars in blue represent upregulated genes (log2FC > 0), and the bars in orange represent the downregulated genes (log2FC < 0) in tDCS samples. The calculated pathway score (0.042) shows that tDCS has an overall upregulating effect on calcium-related genes.

**Figure 10:**
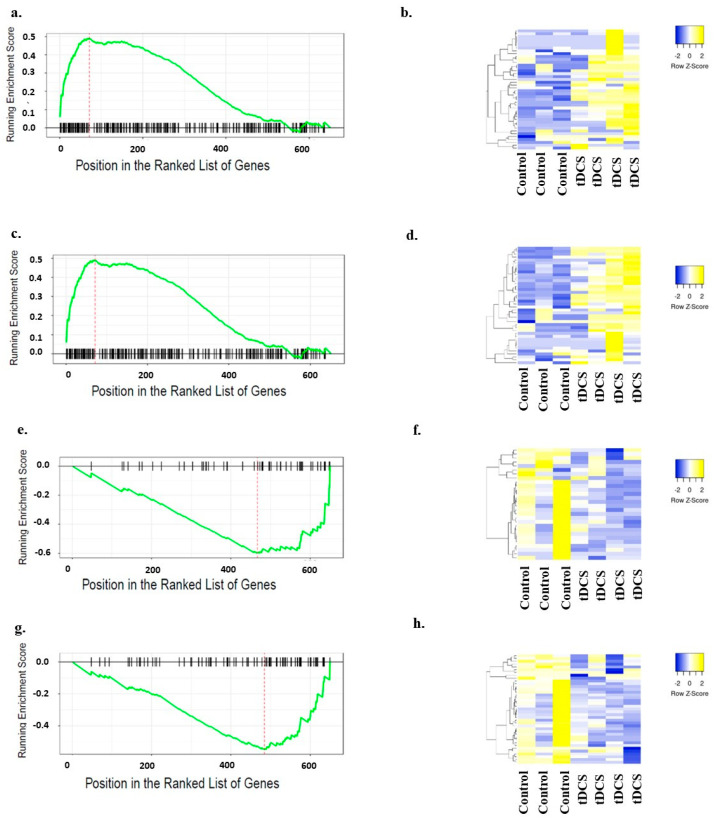
Gene Set Enrichment Analysis (GSEA) of calcium-related significantly enriched Gene Ontology (GO) terms. GSEA plots for four most significantly enriched calcium-related GO terms. Two of the calcium-related GO terms were upregulated, and the other two were downregulated. The y-axis represents enrichment score (ES), and the x-axis represents genes (vertical black lines) in each GO term. The green line is a running sum of the ES. The expression levels of the ‘leading edge’ genes for each of the GO terms were used to generate heat maps. Columns represent samples, and rows represent genes. Yellow indicates expression values larger than the average expression, while blue indicates expression values lower than the average expression. (**a**). GSEA plot for ‘external stimuli’ GO term. (**b**). Heat map representing expression levels of ‘leading edge’ genes in the ‘external stimuli’ GO term. (**c**). GSEA plot for ‘immune system process’ GO term. (**d**). Heat map representing expression levels of ‘leading edge’ genes in the ‘immune system process’ GO term. (**e**). GSEA plot for ‘neurotransmitter secretion’ GO term. (**f**). Heat map representing expression levels of ‘leading edge’ genes in the ‘neurotransmitter secretion’ GO term. (**g**). GSEA plot for ‘synaptic membrane’ GO term. (**h**). Heat map representing expression levels of ‘leading edge’ genes in the ‘synaptic membrane’ GO term. The name of the genes used for generating these heatmaps are shown in Supplementary Table S3.

A PCA using CaGeDB genes (Supplementary Table S7) showed a clear separation between tDCS and control samples (Figure 9a). However, THE overall ‘pathway score’ did not show a clear activation or inhibition of the calcium signaling (Figure 9b). Genes in the CaGeDB database belong to multiple molecular pathways and cellular processes; thus, to evaluate which of these molecular pathways or cellular processes were mostly affected by tDCS, we separated the CaGeDB database into known molecular processes using GO terms. Next, we performed GSEA on each of the GO terms separately. The GO terms ‘response to external stimuli’ and ‘immune system process’ were the most significantly upregulated following tDCS (Figure 10a–d, Supplementary Table S3), while the ‘neurotransmitter secretion’ and ‘synaptic membrane’ pathways were the most significantly downregulated (Figure 10e–h, Supplementary Table S3).

**Figure 11:**
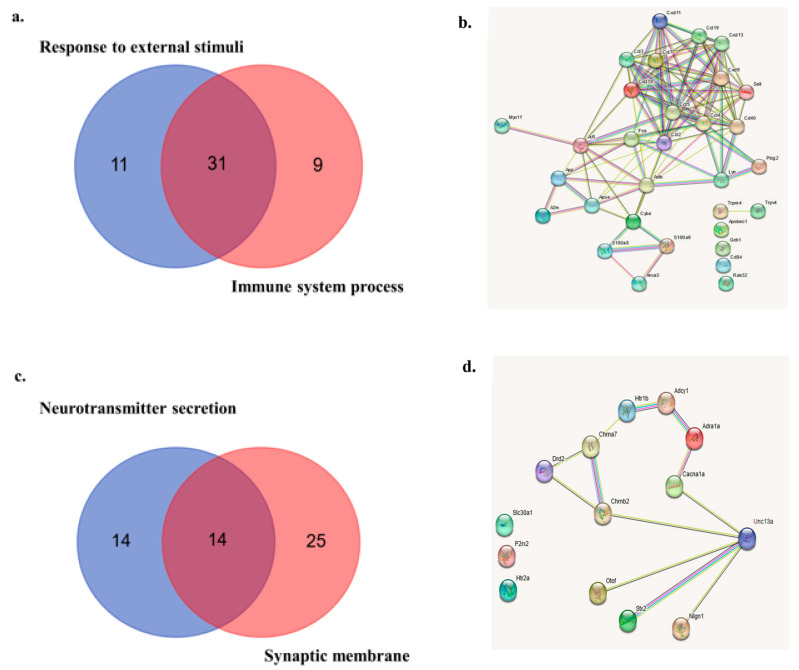
Protein–protein interaction hubs of ‘leading edge’ genes from the up- and down-regulated calcium-related GO terms. (**a**). Venn diagram showing ‘leading edge’ genes from two upregulated calcium-related GO terms: ‘external stimuli’ and ‘immune system process’. A total of 31 genes were common between the ‘leading edge’ genes of these two GO terms. (**b**) The STRING protein-protein network queried with the 31 common ‘leading edge’ genes of the upregulated calcium-related GO terms of GSEA. Colored lines between the proteins indicate the various types of interaction evidence. The STRING network indicates that 25 of the 31 common ‘leading edge’ genes form a highly interconnected hub of proteins. (**c**). Venn diagram showing ‘leading edge’ genes from two downregulated calcium-related GO terms: ‘neurotransmitter secretion’ and ‘synaptic membrane’. A total of 14 genes were common in these two GO terms. (**d**). The STRING protein–protein interaction network queried with 14 common ‘leading edge’ genes of the upregulated calcium-related GO terms of GSEA. Colored lines between the proteins indicate the various types of interaction evidence. The STRING network indicates that 11 of the 14 common ‘leading edge’ genes form an interconnected hub of proteins.

The calcium-related pathways, ‘response to external stimuli’ and ‘immune system process’, had 31 overlapping ‘leading edge’ genes (Figure 11a, Supplementary Table S8). Of these 31 overlapping genes, 25 were highly interconnected based on the protein–protein interaction analysis (Figure 11b). The downregulated calcium-related pathways, ‘neurotransmitter secretion’ and ‘synaptic membrane’, had 14 overlapping ‘leading-edge’ genes (Figure 11c, Supplementary Table S9). The protein–protein interaction network revealed that 11 of these genes were interconnected (Figure 11d).

## 4. Discussion

In this study, we investigated the impact of tDCS on metabolic pathways. By analyzing independent metabolomics and transcriptomics datasets using bioinformatics and machine learning tools, we aimed to identify the specific biological pathways influenced by tDCS and to gain a better understanding of its molecular effects.

The unsupervised analysis of metabolomics data using PCA showed a clear separation between tDCS and sham control groups (Figure 2a). In addition, five metabolites were differentially expressed following tDCS (Figure 2b), three were upregulated (glucose, adenosine, and DL-3-Aminoisobutyric acid), and two were downregulated (D-Sphingosine and DL-Malic acid).

Glucose 6-phosphate (G6P) plays a crucial role in the glycolytic pathway, which is also a determinant of cellular energy levels. Once glucose enters the cell, it is phosphorylated by hexokinase to become G6P, which prevents its diffusion out of the cell membrane back to the blood. G6P acts as the main control point for glucose homeostasis in cells [42,43], and elevated levels of G6P in tDCS-treated samples indicate changes in the energy demand of brain cells, which was also previously shown in humans [9,44]. Adenosine is a purine nucleotide commonly found in its phosphorylated form of ATP/ADP in the body. In the brain, adenosine mainly acts as an inhibitory neuromodulator [45] and controls many physiological processes, such as learning and memory, neuronal plasticity, and astrocytic activity [46]. It was shown that physiological manipulations such as increased energy demand [47], NMDA receptor activation, and intracellular acidification [48] increase the levels of extracellular adenosine. Hence, an increase in adenosine levels following tDCS indicates changes in cellular energy or neuronal activity levels, especially given that extracellular adenosine levels are considered a sensitive indicator of cellular energy demands [49]. Moreover, 3-BAIBA, the third upregulated metabolite, is formed as a result of thymine catabolism [50], and its levels increase as a result of enhanced mitochondrial activity [51]. Previous studies showed that 3-BAIBA reduces reactive oxygen species (ROS), which are mostly produced by mitochondria [52,53]. In addition, 3-BAIBA was shown to reduce ROS levels and decrease apoptosis in neuronal model cells of the PC12 cell line [54]. The fact that these three metabolites are upregulated by tDCS suggests that tDCS modulates mitochondrial functioning and cellular energy production processes.

The metabolites sphingosine and malic acid were downregulated following tDCS. Sphingosine is a primary metabolite that forms the base of most sphingolipids, which are essential components of the neuronal membrane. Sphingosine is produced by the catabolism of ceramides, and the free sphingosine is then recycled for the production of ceramides in the ER or phosphorylated to produce sphingosine-1 phosphate (S1P) [55]. The levels of these three molecules, ceramide, sphingosine, and sphingosine-1-phosphate, are constantly balanced to control the membrane dynamics and physiological functioning of neural cells [56]. The two critical enzymes—ceramide synthase [57] and sphingosine kinase-2 [58]—that are related to sphingosine metabolism are present in the mitochondria. A further accumulation of ceramide in mitochondria leads to increased ROS levels [31], decreased ATP production [59], and the induction of apoptosis [60]. S1P, on the other hand, is responsible for maintaining oxidative stress [61], mitochondrial respiration [58], ATP production [62], and also, to some extent, the mobilization of calcium in the mitochondria [30]. This role of sphingosine in calcium homeostasis is not limited to mitochondria and also plays an important role in cellular calcium signaling in general [39,40]. Malic acid, in its ionized form, malate, is a metabolite in the TCA cycle. Malate serves as a key metabolite in mitochondrial processes, such as the malate–citrate and the malate–aspartate shuttles, and as such, the interaction between malate levels and mitochondrial functioning is bidirectional [63,64,65]. Taken together, the reduction of sphingosine and malic acid levels following tDCS could again indicate that tDCS modulates mitochondrial functioning, bioenergetics, and calcium signaling.

It is probable that fast metabolic alterations following tDCS lead to subsequent transcriptomic changes. Hence, we next investigated publicly available transcriptomic data, which was generated following an experimental design similar to the experimental design we used in the metabolomics study (Figure 3a).

The principal component analysis of the gene expression data revealed a clear separation between sham and tDCS samples on the PC1-PC2 plane (Figure 3b), indicating overall differences between the sham and tDCS samples. A pathway enrichment analysis of differentially expressed genes revealed several biological processes affected by tDCS, including ‘synapse’, ‘oxidative phosphorylation’, ‘respiratory chain’, ‘glycolysis’, and ‘respiratory chain complex IV’ (Figure 3d). Interestingly, three of the five pathways, ‘oxidative phosphorylation’, ’respiratory chain’, and ‘respiratory chain complex IV’, are directly related to mitochondria.

‘Oxidative phosphorylation’ by the respiratory chain complex is a process that takes place in the mitochondrial inner membrane, which determines the production of ROS in the cells [66,67,68,69]. Increased ROS levels are associated with cell death and neurodegenerative disorders [70,71,72]. Hence, affecting ROS levels using tDCS might be beneficial for treating these diseases. This approach of modulating ROS levels by tDCS is indicated in our results that show an increase in the expression of 3-BAIBA, which was shown to act as a quencher for ROS [54]. Another piece of supporting evidence from our results on the ability of tDCS to affect oxidative stress is suggested by the decrease in D-sphingosine, a molecule that plays a role in maintaining oxidative stress and mitochondrial respiration and regulating ATP production [73]. Coinciding with the effects of tDCS on mitochondria, which modulate ATP production, our observed effects of tDCS on glycolytic pathways also determine energy metabolism, especially in the brain [8,74].

The combination of both metabolomic and transcriptomic analyses indicated significant changes in G6P metabolite levels (Figure 2b) and in genes of the ‘glycolysis’ pathway following tDCS (Figure 3d). GSEA identified 20 genes from the glycolysis pathway that were all upregulated (Figure 4c,d). Although, due to a small size effect, none of these genes was significant by itself; together, they encode for 9 out of the 10 enzymatic steps of glycolysis, from glucose to lactate (Supplementary Figure S2). We posit that being a cascade process, a slight increase in each of these steps would generate an avalanche effect, leading to a significant final outcome from this pathway. These changes in glucose metabolism highlight the effects of tDCS on energy production processes, similar to previous reports [8,9,74,75,76].

Mitochondrial functioning is tightly associated with glycolysis activity [77]. The metabolomic analysis revealed that tDCS affects 3-BAIBA and D-sphingosine, both related to mitochondrial functioning (Figure 2b). Also, transcriptome analyses found that tDCS affects mitochondrial-related genes (Figure 3d and Figure 5). We identified 17 upregulated and highly interconnected mitochondrial genes (Figure 7). These 17 genes encode for most of the enzymes that constitute the electron transport chain complexes, mainly complex-I. Again, being a cascadic process, the final output of a series of small increments might be large.

TCA is the pathway that connects glycolysis and mitochondrial functioning. In the metabolomic analyses, we found that malic acid is significantly downregulated. The ionized form of malic acid, malate, is an intermediate of the TCA cycle. As expected, the transcriptomic analysis also showed that TCA was strongly affected by tDCS (Figure 8). GSEA analysis showed that the TCA pathway was significantly upregulated, and 18 genes were identified as the ‘leading edge’ (Figure 8c,d). TCA cycle provides intermediate metabolites that serve for the production of necessary molecules beyond the ones required for ATP production, such as amino acids, lipids, and nucleotides [78]. Hence, tDCS possibly indirectly also affects additional pathways beyond mitochondria and energy production.

Given multiple hints that calcium signaling is involved in energy production processes that are affected by tDCS and given our previous study showing that tDCS modulates calcium dynamics [79,80], we investigated whether calcium-related genes were also altered by tDCS. We found that, as a whole, calcium-related genes are significantly affected by tDCS (Figure 9a). Yet, these genes relate to multiple diverse pathways with variable effects of tDCS on these pathways. Within the calcium-related genes, the genes involved in the ‘immune response’ were upregulated (Figure 10a–d). This result corresponds with the findings of Holmes et al. [12]. It is possible that even a low-current intensity of 250 μA (current density of ~10 μA/mm^2^ for 20 min) causes some tissue damage and an immediate inflammatory response. Interestingly, the downregulated calcium-related genes (Figure 10e–h) showed that one of the highly connected hub genes was Unc13a (Figure 11d). Unc13a is an important protein in presynaptic functioning, which plays a role in vesicle maturation and in priming presynaptic vesicles prior to their fusion [81]. Another two hub genes encode for nicotinic cholinergic receptors subunits Chrnb2 and Chrna7 (Figure 11d), which are calcium channels [82,83]. It is possible that this is a homeostatic response aimed at reducing calcium dynamics following tDCS. This resonates, to some extent, with a study that showed that nicotine administration abolished tDCS-induced plasticity and is reversed by a calcium channel inhibitor [84].

## 5. Conclusions

To conclude, we utilized metabolome and transcriptome datasets to study tDCS effects and found an intriguing interaction between these two levels. We observed that tDCS had multiple effects on metabolism, mostly related to mitochondria and energy production processes such as glycolysis and the TCA cycle. There is no current understanding of how an electrical current induces such metabolic effects. However, we also observed the effects of tDCS on calcium-related pathways, which coincides with previous studies by us and others [79,80,85,86,87]. Calcium is known to modulate mitochondrial functioning [88,89,90], affecting ATP production [90] and regulating oxidative stress [91,92]. Of note, calcium–mitochondria interaction is bidirectional. As much as calcium affects mitochondrial functioning, mitochondria affect calcium dynamics by acting as a buffering system of cytosolic calcium, modulating its intracellular levels [38,89]. Calcium can have additional effects on energy production beyond its effects on the mitochondria, as it regulates the breakdown process of glycogen into glucose-6-phosphate [93], which is the first step of glycolysis and was found to be upregulated in our metabolomic analyses. Moreover, calcium was found to couple neuronal activity to ATP synthesis by activating the malate-aspartate shuttle to regulate the NAD^+^/NADH ratio, which, in turn, upregulates glycolysis and pyruvate production and enhances mitochondrial respiration [65]. Taken together, it is plausible that calcium is the missing link between tDCS and the observed metabolic effects. Moreover, it is likely that these calcium-initiated metabolic processes are later converted to transcriptomic changes as the transcription machinery is ignited. Hence, one caveat of the herein study is that the publicly available transcriptomic data were generated from brain samples taken almost immediately following the tDCS session [12]. We assume that this brief time frame was not sufficient for full transcriptional processes to take place. It is probable that if tissue sampling was performed at several time points following tDCS, stronger effects could have been observed, indicating that future time-lapse studies are crucial for a better comprehension of tDCS effects and their sequence. Moreover, omics serve as a robust platform to explore the multidisciplinary effects of a neuromodulatory technique, like tDCS. Given the dynamic impact of tDCS on neural networks and molecular frameworks, a pragmatic and systems biology approach [94,95] is essential to carry out comprehensive research in this area.

## Data Availability

Data are contained within the article and provided in supplementary information.

## Supplementary Materials

The following supporting information can be downloaded at: https://www.mdpi.com/article/10.3390/cells13030205/s1, Figure S1: Principle component analysis (PCA) of tDCS- and sham-stimulated samples based on normalized gene expression values; Figure S2: Schematic representation of glycolysis pathway; Table S1: Cortex_metabolomics; Table S2: Glycolysis; Table S3: GSEA; Table S4: Mitochondria; Table S5: 17 common mitochondria-related ‘leading edge’ genes; Table S6: TCA cycle; Table S7: Calcium; Table S8: 31 upregulated ‘leading edge’ calcium-related genes; Table S9: 14 downregulated ‘leading edge’ calcium-related genes.
