# Supplementary material for: Molecular Insights into Transcranial Direct Current Stimulation Effects: Metabolomics and Transcriptomics Analyses
Source: Cells. 2024 Jan 23;13(3):205. doi: 10.3390/cells13030205 (PMC10854682; doi:10.3390/cells13030205)
Supplement: Supplementary file 1 [file cells-13-00205-s001.zip › Supplementary Figures and Legends.pdf]

Supplementary Figures

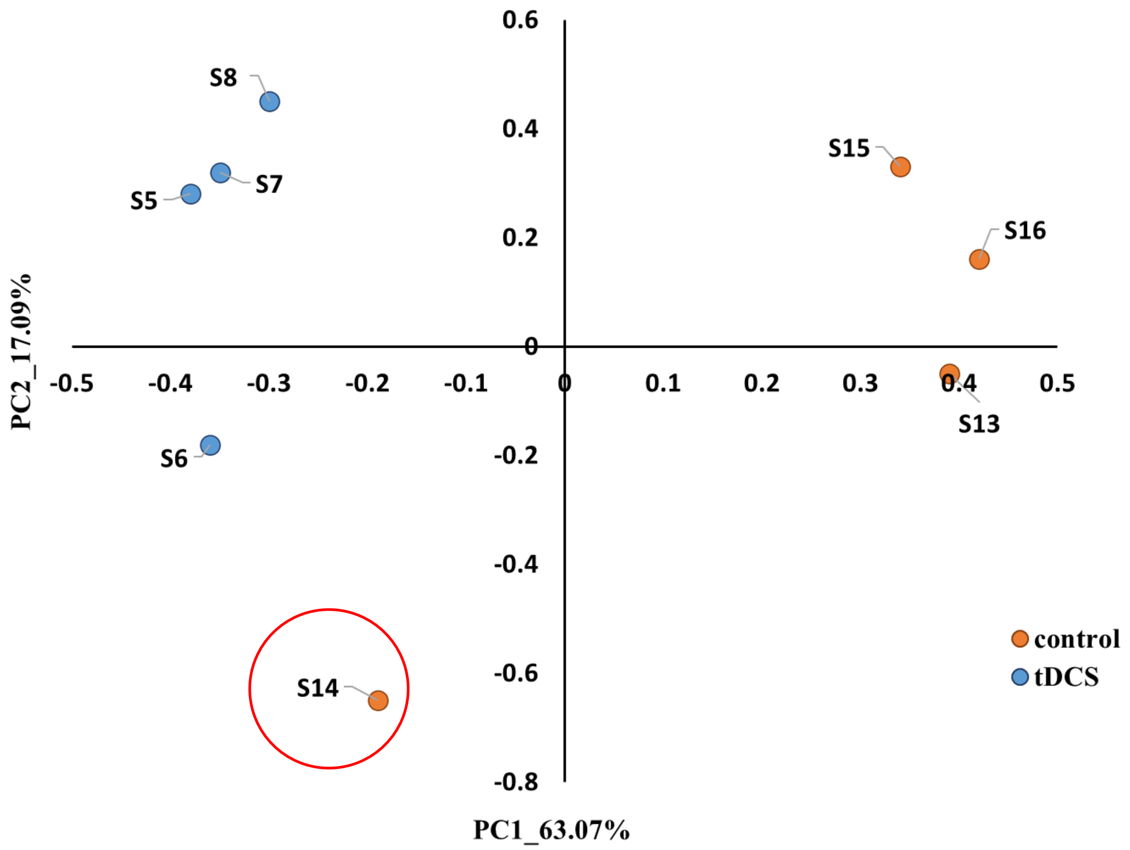

Supplementary Figure S1: Principal component analysis (PCA) of tDCS and sham stimulated samples based on normalized gene expression values. The x- and y-axes represent the first two principal components (PCs). The dots in orange represent sham and the dots in blue represent tDCS samples. PCA revealed that one sample among the sham group is an outlier (circled in red). All other samples are clustered together in two biologically relevant groups.

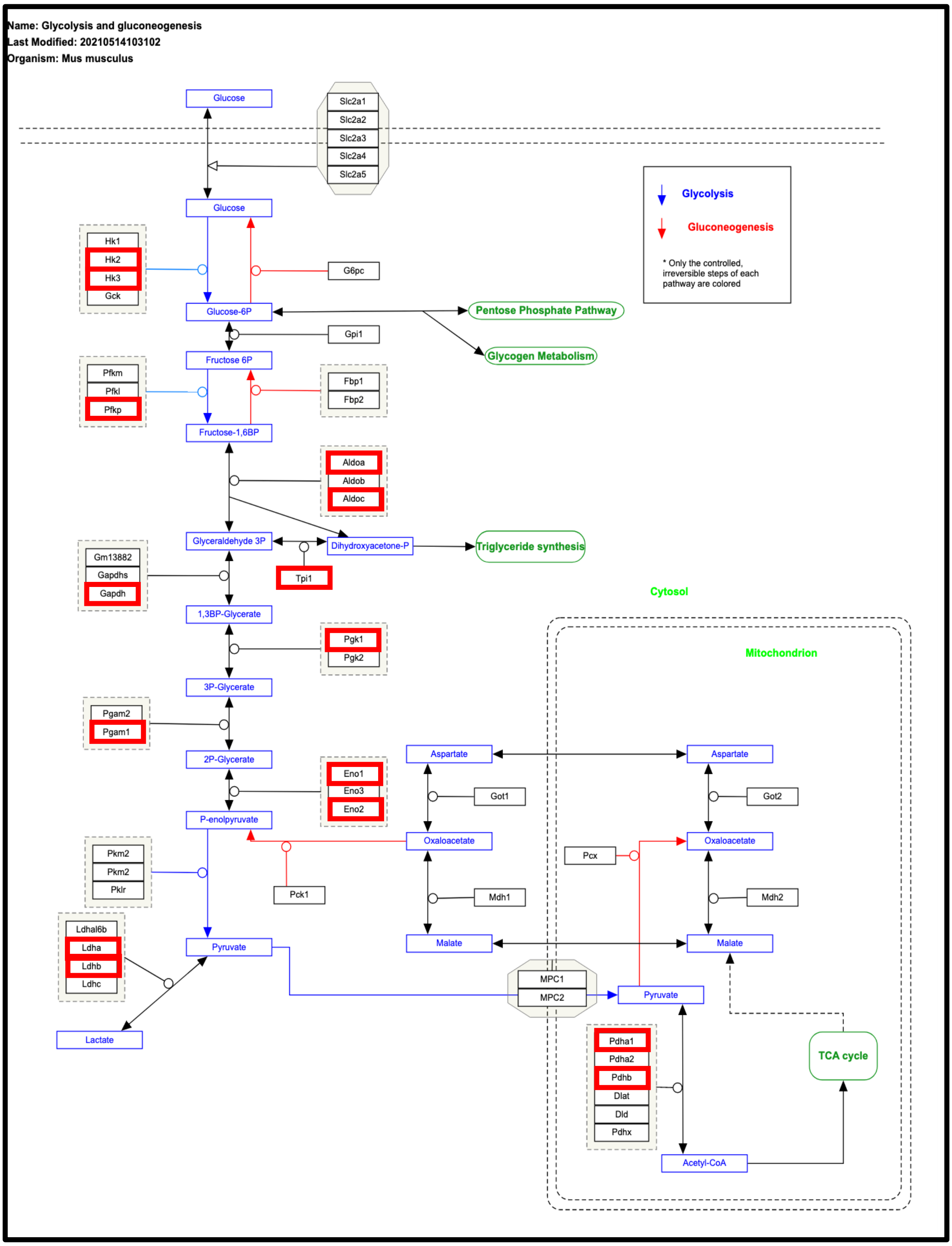

Supplementary Figure S2: Schematic representation of glycolysis pathway shows that 15 ‘leading edge’ genes of the GSEA of ‘glycolysis’ pathway constitute 9 out of 10 steps of glycolysis (boxed in red). [source: mus musculus, WikiPathway WP157]
