# Supplementary material for: Molecular Insights into Transcranial Direct Current Stimulation Effects: Metabolomics and Transcriptomics Analyses
Source: Cells. 2024 Jan 23;13(3):205. doi: 10.3390/cells13030205 (PMC10854682; doi:10.3390/cells13030205)
Supplement: Supplementary file 1 [file cells-13-00205-s001.zip › Supplementary Information (All Tables).pdf]

Supplementary tables:

Supplementary Table S1: Table of expression of 72 high-quality annotated metabolites**.** The abundance of metabolites across all samples was quantile normalized and transformed to a natural logarithmic scale. Differences in the abundance of metabolites between sham-control and tDCS groups were calculated with a two-tailed Student's t-test.

Name	Formula	Control_1	Control_2	Control_3	tDCS_1	tDCS_2	tDCS_3	Ttest	FC	Up/Down_tDCS
**Adenosine**	C10 H13 N5 O4	15.9007	16.3566	16.49	19.6964	18.7086	19.1061	0.00211478	0.053865415	Up_tDCS
**DL-Malic acid**	C4 H6 O5	20.879	20.846	20.7717	20.2966	20.2489	20.4289	0.00292836	1.661022428	Down_tDCS
**D-Glucose 6-phosphate**	C6 H13 O9 P	18.3839	18.4549	18.5677	18.8392	18.7417	18.8565	0.00864654	0.709188916	Up_tDCS
**D-Sphingosine**	C18 H37 N O2	17.8981	18.2827	17.8442	17.1741	17.3086	17.3836	0.02119158	2.053543149	Down_tDCS
**DL-3-Aminoisobutyric**	C4 H9 N O2	18.8897	18.8722	18.6618	19.0601	19.1473	19.0074	0.0484858	0.768203966	Up_tDCS
**L(-)-Carnitine**	C7 H15 N O3	19.8391	19.8617	20.3379	19.4076	19.5118	19.4415	0.07043848	1.749389144	Down_tDCS
**Y-Glutamylcysteine**	C8 H14 N2 O5 S	17.1661	17.4326	17.2422	17.0917	16.8659	17.095	0.07483166	1.300523228	Down_tDCS
**NP-004400**	C26 H36 O12	17.3512	17.116	17.1369	17.7287	17.3813	17.4334	0.08411504	0.731176791	Up_tDCS
**Acetyl-L-carnitine**	C9 H17 N O4	19.8264	19.4239	19.5061	19.3643	18.7554	18.5263	0.08833085	2.020745831	Down_tDCS
**L-Pyroglutamic acid**	C5 H7 N O3	17.8356	18.1251	17.793	17.8204	17.363	17.5102	0.11124201	1.423853127	Down_tDCS
**Taurine**	C2 H7 N O3 S	19.4252	19.5164	19.6706	20.3242	19.6529	20.1664	0.11568498	0.60023542	Up_tDCS
**Y-L-Glutamyl-L-glutamic acid**	C10 H16 N2 O7	19.4054	19.464	19.2818	19.9641	19.4434	19.9148	0.13255032	0.676808666	Up_tDCS
**Adenosine 5'-monophosphate**	C10 H14 N5 O7 P	17.0293	16.3391	17.0584	17.4702	17.2957	17.2588	0.14358745	0.587057016	Up_tDCS
**Choline**	C5 H13 N O	22.1467	22.1315	21.5578	21.8047	20.8728	21.3356	0.14793891	1.836080861	Down_tDCS
**DL-Alanine**	C3 H7 N O2	18.337	17.1895	17.3599	17.1857	16.3923	17.0031	0.15905525	2.156385269	Down_tDCS
**L-Isoleucine**	C6 H13 N O2	16.451	15.7534	18.9297	18.5786	19.4938	19.0201	0.16838396	0.137225004	Up_tDCS
**2'-Deoxyguanosine 5'-monophosphate**	C10 H14 N5 O7 P	16.9925	16.8298	16.7939	16.8571	16.6017	16.4853	0.16877958	1.251112722	Down_tDCS
**Adenosine diphosphate (ADP)**	C10 H15 N5 O10 P2	17.2588	16.8793	16.9578	16.2019	16.9943	16.492	0.16928184	1.598768001	Down_tDCS
**L-Histidine**	C6 H9 N3 O2	18.89	18.9465	18.8442	18.8107	17.9528	18.3733	0.1716169	1.673024947	Down_tDCS
**4-Oxoproline**	C5 H7 N O3	20.0907	19.7866	19.5455	18.0417	19.2863	19.6008	0.21660149	2.296378538	Down_tDCS
**L-Aspartic acid**	C4 H7 N O4	19.9004	19.7551	19.7575	19.7353	19.4628	19.7559	0.24538883	1.165324979	Down_tDCS
**Hypoxanthine**	C5 H4 N4 O	18.9226	18.9348	18.8595	20.2344	19.1126	19.0794	0.27187475	0.565619701	Up_tDCS
**Creatine**	C4 H9 N3 O2	23.1191	23.1168	23.1123	23.1252	22.6478	22.9691	0.28711147	1.223888804	Down_tDCS
**Oleamide**	C18 H35 N O	19.3986	19.4671	18.5369	19.6012	19.5402	19.4914	0.30370113	0.663606008	Up_tDCS
**Nicotinamide**	C6 H6 N2 O	20.9969	20.729	20.7596	19.7075	20.7204	20.7281	0.32005268	1.557631918	Down_tDCS
**D-(+)-Proline**	C5 H9 N O2	19.4577	19.133	19.1207	19.1494	18.8223	19.1964	0.32460829	1.198535027	Down_tDCS
**Citric acid**	C6 H8 O7	20.4706	20.3565	20.5136	18.3656	20.0269	20.4879	0.3309642	2.270726899	Down_tDCS
**Dodecyltrimethylammonium**	C15 H33 N	16.8675	18.2139	16.6165	17.4489	15.6271	16.267	0.34219854	2.192333862	Down_tDCS
**NP-001346**	C11 H15 N5 O3 S	17.3207	17.1287	17.1289	17.2619	16.7577	17.0561	0.37791492	1.182384702	Down_tDCS
**(2R,3S,4S,5R,6R)-2-(hydroxymethyl)-6-(2-phenylethoxy)oxane-3,4,5-triol**	C14 H20 O6	16.8485	16.0967	15.4632	16.5724	16.6722	16.5045	0.38043352	0.639607866	Up_tDCS
**Cytosine**	C4 H5 N3 O	17.7502	17.5474	17.4711	18.3176	17.6177	17.6095	0.38887885	0.772054605	Up_tDCS
**L-Glutathione oxidized**	C20 H32 N6 O12 S2	19.0321	19.1613	19.3769	19.3432	18.7008	18.8959	0.39986288	1.233842561	Down_tDCS
**N-Acetyl-1-aspartylglutamic acid**	C11 H16 N2 O8	19.0106	18.9835	19.0704	18.9738	18.5776	19.0486	0.40077951	1.167463368	Down_tDCS
**L-Glutathione**	C10 H17 N3 O6 S	18.8392	19.3916	19.7158	18.9627	19.298	18.858	0.40894422	1.317803936	Down_tDCS
**Crotonic acid**	C4 H6 O2	20.7261	20.7802	20.6736	20.7525	20.2764	20.6881	0.4103308	1.166840887	Down_tDCS
**6-Hydroxy-2-naphthoic acid**	C11 H8 O3	19.3794	19.3083	18.8726	19.7426	19.1148	19.2892	0.47112915	0.822478174	Up_tDCS
**2-Amino-1,3,4-octadecanetriol**	C18 H39 N O3	16.3535	16.6942	15.3368	16.0213	15.7841	15.4866	0.47428499	1.43931408	Down_tDCS
**2-Naphthylamine**	C10 H9 N	16.3473	16.3525	16.0781	16.4032	15.7638	16.117	0.48413652	1.178960754	Down_tDCS
**D-Sedoheptulose 7-phosphate**	C7 H15 O10 P	17.1201	17.081	17.4571	17.3038	16.2688	17.3111	0.54147598	1.29455456	Down_tDCS
**DL-Tryptophan**	C11 H12 N2 O2	19.8055	19.7066	19.5466	19.8831	19.305	19.4856	0.54521371	1.136931917	Down_tDCS
**Guanosine 5'-diphosphate (GDP)**	C10 H15 N5 O11 P2	16.7159	16.6002	17.2177	16.9598	16.232	16.7805	0.55392604	1.20582824	Down_tDCS
**L-Phenylalanine**	C9 H11 N O2	20.3263	20.1905	17.9886	20.2864	19.8401	19.9136	0.57110181	0.599555539	Up_tDCS
**N-α-L-Acetyl-arginine**	C8 H16 N4 O3	16.3433	16.5899	16.7867	16.706	16.5305	16.7503	0.58206336	0.914876069	Up_tDCS
**Guanosine monophosphate**	C10 H14 N5 O8 P	16.7915	16.4342	17.0291	17.3745	16.3998	17.0567	0.60340716	0.82525185	Up_tDCS
**Xanthine**	C5 H4 N4 O2	19.7438	19.9619	19.4426	20.2276	19.5631	19.7585	0.62055733	0.874910806	Up_tDCS
**DL-Lactic Acid**	C3 H6 O3	20.6738	20.3525	20.7132	20.6409	20.37	20.5176	0.64155732	1.072865744	Down_tDCS
**D-(-)-Glutamine**	C5 H10 N2 O3	20.3786	20.3225	20.2895	20.4686	19.8726	20.3756	0.67205914	1.095561116	Down_tDCS
**Palmitoylcarnitine**	C23 H45 N O4	18.465	18.2379	18.3243	18.4849	17.643	18.4957	0.6843899	1.144002792	Down_tDCS
**Coniine**	C8 H17 N	13.9623	13.9363	12.0622	14.6364	12.1508	12.0456	0.74090429	1.456447134	Down_tDCS
**Guanine**	C5 H5 N5 O	17.3025	17.297	17.2662	17.9397	16.9413	17.2884	0.76247398	0.90372214	Up_tDCS

**Uric acid**	C5 H4 N4 O3	16.2855	16.009	15.7115	16.7112	15.4275	15.4911	0.80079204	1.133601803	Down_tDCS
**Cytidine**	C9 H13 N3 O5	17.2488	17.2848	17.3674	17.4538	16.999	17.3346	0.81027895	1.038592745	Down_tDCS
**Uracil**	C4 H4 N2 O2	16.7489	16.712	16.6865	17.3919	16.3881	16.5974	0.82556609	0.926198535	Up_tDCS
**Xanthosine**	C10 H12 N4 O6	17.005	16.7061	16.5233	17.2707	16.3243	16.8478	0.83592379	0.932891229	Up_tDCS
**Acetylcholine**	C7 H15 N O2	18.7653	18.8133	19.1234	19.0507	18.6973	19.0622	0.83686889	0.964575986	Up_tDCS
**(+/-)11(12)-EET**	C20 H32 O3	15.4349	15.3103	15.0635	16.4735	14.5387	14.3135	0.83723908	1.174684969	Down_tDCS
**Adenine**	C5 H5 N5	15.2639	15.6203	15.3254	16.1946	14.8388	15.4191	0.85875559	0.922224432	Up_tDCS
**Bis(methylbenzylidene) sorbitol**	C22 H26 O6	20.1445	20.1221	19.1388	20.5301	19.4342	19.1369	0.86017128	1.106719241	Down_tDCS
**DL-4-Hydroxyphenyllactic**	C9 H10 O4	15.1409	15.043	14.7585	15.4414	14.7211	14.6417	0.88012955	1.047144218	Down_tDCS
**DL-Arginine**	C6 H14 N4 O2	18.7779	18.5299	18.2828	18.1415	18.9999	18.3078	0.88456089	1.048261768	Down_tDCS
**N-Acetyl-L-methionine**	C7 H13 N O3 S	15.6712	15.6444	15.2857	15.7522	15.4732	15.2951	0.88957003	1.027299314	Down_tDCS
**Uridine**	C9 H12 N2 O6	18.1286	18.0776	17.7343	18.8365	17.5057	17.7825	0.89623901	0.940446986	Up_tDCS
**N-Acetylaspartic acid**	C6 H9 N O5	17.678	17.4255	17.4144	17.7446	17.2814	17.4246	0.89756943	1.022686853	Down_tDCS
**Guanosine**	C10 H13 N5 O5	17.416	17.4099	17.41	17.9499	16.9957	17.409	0.89924805	0.961205871	Up_tDCS
**5-(6-hydroxy-6-methyloctyl)-2,5-dihydrofuran-2-one**	C13 H22 O3	17.6102	18.7698	16.9828	18.9841	17.0225	17.1384	0.93411491	1.075300331	Down_tDCS
**Bis(4-ethylbenzylidene)sorbi**	C24 H30 O6	18.8105	19.1488	17.6276	19.7065	18.2391	17.8337	0.9345923	0.937879941	Up_tDCS
**TKK**	C16 H33 N5 O5	19.1694	19.5131	19.1496	17.5722	18.5537	21.9752	0.95264331	0.91423588	Up_tDCS
**ent-Prostaglandin F2α**	C20 H34 O5	15.7105	15.7625	15.2397	16.0934	15.3129	15.2521	0.95784399	1.018264798	Down_tDCS
**Cannabidiolic acid**	C22 H30 O4	15.1839	16.8866	16.6599	15.2003	15.3068	18.0788	0.96718378	1.049345532	Down_tDCS
**Pencycuron**	C19 H21 Cl N2 O	17.3985	17.0391	16.8939	17.7647	16.8007	16.8122	0.96814503	0.984750798	Up_tDCS
**Oleoyl ethanolamide**	C20 H39 N O2	18.1503	18.046	17.7677	18.2085	17.7157	18.0223	0.97623933	1.00585038	Down_tDCS
**4-Guanidinobutyric**	C5 H11 N3 O2	17.4344	17.0957	17.0495	17.8178	17.0155	16.7252	0.98537725	1.007058125	Down_tDCS

Supplementary Table S2: Expression profiles depicting genes associated with the 'glycolysis' pathway.

Gene name	log2Fold Change	pvalue	padj	Control Sample16	Control Sample15	Control Sample13	250tDCS Sample8	250tDCS Sample7	250tDCS Sample6	250tDCS Sample5	Up/Down tDCS
**Eno4**	-1.11023	5.33E-01	NA	0.9815711	2.939825	0.9864765	1.013363	2.0155862	0	0	Down_tDCS
**Aldh3a1**	-0.37288	8.39E-01	NA	0.9815711	0.979942	0.9864765	0	1.0077931	1.012884	1.014138	Down_tDCS
**Aldh3b2**	-0.37253	3.53E-01	NA	14.723567	17.63895	17.756578	15.20045	12.093517	12.15461	12.16965	Down_tDCS
**Pklr**	-0.37193	8.10E-01	NA	0.9815711	1.959883	0.9864765	1.013363	1.0077931	1.012884	1.014138	Down_tDCS
**Pgam2**	-0.31117	6.04E-01	NA	6.8709979	7.839533	7.8918123	6.080179	6.0467587	6.077304	6.084827	Down_tDCS
**Galm**	-0.13921	7.00E-01	NA	18.649852	19.59883	17.756578	17.22718	16.12469	17.21903	17.24034	Down_tDCS
**Acss2**	-0.13675	6.13E-01	NA	33.373419	36.25784	35.513155	28.37417	34.264966	26.33498	38.53724	Down_tDCS
**Dlat**	-0.12855	5.69E-01	NA	46.133843	41.15755	51.29678	43.57462	42.327311	41.52825	41.57965	Down_tDCS
**Aldh1b1**	-0.11536	7.87E-01	NA	11.778854	14.69912	13.810671	13.17372	12.093517	13.16749	11.15552	Down_tDCS
**Gapdhs**	-0.08308	9.32E-01	NA	2.9447134	2.939825	2.9594296	3.04009	3.0233793	2.025768	3.042414	Down_tDCS
**Aldh3b1**	-0.07651	7.50E-01	NA	36.318132	58.79649	47.350874	54.72162	41.319518	42.54113	41.57965	Down_tDCS
**Aldh9a1**	-0.03495	8.80E-01	NA	43.18913	45.07731	38.472585	40.53453	43.335104	39.50248	41.57965	Down_tDCS
**Pck2**	-0.03151	9.11E-01	NA	28.465563	30.37819	29.594296	27.36081	29.226	31.3994	27.38172	Down_tDCS
**Aldh3a2**	-0.02962	9.00E-01	NA	39.262845	40.1776	41.432014	40.53453	37.288345	39.50248	40.56551	Down_tDCS
**Acss1**	0.00831	9.75E-01	NA	34.35499	40.1776	29.594296	33.44099	35.272759	33.42517	37.5231	Up_tDCS
**Adpgk**	0.00851	9.70E-01	NA	48.096986	47.0372	41.432014	46.61471	46.358483	38.48959	51.72103	Up_tDCS
**Minpp1**	0.00899	9.71E-01	NA	34.35499	35.2779	37.486108	35.46771	35.272759	36.46383	36.50896	Up_tDCS
**G6pc3**	0.02523	8.98E-01	NA	56.931126	56.83661	50.310303	55.73498	55.428621	55.70862	55.77758	Up_tDCS
**Dld**	0.02954	8.62E-01	NA	74.599406	68.59591	76.94517	79.04233	71.553311	70.90188	78.08861	Up_tDCS
**Pfkl**	0.05486	7.57E-01	NA	66.746837	65.65608	67.080404	65.86861	69.537725	74.95342	65.91896	Up_tDCS
**Pgm1**	0.07044	7.20E-01	NA	53.986412	57.81655	52.283256	58.77507	54.420828	55.70862	60.84827	Up_tDCS
**Eno3**	0.07098	8.94E-01	NA	7.8525691	8.819474	7.8918123	8.106906	10.077931	8.103072	8.113103	Up_tDCS
**Adh5**	0.09234	6.31E-01	NA	56.931126	57.81655	53.269733	59.78843	58.452001	61.78593	58.82	Up_tDCS
**Aldh7a1**	0.10367	6.07E-01	NA	52.02327	51.9369	49.323827	52.69489	54.420828	56.72151	55.77758	Up_tDCS
**Pdha1**	0.13549	3.41E-01	NA	103.06497	98.9741	100.62061	112.4833	107.83386	112.4301	110.541	Up_tDCS
**Bpgm**	0.14981	3.50E-01	NA	79.507262	82.31509	76.94517	82.08242	86.670208	89.13379	95.32896	Up_tDCS
**Pfkp**	0.1624	1.99E-01	2.83E-01	141.34624	126.4125	145.99853	152.0045	148.14559	160.0357	157.1914	Up_tDCS
**Pdhb**	0.16689	3.10E-01	NA	71.654693	76.43544	72.999264	83.09579	79.615656	85.08226	83.1593	Up_tDCS
**Hk2**	0.168	8.40E-01	NA	2.9447134	3.919766	3.9459061	3.04009	4.0311725	4.051536	5.070689	Up_tDCS
**Pfkm**	0.20976	6.13E-02	1.36E-01	172.75652	175.4095	173.61987	200.6459	193.49628	202.5768	207.8983	Up_tDCS
**RGD1564958**	0.21118	8.81E-01	NA	0.9815711	0.979942	1.9729531	1.013363	2.0155862	2.025768	1.014138	Up_tDCS
**Pgam1**	0.21242	1.85E-01	NA	74.599406	76.43544	78.918123	91.20269	86.670208	92.17245	85.18758	Up_tDCS
**Fbp2**	0.21387	9.20E-01	NA	0.9815711	0.979942	0	1.013363	1.0077931	0	1.014138	Up_tDCS
**Eno2**	0.26968	2.50E-03	1.20E-02	306.25019	313.5813	303.83477	378.9979	354.74318	372.7413	378.2734	Up_tDCS
**Ldha**	0.27192	1.82E-02	5.65E-02	160.97767	162.6703	166.71453	196.5925	187.44952	209.667	195.7286	Up_tDCS
**Pgk1**	0.28954	7.61E-03	2.82E-02	184.53537	195.0084	199.26826	228.0067	225.74566	249.1695	240.3507	Up_tDCS
**Gpi**	0.29169	3.57E-03	1.58E-02	220.85351	224.4066	221.95722	272.5947	266.05738	279.556	270.7748	Up_tDCS
**Akr1a1**	0.29464	4.75E-03	1.97E-02	202.20365	214.6072	198.28178	253.3408	243.88593	256.2597	252.5203	Up_tDCS
**Eno1**	0.3296	9.86E-04	5.92E-03	245.39278	278.3034	239.7138	313.1292	316.44704	337.2904	312.3545	Up_tDCS
**Tpi1**	0.35905	1.67E-04	1.43E-03	253.24535	271.4438	256.4839	323.2629	323.50159	349.445	339.7362	Up_tDCS
**Aldoa**	0.36811	1.51E-06	3.47E-05	479.00671	521.3289	495.21122	645.5124	619.79277	664.4519	643.9775	Up_tDCS
**Ldhb**	0.3759	5.78E-05	6.37E-04	284.65563	294.9624	267.33514	355.6905	354.74318	368.6898	386.3865	Up_tDCS
**Aldoc**	0.40285	6.45E-06	1.30E-04	326.86319	338.0798	298.90239	411.4255	432.34325	418.3211	437.0934	Up_tDCS
**Gapdh**	0.44484	7.46E-10	4.62E-08	603.66625	665.3803	605.69659	856.2919	829.41373	868.0416	848.8334	Up_tDCS
**Hk3**	0.76382	8.41E-01	NA	0	0	0	0	0	1.012884	0	Up_tDCS

Supplementary Table S3: Gene Set Enrichment Analysis result table depicting the significance of each studied pathway. NES stands for Normalized Enrichment Score. P-value is calculated based on the Kolmagorov-Smirnov statistical test comparing the deviation between the cumulative distributions of observed and expected gene ranks within the gene set. The 'leading edge' genes were defined as a subset of genes in the ranked list before the point where the running sum reaches its maximum deviation from zero.

Description	NES	p-value	adjusted p-value	Number of 'leading edge' genes	' leading edge ' genes
Glycolysis	1.04584	0.389899	0.389899	20	Gapdh, Aldoc, Ldhb, Aldoa, Tpi1, Eno1, Akr1a1, Gpi, Pgk1, Ldha, Eno2, Fbp2, Pgam1, RGD1564958, Pfkm, Hk2, Pdhb, Pfkp, Bpgm, Pdha1
Mitochondrial respirasome	2.629676	1.00E-10	9.28E-09	46	Ndufa13, Cox7a2, Ndufs5, Cox7b, Cox4i1, Ndufa4, Ndufb9, Cox7c, Cox6a1, Ndufb4, Uqcrh, Ndufa8, Cox5a, Ndufb2, Ndufa1, Ndufa6, Ndufb8, Cox4i2, Uqcrb, Ndufb11, Ndufb6, Ndufc2, Cox7a2l, Uqcrq, Ndufb7, Ndufb5, Ndufb10, Ndufv2, Ndufa3, Ndufa2, Ndufs3, Ndufa5, Sdhc, Ndufb3, Uqcrc1, Ndufs7, Uqcrc2, Sdhb, Ndufa7, Ndufa12, Uqcrfs1, Ndufab1, Ndufs8, Ndufs4, Sdhd, Ndufv1
ATP metabolic process	2.312705	1.00E-10	9.28E-09	50	Tspo, Cox7a2, Atp5j2, Cox4i1, Atp5l, Atp5b, Ndufb9, Cox7c, Cox6a1, Atp5j, Uqcrh, Cox5b, Atp5h, Atp5o, Ndufa8, Cox5a, Slc25a13, Atp5a1, Atp5e, Atp5d, Ndufb8, Cox4i2, Uqcrb, Ndufb6, Atp5g2, Chchd2, Ndufc2, Atpif1, Cox7a2l, Guk1, Uqcrq, Iscu, Fis1, Ndufv2, Atp5g1, Chchd10, Stoml2, Sdhc, Uqcrc1, Pink1, Atp5c1, Ndufa7, Gadd45gip1, Ndufa12, Pde2a, Uqcrfs1, Cycs, Ndufs8, Sdhd, Ndufv1
Respiratory chain complex	2.572259	1.00E-10	9.28E-09	46	Ndufa13, Cox6b1, Ndufs5, Cox7b, Cox4i1, Ndufa4, Ndufb9, Cox7c, Cox6a1, Ndufb4, Uqcrh, Ndufa8, Cox5a, Ndufb2, Ndufa1, Ndufa6, Cox8a, Ndufb8, Cox4i2, Uqcrb, Ndufb11, Ndufb6, Ndufc2, Uqcrq, Ndufb7, Ndufb5, Ndufb10, Ndufv2, Ndufa3, Ndufa2, Ndufs3, Ndufa5, Sdhc, Ndufb3, Uqcrc1, Ndufs7, Uqcrc2, Sdhb, Ndufa7, Ndufa12, Uqcrfs1, Ndufab1, Ndufs8, Ndufs4, Sdhd, Ndufv1
Oxidoreductase complex	2.241927	1.23E-09	7.71E-08	40	Ndufa13, Ndufs5, Ndufa4, Ndufb9, Ndufb4, Uqcrh, Ndufa8, Ndufb2, Ndufa1, Ndufa6, Ndufb8, Uqcrb, Ndufb11, Ndufb6, Ndufc2, Uqcrq, Ndufb7, Ndufb5, Ndufb10, Ndufv2, Mrps36, Ndufa3, Ndufa2, Ndufs3, Ndufa5, Sdhc, Ndufb3, Uqcrc1, Ndufs7, Uqcrc2, Sdhb, Ndufa7, Ndufa12, Pdhb, Uqcrfs1, Ndufab1, Ndufs8, Ndufs4, Sdhd, Ndufv1
Oxidative phosphorylation	2.241657	1.25E-09	7.71E-08	42	Cox7a2, Atp5j2, Cox4i1, Atp5l, Atp5b, Ndufb9, Cox7c, Cox6a1, Atp5j, Uqcrh, Cox5b, Atp5h, Atp5o, Ndufa8, Cox5a, Atp5a1, Atp5d, Ndufb8, Cox4i2, Uqcrb, Ndufb6, Chchd2, Ndufc2, Cox7a2l, Uqcrq, Iscu, Ndufv2, Chchd10, Stoml2, Sdhc, Uqcrc1, Pink1, Atp5c1, Ndufa7, Gadd45gip1, Ndufa12, Pde2a, Uqcrfs1, Cycs, Ndufs8, Sdhd, Ndufv1
TCA	1.516144	0.044648	0.0446483	18	Mdh1, Mdh2, Sdhc, Aco2, Sdhb, Suclg1, Pdhb, Idh3g, Sdhd, Pdha1, Suclg2, Sdha, Sucla2, Idh2, Cs, Idh3a, Idh1, Dlst
Response to external stimulus	1.804579	0.000149	0.0146109	42	Cxcl11, Cxcl10, Sell, Cxcl13, Cps1, Ghrh, Lilra5, Cxcl9, Ccl7, S100a9, Apobec1, Rgs1, Ccl4, Ccl2, Cav3, Ptger2, Wnt11, Ccl19, Ccr5, A2m, Fos, Ccl3, Myo1f, Trpm4, Gch1, Ptges, Trpv4, Cyba, Prok2, Aif1, Anxa3, Apoe, Actg1, Cd84, Cd40, Plcg2, S100a8, App, Calcrl, Lyn, Rab32, Pcdh15
Immune system process	1.820035	0.000153	0.0146109	40	Cxcl11, Cxcl10, Sell, Cxcl13, Cxcl9, Ccl7, S100a9, Apobec1, Ccl4, Add1, Ccl2, Ccl19, Ccr5, A2m, Fos, Ccl3, Myo1f, Trpm4, Gch1, Trpv4, Cyba, Aif1, Anxa3, Apoe, Actg1, Cd84, Cd40, Plcg2, S100a8, App, Myh9, Lyn, Rab32, Kcnn4, Lcp1, Thy1, Junb, Npy, Slc8a3, Prnp
Neurotransmitter secretion	-1.9281	0.000232	0.0146109	28	Mef2c, P2rx7, Chrna4, Prkce, Myo6, Slc30a1, Cacna1a, Chrnb2, Nlgn1, Stx2, Gsk3b, Syt2, Htr1b, Drd2, Adcy1, Otof, Unc13a, Cacnb4, Sphk1, Chrna7, Prkca, Htr2a, Cacna1b, Adra1a, Braf, Pclo, Hcrt, P2rx2
Synaptic membrane	-1.93671	0.000249	0.0146109	39	Kcnma1, Grm1, Trpv1, Slc30a1, Cacna1a, F2r, Chrnb2, Atp2b3, Nlgn1, Stx2, Cacng2, Grin2d, Itpr1, Akap5, Cacna2d1, Htr1b, Oprd1, Slc8a1, Drd2, Erbb3, Cdh9, Adcy1, Otof, Grm5, Unc13a, Grm6, Drd5, Cacna1h, Chrna7, Clstn2, Grin2b, Itsn1, Dgkb, Htr2a, Cacna1d, Il1rapl1, Cacna1c, Adra1a, P2rx2

Supplementary Table S4: Expression profiles of genes associated with the 'mitochondria functioning'. The genes belonging to the 'mitochondrial functioning' were identified utilizing the MitoCarta database.

Gene name	log2Fold Change	pvalue	padj	Control Sample16	Control Sample15	Control Sample13	250tDCS Sample8	250tDCS Sample7	250tDCS Sample6	250tDCS Sample5	Up/Down tDCS
Mutyh	-0.9041	3.95E-01	NA	3.926285	4.899708	3.9459061	0	5.038966	1.012884	3.042414	Down_tDCS
Lipt1	-0.6947	6.36E-01	NA	0.981571	1.959883	1.9729531	1.013363	1.007793	1.012884	1.014138	Down_tDCS
Fpgs	-0.5579	3.80E-01	NA	9.815711	5.879649	8.8782888	4.053453	9.070138	5.06442	4.056551	Down_tDCS
Neu4	-0.5526	2.97E-01	NA	10.79728	14.69912	7.8918123	9.120269	8.062345	5.06442	8.113103	Down_tDCS
Nudt13	-0.5243	6.07E-01	NA	2.944713	2.939825	3.9459061	3.04009	1.007793	3.038652	2.028276	Down_tDCS
Akr1b7	-0.3734	8.09E-01	NA	0.981571	0.979942	1.9729531	1.013363	1.007793	1.012884	1.014138	Down_tDCS
Cyct	-0.3734	8.09E-01	NA	0.981571	0.979942	1.9729531	1.013363	1.007793	1.012884	1.014138	Down_tDCS
Mettl4	-0.3734	8.09E-01	NA	0.981571	0.979942	1.9729531	1.013363	1.007793	1.012884	1.014138	Down_tDCS
Mettl8	-0.3733	6.39E-01	NA	5.889427	3.919766	4.9323827	3.04009	6.046759	3.038652	3.042414	Down_tDCS
Styxl1	-0.3732	7.66E-01	NA	1.963142	2.939825	1.9729531	2.026726	3.023379	2.025768	0	Down_tDCS
Ears2	-0.3731	6.65E-01	NA	3.926285	2.939825	5.9188592	4.053453	3.023379	2.025768	4.056551	Down_tDCS
Slc25a24	-0.3728	7.32E-01	NA	2.944713	1.959883	2.9594296	2.026726	2.015586	2.025768	2.028276	Down_tDCS
Aars2	-0.3727	8.01E-01	NA	0.981571	1.959883	1.9729531	2.026726	1.007793	2.025768	0	Down_tDCS
Mthfd2l	-0.3727	7.32E-01	NA	1.963142	2.939825	2.9594296	2.026726	2.015586	2.025768	2.028276	Down_tDCS
Slc25a16	-0.3724	5.42E-01	NA	5.889427	9.799416	7.8918123	4.053453	6.046759	7.090188	7.098965	Down_tDCS
Nags	-0.3724	7.90E-01	NA	0.981571	1.959883	1.9729531	1.013363	1.007793	1.012884	2.028276	Down_tDCS
Ehhadh	-0.3723	6.87E-01	NA	3.926285	3.919766	2.9594296	2.026726	3.023379	3.038652	3.042414	Down_tDCS
Star	-0.3719	8.10E-01	NA	0.981571	1.959883	0.9864765	1.013363	1.007793	1.012884	1.014138	Down_tDCS
Spata20	-0.3714	8.40E-01	NA	0.981571	0.979942	0.9864765	1.013363	0	1.012884	1.014138	Down_tDCS
Fdxr	-0.3307	5.09E-01	NA	11.77885	8.819474	12.824195	9.120269	6.046759	11.14172	9.127241	Down_tDCS
Mars2	-0.3295	5.07E-01	NA	10.79728	10.77936	10.851242	10.13363	9.070138	7.090188	8.113103	Down_tDCS
Pmaip1	-0.3252	5.35E-01	NA	10.79728	9.799416	8.8782888	9.120269	8.062345	8.103072	6.084827	Down_tDCS
Pars2	-0.3221	5.53E-01	NA	7.852569	8.819474	10.851242	7.093543	7.054552	7.090188	8.113103	Down_tDCS
Pdp2	-0.3138	5.94E-01	NA	7.852569	6.859591	8.8782888	7.093543	6.046759	6.077304	6.084827	Down_tDCS
Txnrd1	-0.3019	4.10E-01	NA	17.66828	21.55871	19.729531	16.21381	13.10131	17.21903	17.24034	Down_tDCS
Pde12	-0.2903	6.83E-01	NA	5.889427	4.899708	5.9188592	5.066816	5.038966	4.051536	4.056551	Down_tDCS
Cox8b	-0.29	6.84E-01	NA	4.907856	5.879649	5.9188592	5.066816	4.031172	5.06442	4.056551	Down_tDCS
Slc25a10	-0.2884	2.85E-01	NA	38.28127	45.07731	31.567249	32.42762	32.24938	25.3221	35.49482	Down_tDCS
Usp30	-0.2828	2.98E-01	NA	39.26285	33.31801	34.526679	29.38753	33.25717	24.30922	30.42414	Down_tDCS
Polg	-0.2713	4.71E-01	NA	18.64985	16.65901	18.743054	14.18709	15.1169	15.19326	15.21207	Down_tDCS
Pdpr	-0.2696	6.22E-01	NA	8.83414	7.839533	9.8647653	7.093543	7.054552	7.090188	8.113103	Down_tDCS
Slc25a37	-0.2656	7.47E-01	NA	3.926285	4.899708	3.9459061	4.053453	4.031172	3.038652	3.042414	Down_tDCS
D2hgdh	-0.2635	5.01E-01	NA	16.68671	17.63895	15.783625	12.16036	14.1091	14.18038	15.21207	Down_tDCS
Akap10	-0.2573	5.83E-01	NA	11.77885	9.799416	13.810671	10.13363	10.07793	9.115956	10.14138	Down_tDCS
Bcl2l13	-0.2472	4.71E-01	NA	21.59456	19.59883	23.675437	17.22718	19.14807	18.23191	18.25448	Down_tDCS
Bcl2l1	-0.2427	2.31E-01	NA	58.89427	59.77644	69.053357	53.70825	47.36628	63.81169	46.65034	Down_tDCS
Lars2	-0.2395	6.36E-01	NA	10.79728	8.819474	10.851242	9.120269	9.070138	9.115956	7.098965	Down_tDCS
Oxsm	-0.2382	5.83E-01	NA	13.742	13.71918	12.824195	12.16036	12.09352	10.12884	11.15552	Down_tDCS
Stx17	-0.2357	7.18E-01	NA	5.889427	4.899708	8.8782888	5.066816	6.046759	6.077304	5.070689	Down_tDCS
Sphkap	-0.2353	4.88E-01	NA	21.59456	19.59883	27.621343	21.28063	19.14807	20.25768	17.24034	Down_tDCS
Nudt6	-0.2351	6.02E-01	NA	12.76042	13.71918	12.824195	12.16036	13.10131	7.090188	12.16965	Down_tDCS
Prss35	-0.2345	8.06E-01	NA	2.944713	3.919766	2.9594296	4.053453	2.015586	2.025768	3.042414	Down_tDCS
Myo19	-0.2284	7.29E-01	NA	5.889427	5.879649	6.9053357	5.066816	6.046759	5.06442	5.070689	Down_tDCS
Mrps27	-0.2236	4.43E-01	NA	29.44713	31.35813	29.594296	23.30735	25.19483	24.30922	30.42414	Down_tDCS
Wars2	-0.2206	7.45E-01	NA	6.870998	4.899708	5.9188592	5.066816	5.038966	5.06442	5.070689	Down_tDCS
Hpdl	-0.2122	7.62E-01	NA	4.907856	5.879649	5.9188592	5.066816	5.038966	4.051536	5.070689	Down_tDCS
Gpd2	-0.2093	5.95E-01	NA	15.70514	15.67907	17.756578	12.16036	16.12469	15.19326	13.18379	Down_tDCS
Pusl1	-0.2091	5.94E-01	NA	17.66828	15.67907	15.783625	15.20045	15.1169	12.15461	14.19793	Down_tDCS
Rpusd3	-0.2089	5.92E-01	NA	16.68671	16.65901	15.783625	15.20045	13.10131	14.18038	14.19793	Down_tDCS
Nlrx1	-0.2076	6.68E-01	NA	10.79728	9.799416	11.837718	10.13363	9.070138	9.115956	9.127241	Down_tDCS
Tmem143	-0.2026	5.34E-01	NA	24.53928	22.53866	23.675437	22.29399	19.14807	20.25768	20.28276	Down_tDCS
Pycr1	-0.1974	6.93E-01	NA	9.815711	9.799416	10.851242	10.13363	8.062345	8.103072	9.127241	Down_tDCS
Bcl2l11	-0.1924	8.01E-01	NA	5.889427	2.939825	5.9188592	4.053453	4.031172	5.06442	4.056551	Down_tDCS
Ide	-0.1883	6.52E-01	NA	12.76042	14.69912	15.783625	11.147	14.1091	12.15461	13.18379	Down_tDCS
Ogg1	-0.185	6.89E-01	NA	9.815711	12.73924	12.824195	11.147	10.07793	10.12884	10.14138	Down_tDCS
Gdap1	-0.1816	4.82E-01	NA	36.31813	34.29795	40.445538	36.48108	31.24159	31.3994	31.43827	Down_tDCS

**Rdh13**	-0.1801	7.00E-01	NA	10.79728	10.77936	12.824195	9.120269	11.08572	10.12884	10.14138	Down_tDCS
**Acaca**	-0.1801	6.25E-01	NA	17.66828	16.65901	20.716007	16.21381	17.13248	15.19326	16.22621	Down_tDCS
**Dnajc30**	-0.1799	5.34E-01	NA	30.42871	29.39825	29.594296	29.38753	26.20262	23.29633	26.36758	Down_tDCS
**Acacb**	-0.1795	8.78E-01	NA	2.944713	2.939825	0.9864765	1.013363	3.023379	2.025768	2.028276	Down_tDCS
**Gcat**	-0.1738	6.23E-01	NA	21.59456	20.57877	17.756578	16.21381	17.13248	19.2448	18.25448	Down_tDCS
**Trub2**	-0.1732	7.46E-01	NA	8.83414	8.819474	8.8782888	8.106906	8.062345	7.090188	8.113103	Down_tDCS
**Bcs1l**	-0.1718	6.08E-01	NA	21.59456	21.55871	22.68896	19.2539	18.14028	21.27056	19.26862	Down_tDCS
**Abcb7**	-0.1712	6.96E-01	NA	11.77885	11.7593	15.783625	12.16036	12.09352	11.14172	11.15552	Down_tDCS
**Ccdc127**	-0.1639	6.03E-01	NA	24.53928	22.53866	28.60782	21.28063	24.18703	22.28345	22.31103	Down_tDCS
**Dnajc28**	-0.1632	7.38E-01	NA	9.815711	9.799416	11.837718	9.120269	9.070138	10.12884	9.127241	Down_tDCS
**Gpt2**	-0.1611	6.10E-01	NA	24.53928	26.45842	23.675437	20.26726	25.19483	22.28345	21.29689	Down_tDCS
**Ppm1k**	-0.1606	5.56E-01	NA	31.41028	31.35813	36.499632	31.41426	30.23379	29.37364	27.38172	Down_tDCS
**Aldh4a1**	-0.1584	6.84E-01	NA	15.70514	17.63895	15.783625	16.21381	16.12469	13.16749	13.18379	Down_tDCS
**Spryd4**	-0.1583	6.82E-01	NA	16.68671	16.65901	15.783625	14.18709	14.1091	16.20614	14.19793	Down_tDCS
**Arg2**	-0.1569	7.51E-01	NA	9.815711	9.799416	10.851242	9.120269	9.070138	8.103072	10.14138	Down_tDCS
**Cyp11b1**	-0.1536	6.59E-01	NA	17.66828	20.57877	27.621343	23.30735	20.15586	17.21903	18.25448	Down_tDCS
**Mtfr1**	-0.1506	8.59E-01	NA	2.944713	3.919766	4.9323827	4.053453	4.031172	3.038652	3.042414	Down_tDCS
**Pdp1**	-0.1503	5.41E-01	NA	36.31813	39.19766	48.33735	40.53453	35.27276	38.48959	34.48069	Down_tDCS
**Clybl**	-0.1503	6.58E-01	NA	19.63142	22.53866	22.68896	20.26726	21.16366	20.25768	16.22621	Down_tDCS
**Cry1**	-0.1503	7.66E-01	NA	8.83414	9.799416	10.851242	8.106906	9.070138	8.103072	10.14138	Down_tDCS
**Adck1**	-0.1502	7.03E-01	NA	15.70514	14.69912	16.770101	14.18709	14.1091	14.18038	14.19793	Down_tDCS
**Ddx28**	-0.1502	7.03E-01	NA	15.70514	15.67907	15.783625	14.18709	14.1091	15.19326	13.18379	Down_tDCS
**Aifm2**	-0.1501	7.92E-01	NA	7.852569	7.839533	7.8918123	7.093543	7.054552	6.077304	8.113103	Down_tDCS
**Dhodh**	-0.1501	7.92E-01	NA	6.870998	8.819474	7.8918123	7.093543	7.054552	7.090188	7.098965	Down_tDCS
**Cox11**	-0.147	6.64E-01	NA	20.61299	21.55871	21.702484	19.2539	19.14807	19.2448	19.26862	Down_tDCS
**Nt5dc3**	-0.1465	5.84E-01	NA	32.39185	31.35813	40.445538	33.44099	32.24938	29.37364	30.42414	Down_tDCS
**Acot2**	-0.1441	7.56E-01	NA	10.79728	12.73924	10.851242	10.13363	10.07793	10.12884	11.15552	Down_tDCS
**Acadsb**	-0.1438	6.15E-01	NA	30.42871	28.41831	32.553726	26.34744	32.24938	25.3221	26.36758	Down_tDCS
**Mrs2**	-0.1431	6.89E-01	NA	18.64985	18.61889	19.729531	17.22718	17.13248	18.23191	16.22621	Down_tDCS
**Nars2**	-0.1431	7.80E-01	NA	9.815711	8.819474	9.8647653	8.106906	8.062345	9.115956	9.127241	Down_tDCS
**Vars2**	-0.143	6.90E-01	NA	20.61299	18.61889	17.756578	17.22718	18.14028	16.20614	17.24034	Down_tDCS
**Fahd1**	-0.1419	6.54E-01	NA	25.52085	24.49854	23.675437	24.32072	23.17924	22.28345	19.26862	Down_tDCS
**Vps13d**	-0.1412	7.25E-01	NA	14.72357	14.69912	15.783625	13.17372	13.10131	14.18038	14.19793	Down_tDCS
**Sars2**	-0.1403	6.83E-01	NA	19.63142	22.53866	19.729531	19.2539	19.14807	18.23191	18.25448	Down_tDCS
**Fastkd5**	-0.1381	8.41E-01	NA	4.907856	5.879649	5.9188592	5.066816	5.038966	5.06442	5.070689	Down_tDCS
**Cmc1**	-0.1364	7.12E-01	NA	15.70514	27.43836	17.756578	20.26726	15.1169	17.21903	21.29689	Down_tDCS
**Fhit**	-0.1346	6.09E-01	NA	35.33656	36.25784	32.553726	29.38753	33.25717	33.42517	30.42414	Down_tDCS
**Hemk1**	-0.1344	7.59E-01	NA	12.76042	12.73924	12.824195	13.17372	11.08572	12.15461	10.14138	Down_tDCS
**Afg3l1**	-0.1333	7.02E-01	NA	19.63142	20.57877	19.729531	19.2539	18.14028	18.23191	17.24034	Down_tDCS
**Adhfe1**	-0.1323	5.85E-01	NA	39.26285	46.05725	39.459061	33.44099	40.31172	35.45094	42.59379	Down_tDCS
**Nt5dc2**	-0.1319	7.86E-01	NA	9.815711	8.819474	13.810671	9.120269	9.070138	12.15461	9.127241	Down_tDCS
**Stom**	-0.1315	8.25E-01	NA	6.870998	7.839533	6.9053357	6.080179	7.054552	7.090188	6.084827	Down_tDCS
**Sphk2**	-0.1315	6.09E-01	NA	37.2997	35.2779	35.513155	33.44099	32.24938	31.3994	34.48069	Down_tDCS
**Car5a**	-0.1315	8.81E-01	NA	2.944713	3.919766	3.9459061	3.04009	3.023379	3.038652	4.056551	Down_tDCS
**Slc25a34**	-0.1315	7.84E-01	NA	10.79728	10.77936	10.851242	12.16036	9.070138	9.115956	9.127241	Down_tDCS
**Tfb2m**	-0.1314	6.95E-01	NA	22.57614	22.53866	19.729531	20.26726	19.14807	20.25768	19.26862	Down_tDCS
**Trmu**	-0.1293	7.41E-01	NA	15.70514	17.63895	14.797148	14.18709	17.13248	14.18038	13.18379	Down_tDCS
**Hccs**	-0.1287	6.80E-01	NA	24.53928	23.5186	26.634866	22.29399	22.17145	23.29633	23.32517	Down_tDCS
**Thg1l**	-0.1286	7.72E-01	NA	10.79728	13.71918	12.824195	11.147	12.09352	10.12884	12.16965	Down_tDCS
**Dlat**	-0.1285	5.69E-01	NA	46.13384	41.15755	51.29678	43.57462	42.32731	41.52825	41.57965	Down_tDCS
**Nme4**	-0.1283	4.51E-01	NA	75.58098	75.4555	77.931646	68.9087	74.57669	66.85035	68.96137	Down_tDCS
**Thnsl1**	-0.1272	8.12E-01	NA	9.815711	8.819474	7.8918123	7.093543	7.054552	10.12884	8.113103	Down_tDCS
**Cbr4**	-0.1266	7.00E-01	NA	22.57614	23.5186	22.68896	21.28063	18.14028	20.25768	24.33931	Down_tDCS
**Coq4**	-0.1258	7.23E-01	NA	17.66828	21.55871	18.743054	18.24054	17.13248	18.23191	17.24034	Down_tDCS
**Mrm1**	-0.1247	7.96E-01	NA	9.815711	10.77936	10.851242	9.120269	10.07793	9.115956	10.14138	Down_tDCS
**Acsl1**	-0.1247	6.53E-01	NA	32.39185	29.39825	32.553726	27.36081	29.226	27.34787	31.43827	Down_tDCS
**Aldh5a1**	-0.1236	5.87E-01	NA	46.13384	45.07731	44.391444	40.53453	46.35848	39.50248	39.55138	Down_tDCS
**Mipep**	-0.1203	7.16E-01	NA	20.61299	21.55871	24.661913	20.26726	20.15586	21.27056	20.28276	Down_tDCS
**Slc25a44**	-0.1177	6.85E-01	NA	30.42871	28.41831	27.621343	29.38753	26.20262	26.33498	24.33931	Down_tDCS
**Polrmt**	-0.1173	7.35E-01	NA	21.59456	20.57877	18.743054	19.2539	20.15586	18.23191	17.24034	Down_tDCS
**Aldh1b1**	-0.1154	7.87E-01	NA	11.77885	14.69912	13.810671	13.17372	12.09352	13.16749	11.15552	Down_tDCS
**Bcl2**	-0.1098	9.06E-01	NA	3.926285	2.939825	2.9594296	2.026726	4.031172	3.038652	3.042414	Down_tDCS
**Cdk5rap1**	-0.1096	7.66E-01	NA	15.70514	17.63895	20.716007	16.21381	16.12469	17.21903	17.24034	Down_tDCS
**Nt5m**	-0.1093	7.48E-01	NA	20.61299	24.49854	18.743054	20.26726	20.15586	18.23191	20.28276	Down_tDCS
**Opa3**	-0.1073	6.68E-01	NA	38.28127	37.23778	36.499632	35.46771	34.26497	33.42517	35.49482	Down_tDCS
**Abcd2**	-0.1055	7.66E-01	NA	17.66828	18.61889	21.702484	18.24054	17.13248	18.23191	18.25448	Down_tDCS
**Mthfd1l**	-0.1051	7.76E-01	NA	17.66828	17.63895	17.756578	17.22718	17.13248	16.20614	15.21207	Down_tDCS
**Aifm3**	-0.1049	7.96E-01	NA	16.68671	14.69912	16.770101	9.120269	18.14028	15.19326	17.24034	Down_tDCS
**Lyplal1**	-0.1046	7.87E-01	NA	15.70514	16.65901	15.783625	15.20045	15.1169	16.20614	13.18379	Down_tDCS
**Mrrf**	-0.1041	7.99E-01	NA	13.742	14.69912	14.797148	13.17372	13.10131	14.18038	13.18379	Down_tDCS

**Tbrg4**	-0.104	7.24E-01	NA	31.41028	27.43836	27.621343	31.41426	27.21041	22.28345	26.36758	Down_tDCS
**Coasy**	-0.1024	7.01E-01	NA	35.33656	34.29795	30.580773	30.4009	32.24938	30.38652	31.43827	Down_tDCS
**Top1mt**	-0.1019	7.65E-01	NA	19.63142	21.55871	20.716007	18.24054	20.15586	19.2448	19.26862	Down_tDCS
**Nthl1**	-0.101	8.43E-01	NA	9.815711	10.77936	7.8918123	8.106906	9.070138	9.115956	9.127241	Down_tDCS
**Tars2**	-0.1006	7.91E-01	NA	14.72357	16.65901	20.716007	14.18709	15.1169	17.21903	18.25448	Down_tDCS
**Macrod1**	-0.1002	7.92E-01	NA	17.66828	20.57877	13.810671	17.22718	17.13248	14.18038	16.22621	Down_tDCS
**Fastkd3**	-0.0984	8.11E-01	NA	13.742	13.71918	14.797148	13.17372	13.10131	13.16749	13.18379	Down_tDCS
**Trit1**	-0.0982	8.13E-01	NA	12.76042	15.67907	13.810671	13.17372	12.09352	14.18038	13.18379	Down_tDCS
**Slc25a27**	-0.0969	7.54E-01	NA	24.53928	24.49854	25.64839	24.32072	23.17924	23.29633	22.31103	Down_tDCS
**Bcat2**	-0.0954	8.01E-01	NA	15.70514	19.59883	15.783625	15.20045	14.1091	17.21903	17.24034	Down_tDCS
**Cryz**	-0.095	8.40E-01	NA	10.79728	10.77936	10.851242	10.13363	10.07793	10.12884	10.14138	Down_tDCS
**Mocs1**	-0.0949	8.41E-01	NA	10.79728	11.7593	9.8647653	9.120269	11.08572	10.12884	10.14138	Down_tDCS
**Cat**	-0.0944	6.52E-01	NA	57.9127	52.91684	46.364397	47.62807	49.38186	49.63132	49.69275	Down_tDCS
**Ndufaf1**	-0.0908	8.12E-01	NA	15.70514	15.67907	18.743054	17.22718	14.1091	15.19326	16.22621	Down_tDCS
**Osgepl1**	-0.0903	8.39E-01	NA	11.77885	11.7593	12.824195	10.13363	12.09352	12.15461	11.15552	Down_tDCS
**Hint3**	-0.0902	7.74E-01	NA	23.55771	23.5186	25.64839	22.29399	22.17145	22.28345	24.33931	Down_tDCS
**Tmem177**	-0.0902	8.40E-01	NA	10.79728	12.73924	12.824195	12.16036	11.08572	11.14172	11.15552	Down_tDCS
**Pptc7**	-0.0888	7.52E-01	NA	30.42871	28.41831	31.567249	28.37417	29.226	28.36075	27.38172	Down_tDCS
**Flad1**	-0.0881	7.76E-01	NA	26.50242	25.47848	24.661913	20.26726	25.19483	27.34787	23.32517	Down_tDCS
**Yme1l1**	-0.0879	7.32E-01	NA	36.31813	32.33807	39.459061	34.45435	32.24938	35.45094	33.46655	Down_tDCS
**Mtfmt**	-0.0871	7.97E-01	NA	19.63142	21.55871	21.702484	19.2539	18.14028	21.27056	20.28276	Down_tDCS
**Letm1**	-0.0865	6.82E-01	NA	51.0417	47.0372	54.256209	48.64144	49.38186	47.60555	45.6362	Down_tDCS
**Ccdc90b**	-0.0851	8.09E-01	NA	19.63142	19.59883	18.743054	17.22718	17.13248	19.2448	19.26862	Down_tDCS
**Cpox**	-0.0849	7.96E-01	NA	24.53928	21.55871	20.716007	21.28063	21.16366	20.25768	21.29689	Down_tDCS
**Dna2**	-0.0832	9.32E-01	NA	2.944713	2.939825	2.9594296	3.04009	3.023379	3.038652	2.028276	Down_tDCS
**Sirt4**	-0.0831	9.00E-01	NA	5.889427	5.879649	5.9188592	5.066816	6.046759	5.06442	6.084827	Down_tDCS
**Lypla1**	-0.078	8.33E-01	NA	17.66828	16.65901	17.756578	16.21381	15.1169	17.21903	17.24034	Down_tDCS
**Crot**	-0.0773	8.50E-01	NA	12.76042	13.71918	16.770101	13.17372	13.10131	15.19326	13.18379	Down_tDCS
**Acaa1a**	-0.0755	7.34E-01	NA	47.11541	46.05725	44.391444	41.54789	44.3429	41.52825	46.65034	Down_tDCS
**Pdk1**	-0.0739	7.83E-01	NA	30.42871	34.29795	33.540202	29.38753	30.23379	32.41229	32.45241	Down_tDCS
**Clpb**	-0.0731	7.66E-01	NA	37.2997	36.25784	41.432014	37.49444	36.28055	35.45094	36.50896	Down_tDCS
**Slc25a35**	-0.0729	9.26E-01	NA	4.907856	3.919766	3.9459061	4.053453	4.031172	4.051536	4.056551	Down_tDCS
**Stard7**	-0.0724	7.37E-01	NA	49.07856	46.05725	49.323827	44.58798	47.36628	45.57978	45.6362	Down_tDCS
**Ppif**	-0.0718	8.09E-01	NA	26.50242	28.41831	25.64839	25.33408	25.19483	25.3221	26.36758	Down_tDCS
**Pitrm1**	-0.0712	7.91E-01	NA	32.39185	31.35813	33.540202	31.41426	31.24159	31.3994	29.41	Down_tDCS
**Opa1**	-0.0712	7.72E-01	NA	34.35499	39.19766	53.269733	40.53453	38.29614	42.54113	39.55138	Down_tDCS
**Mul1**	-0.0705	8.23E-01	NA	23.55771	24.49854	23.675437	22.29399	23.17924	23.29633	22.31103	Down_tDCS
**Acad11**	-0.07	8.88E-01	NA	9.815711	9.799416	9.8647653	8.106906	10.07793	9.115956	10.14138	Down_tDCS
**Them4**	-0.0691	8.02E-01	NA	29.44713	31.35813	31.567249	29.38753	28.21821	30.38652	29.41	Down_tDCS
**Dbt**	-0.0677	8.57E-01	NA	17.66828	15.67907	16.770101	16.21381	16.12469	15.19326	16.22621	Down_tDCS
**Hdhd3**	-0.0667	8.34E-01	NA	23.55771	25.47848	21.702484	21.28063	24.18703	22.28345	22.31103	Down_tDCS
**Cyb5b**	-0.0659	7.91E-01	NA	37.2997	36.25784	38.472585	36.48108	35.27276	35.45094	35.49482	Down_tDCS
**Clpx**	-0.0659	8.31E-01	NA	24.53928	24.49854	25.64839	23.30735	23.17924	25.3221	23.32517	Down_tDCS
**Gpam**	-0.0659	7.91E-01	NA	39.26285	36.25784	36.499632	36.48108	37.28835	33.42517	35.49482	Down_tDCS
**Nsun4**	-0.0658	8.80E-01	NA	11.77885	12.73924	12.824195	11.147	11.08572	12.15461	13.18379	Down_tDCS
**Rpusd4**	-0.0644	8.77E-01	NA	12.76042	14.69912	13.810671	12.16036	13.10131	12.15461	15.21207	Down_tDCS
**Slc25a40**	-0.0643	9.15E-01	NA	6.870998	6.859591	6.9053357	6.080179	6.046759	7.090188	7.098965	Down_tDCS
**Akap1**	-0.0628	8.18E-01	NA	30.42871	31.35813	32.553726	29.38753	32.24938	29.37364	29.41	Down_tDCS
**Rars2**	-0.0622	9.09E-01	NA	8.83414	7.839533	7.8918123	7.093543	8.062345	8.103072	8.113103	Down_tDCS
**Mtpap**	-0.0622	8.70E-01	NA	15.70514	16.65901	16.770101	15.20045	15.1169	16.20614	16.22621	Down_tDCS
**Armcx1**	-0.0618	8.14E-01	NA	33.37342	32.33807	36.499632	31.41426	31.24159	33.42517	34.48069	Down_tDCS
**Ccdc51**	-0.0614	8.67E-01	NA	16.68671	17.63895	18.743054	17.22718	16.12469	17.21903	17.24034	Down_tDCS
**Pank2**	-0.0595	8.58E-01	NA	21.59456	19.59883	23.675437	21.28063	20.15586	20.25768	21.29689	Down_tDCS
**Mrpl50**	-0.0589	8.55E-01	NA	22.57614	23.5186	22.68896	20.26726	22.17145	23.29633	22.31103	Down_tDCS
**Aldh18a1**	-0.0581	8.48E-01	NA	24.53928	25.47848	26.634866	24.32072	25.19483	24.30922	24.33931	Down_tDCS
**Exd2**	-0.0567	7.95E-01	NA	46.13384	47.0372	47.350874	45.60135	45.35069	44.5669	44.62207	Down_tDCS
**Cox16**	-0.0547	8.05E-01	NA	45.15227	46.05725	48.33735	41.54789	42.32731	49.63132	45.6362	Down_tDCS
**Slc25a42**	-0.0544	8.61E-01	NA	24.53928	24.49854	26.634866	27.36081	24.18703	20.25768	25.35345	Down_tDCS
**Hsdl2**	-0.054	8.75E-01	NA	19.63142	31.35813	20.716007	18.24054	19.14807	27.34787	27.38172	Down_tDCS
**Gfm1**	-0.0537	8.43E-01	NA	30.42871	31.35813	33.540202	29.38753	31.24159	30.38652	31.43827	Down_tDCS
**Cox15**	-0.0535	8.44E-01	NA	32.39185	33.31801	29.594296	29.38753	31.24159	30.38652	31.43827	Down_tDCS
**Nat8l**	-0.0533	6.83E-01	7.16E-01	139.3831	134.252	135.14729	141.8709	124.9663	124.5847	133.8662	Down_tDCS
**Grpel2**	-0.0507	9.05E-01	NA	12.76042	12.73924	13.810671	13.17372	13.10131	12.15461	12.16965	Down_tDCS
**Lig3**	-0.0507	9.10E-01	NA	12.76042	10.77936	11.837718	11.147	12.09352	10.12884	12.16965	Down_tDCS
**Slc25a32**	-0.0507	9.21E-01	NA	7.852569	9.799416	9.8647653	9.120269	9.070138	8.103072	9.127241	Down_tDCS
**Coq6**	-0.0507	8.36E-01	NA	36.31813	38.21772	39.459061	36.48108	37.28835	35.45094	37.5231	Down_tDCS
**Sco1**	-0.0507	8.84E-01	NA	17.66828	20.57877	20.716007	19.2539	19.14807	18.23191	19.26862	Down_tDCS
**Fech**	-0.0506	8.73E-01	NA	22.57614	24.49854	23.675437	22.29399	23.17924	23.29633	22.31103	Down_tDCS
**Rnaseh1**	-0.0506	9.01E-01	NA	13.742	15.67907	13.810671	13.17372	15.1169	14.18038	13.18379	Down_tDCS
**Shmt2**	-0.0506	8.74E-01	NA	24.53928	22.53866	23.675437	20.26726	22.17145	22.28345	26.36758	Down_tDCS

**Mtx1**	-0.0506	8.97E-01	NA	14.72357	15.67907	16.770101	16.21381	13.10131	16.20614	15.21207	Down_tDCS
**Guf1**	-0.0505	9.28E-01	NA	7.852569	7.839533	7.8918123	7.093543	7.054552	8.103072	8.113103	Down_tDCS
**Atpaf2**	-0.0505	8.54E-01	NA	29.44713	33.31801	31.567249	29.38753	28.21821	31.3994	32.45241	Down_tDCS
**Lipt2**	-0.0505	9.43E-01	NA	5.889427	4.899708	4.9323827	5.066816	5.038966	4.051536	6.084827	Down_tDCS
**Apool**	-0.0505	9.35E-01	NA	5.889427	6.859591	6.9053357	7.093543	5.038966	7.090188	6.084827	Down_tDCS
**Slc25a2**	-0.0504	9.61E-01	NA	1.963142	2.939825	2.9594296	3.04009	2.015586	2.025768	3.042414	Down_tDCS
**Ttc19**	-0.0485	8.28E-01	NA	44.1707	44.09737	48.33735	45.60135	40.31172	45.57978	44.62207	Down_tDCS
**Adck5**	-0.0467	8.80E-01	NA	24.53928	25.47848	23.675437	24.32072	24.18703	24.30922	22.31103	Down_tDCS
**Adck2**	-0.0463	8.88E-01	NA	21.59456	21.55871	22.68896	22.29399	20.15586	21.27056	21.29689	Down_tDCS
**Slc25a26**	-0.0454	9.00E-01	NA	17.66828	17.63895	18.743054	17.22718	16.12469	19.2448	17.24034	Down_tDCS
**Nbr1**	-0.0444	8.15E-01	NA	60.85741	57.81655	62.148022	56.74834	60.46759	58.74727	57.80586	Down_tDCS
**Zadh2**	-0.043	9.20E-01	NA	11.77885	14.69912	11.837718	12.16036	12.09352	12.15461	13.18379	Down_tDCS
**Crls1**	-0.0424	8.95E-01	NA	21.59456	23.5186	23.675437	21.28063	21.16366	23.29633	23.32517	Down_tDCS
**Fam136a**	-0.042	8.99E-01	NA	19.63142	21.55871	23.675437	19.2539	21.16366	22.28345	21.29689	Down_tDCS
**Nfs1**	-0.0418	8.55E-01	NA	44.1707	45.07731	40.445538	42.56126	42.32731	41.52825	41.57965	Down_tDCS
**Polb**	-0.0418	8.77E-01	NA	31.41028	31.35813	32.553726	30.4009	31.24159	31.3994	30.42414	Down_tDCS
**Atad3a**	-0.0417	8.78E-01	NA	29.44713	33.31801	32.553726	31.41426	31.24159	31.3994	29.41	Down_tDCS
**Hibch**	-0.0414	9.03E-01	NA	20.61299	18.61889	21.702484	19.2539	19.14807	21.27056	19.26862	Down_tDCS
**Akr1b10**	-0.0407	8.93E-01	NA	21.59456	27.43836	34.526679	28.37417	25.19483	27.34787	27.38172	Down_tDCS
**Fastkd1**	-0.04	9.39E-01	NA	7.852569	9.799416	8.8782888	9.120269	9.070138	9.115956	7.098965	Down_tDCS
**Ptcd1**	-0.0399	9.13E-01	NA	17.66828	18.61889	16.770101	16.21381	18.14028	17.21903	17.24034	Down_tDCS
**Snap29**	-0.0393	8.97E-01	NA	26.50242	25.47848	23.675437	24.32072	24.18703	24.30922	25.35345	Down_tDCS
**Mrps5**	-0.039	8.83E-01	NA	32.39185	33.31801	32.553726	31.41426	30.23379	33.42517	32.45241	Down_tDCS
**Pdk3**	-0.0381	9.06E-01	NA	21.59456	22.53866	23.675437	22.29399	21.16366	21.27056	23.32517	Down_tDCS
**Pex11b**	-0.0381	8.52E-01	NA	53.00484	53.89679	51.29678	50.66816	50.38966	52.66997	51.72103	Down_tDCS
**Hsdl1**	-0.037	8.99E-01	NA	26.50242	27.43836	28.60782	28.37417	27.21041	26.33498	25.35345	Down_tDCS
**Sfxn3**	-0.0364	8.48E-01	NA	58.89427	61.73632	59.188592	60.80179	57.44421	58.74727	56.79172	Down_tDCS
**Rmnd1**	-0.0364	9.03E-01	NA	24.53928	25.47848	28.60782	26.34744	25.19483	25.3221	25.35345	Down_tDCS
**Slc25a15**	-0.0354	9.35E-01	NA	11.77885	12.73924	12.824195	12.16036	11.08572	12.15461	13.18379	Down_tDCS
**Bckdk**	-0.0353	8.77E-01	NA	43.18913	47.0372	40.445538	43.57462	39.30393	45.57978	41.57965	Down_tDCS
**Aldh9a1**	-0.0349	8.80E-01	NA	43.18913	45.07731	38.472585	40.53453	43.3351	39.50248	41.57965	Down_tDCS
**Elac2**	-0.0348	9.01E-01	NA	29.44713	30.37819	29.594296	28.37417	29.226	30.38652	28.39586	Down_tDCS
**Spire1**	-0.0344	9.26E-01	NA	15.70514	18.61889	17.756578	17.22718	17.13248	17.21903	16.22621	Down_tDCS
**Apoo**	-0.0343	9.26E-01	NA	17.66828	17.63895	16.770101	16.21381	16.12469	18.23191	17.24034	Down_tDCS
**Spr**	-0.0339	8.88E-01	NA	40.24442	42.13749	36.499632	35.46771	39.30393	41.52825	38.53724	Down_tDCS
**Slc25a38**	-0.0327	8.96E-01	NA	37.2997	40.1776	33.540202	33.44099	37.28835	36.46383	37.5231	Down_tDCS
**Pus1**	-0.0327	9.22E-01	NA	20.61299	21.55871	20.716007	20.26726	21.16366	20.25768	20.28276	Down_tDCS
**Mcat**	-0.0327	9.22E-01	NA	21.59456	20.57877	20.716007	21.28063	20.15586	20.25768	20.28276	Down_tDCS
**Yrdc**	-0.0323	9.06E-01	NA	30.42871	30.37819	31.567249	29.38753	29.226	31.3994	30.42414	Down_tDCS
**Lactb2**	-0.0322	9.07E-01	NA	30.42871	31.35813	30.580773	30.4009	27.21041	31.3994	31.43827	Down_tDCS
**Coq3**	-0.0321	8.99E-01	NA	35.33656	37.23778	34.526679	33.44099	33.25717	36.46383	36.50896	Down_tDCS
**Pck2**	-0.0315	9.11E-01	NA	28.46556	30.37819	29.594296	27.36081	29.226	31.3994	27.38172	Down_tDCS
**Acp6**	-0.0314	9.28E-01	NA	20.61299	19.59883	18.743054	19.2539	18.14028	19.2448	20.28276	Down_tDCS
**Gtpbp10**	-0.0313	9.49E-01	NA	9.815711	10.77936	8.8782888	10.13363	9.070138	10.12884	9.127241	Down_tDCS
**Nnt**	-0.0305	9.23E-01	NA	22.57614	22.53866	24.661913	22.29399	22.17145	23.29633	23.32517	Down_tDCS
**Plscr3**	-0.0303	9.24E-01	NA	23.55771	25.47848	20.716007	22.29399	23.17924	23.29633	22.31103	Down_tDCS
**Ndufaf4**	-0.0301	9.33E-01	NA	18.64985	17.63895	18.743054	19.2539	17.13248	18.23191	17.24034	Down_tDCS
**Aldh3a2**	-0.0296	9.00E-01	NA	39.26285	40.1776	41.432014	40.53453	37.28835	39.50248	40.56551	Down_tDCS
**Tomm70a**	-0.0294	8.74E-01	NA	64.78369	57.81655	74.972217	66.88197	63.49097	65.83746	61.86241	Down_tDCS
**Amacr**	-0.0272	9.31E-01	NA	25.52085	24.49854	22.68896	24.32072	22.17145	22.28345	26.36758	Down_tDCS
**Abhd10**	-0.0263	9.40E-01	NA	17.66828	20.57877	19.729531	21.28063	18.14028	18.23191	18.25448	Down_tDCS
**Ogdhl**	-0.0263	9.14E-01	NA	38.28127	40.1776	37.486108	42.56126	37.28835	36.46383	35.49482	Down_tDCS
**Tdrkh**	-0.0256	9.22E-01	NA	32.39185	33.31801	35.513155	32.42762	32.24938	34.43806	33.46655	Down_tDCS
**Tmem70**	-0.0255	9.14E-01	NA	41.22599	39.19766	42.418491	42.56126	38.29614	41.52825	38.53724	Down_tDCS
**Ndufa10**	-0.025	9.34E-01	NA	33.37342	22.53866	30.580773	26.34744	32.24938	31.3994	23.32517	Down_tDCS
**Suox**	-0.0246	9.40E-01	NA	20.61299	22.53866	21.702484	20.26726	22.17145	21.27056	21.29689	Down_tDCS
**Nsun2**	-0.0246	9.40E-01	NA	21.59456	21.55871	21.702484	21.28063	21.16366	21.27056	21.29689	Down_tDCS
**Mlycd**	-0.0246	9.18E-01	NA	39.26285	40.1776	39.459061	40.53453	39.30393	37.47671	38.53724	Down_tDCS
**Tmem205**	-0.0246	9.31E-01	NA	28.46556	30.37819	27.621343	27.36081	29.226	29.37364	27.38172	Down_tDCS
**Dars2**	-0.0246	9.51E-01	NA	13.742	14.69912	14.797148	14.18709	13.10131	15.19326	14.19793	Down_tDCS
**Chpt1**	-0.0245	9.22E-01	NA	37.2997	37.23778	33.540202	34.45435	36.28055	34.43806	36.50896	Down_tDCS
**Txnrd2**	-0.0245	9.41E-01	NA	22.57614	22.53866	19.729531	21.28063	22.17145	19.2448	22.31103	Down_tDCS
**Comtd1**	-0.0245	9.46E-01	NA	18.64985	18.61889	16.770101	19.2539	17.13248	16.20614	18.25448	Down_tDCS
**Casp9**	-0.0238	9.14E-01	NA	46.13384	48.01714	42.418491	45.60135	45.35069	44.5669	43.60793	Down_tDCS
**Pycr2**	-0.0235	9.27E-01	NA	36.31813	36.25784	31.567249	34.45435	33.25717	34.43806	34.48069	Down_tDCS
**Taz**	-0.0224	9.36E-01	NA	28.46556	29.39825	31.567249	29.38753	29.226	31.3994	27.38172	Down_tDCS
**Trmt5**	-0.0221	9.58E-01	NA	12.76042	12.73924	13.810671	12.16036	12.09352	14.18038	13.18379	Down_tDCS
**Trnt1**	-0.022	9.41E-01	NA	26.50242	25.47848	26.634866	26.34744	24.18703	27.34787	25.35345	Down_tDCS
**Rhot2**	-0.0204	9.17E-01	NA	54.96798	55.85667	56.229162	56.74834	55.42862	52.66997	54.76344	Down_tDCS
**Rtn4ip1**	-0.0199	9.60E-01	NA	17.66828	14.69912	13.810671	15.20045	12.09352	16.20614	17.24034	Down_tDCS

**Aldh6a1**	-0.0193	9.36E-01	NA	40.24442	41.15755	35.513155	36.48108	39.30393	38.48959	39.55138	Down_tDCS
**Pnpt1**	-0.019	9.66E-01	NA	11.77885	10.77936	12.824195	11.147	11.08572	13.16749	11.15552	Down_tDCS
**Mthfd2**	-0.0188	9.77E-01	NA	6.870998	4.899708	5.9188592	6.080179	5.038966	6.077304	6.084827	Down_tDCS
**Cpt2**	-0.0173	9.66E-01	NA	13.742	15.67907	12.824195	12.16036	14.1091	15.19326	14.19793	Down_tDCS
**Qrsl1**	-0.0165	9.65E-01	NA	15.70514	15.67907	17.756578	16.21381	16.12469	16.20614	16.22621	Down_tDCS
**Ethe1**	-0.0163	9.45E-01	NA	40.24442	45.07731	37.486108	40.53453	42.32731	40.51536	38.53724	Down_tDCS
**Armcx3**	-0.0156	9.26E-01	NA	76.56255	70.55579	76.94517	72.96215	72.5611	75.9663	74.03206	Down_tDCS
**Ldhd**	-0.0155	9.65E-01	NA	20.61299	18.61889	16.770101	19.2539	19.14807	18.23191	17.24034	Down_tDCS
**Slc25a46**	-0.0152	9.42E-01	NA	49.07856	48.99708	52.283256	49.6548	47.36628	49.63132	51.72103	Down_tDCS
**Slc25a29**	-0.0143	9.64E-01	NA	25.52085	24.49854	19.729531	23.30735	24.18703	21.27056	23.32517	Down_tDCS
**Gatc**	-0.0142	9.47E-01	NA	50.06013	49.97702	46.364397	45.60135	47.36628	48.61843	51.72103	Down_tDCS
**Cisd3**	-0.0141	9.61E-01	NA	35.33656	34.29795	31.567249	34.45435	21.16366	38.48959	39.55138	Down_tDCS
**Aifm1**	-0.0141	9.63E-01	NA	24.53928	26.45842	25.64839	24.32072	25.19483	26.33498	25.35345	Down_tDCS
**Abcd3**	-0.0123	9.56E-01	NA	43.18913	45.07731	43.404968	42.56126	43.3351	44.5669	43.60793	Down_tDCS
**Gtpbp3**	-0.0103	9.84E-01	NA	8.83414	8.819474	9.8647653	8.106906	11.08572	8.103072	9.127241	Down_tDCS
**Tk2**	-0.0102	9.74E-01	NA	25.52085	21.55871	28.60782	25.33408	25.19483	23.29633	26.36758	Down_tDCS
**Gfer**	-0.0099	9.78E-01	NA	18.64985	18.61889	17.756578	20.26726	18.14028	17.21903	17.24034	Down_tDCS
**Lactb**	-0.0098	9.83E-01	NA	10.79728	12.73924	10.851242	11.147	10.07793	13.16749	11.15552	Down_tDCS
**Fundc1**	-0.0074	9.82E-01	NA	23.55771	21.55871	19.729531	22.29399	21.16366	21.27056	21.29689	Down_tDCS
**Nudt19**	-0.0064	9.82E-01	NA	30.42871	29.39825	28.60782	28.37417	29.226	29.37364	30.42414	Down_tDCS
**Dhx30**	-0.0063	9.74E-01	NA	56.93113	55.85667	57.215639	57.76171	56.43641	56.72151	54.76344	Down_tDCS
**Angel2**	-0.0058	9.85E-01	NA	25.52085	23.5186	25.64839	25.33408	25.19483	24.30922	24.33931	Down_tDCS
**Ptrh2**	-0.0046	9.92E-01	NA	9.815711	10.77936	9.8647653	10.13363	9.070138	10.12884	11.15552	Down_tDCS
**Mccc1**	-0.0043	9.91E-01	NA	18.64985	17.63895	17.756578	17.22718	20.15586	17.21903	17.24034	Down_tDCS
**Abcb8**	-0.0039	9.86E-01	NA	44.1707	46.05725	41.432014	43.57462	44.3429	42.54113	44.62207	Down_tDCS
**L2hgdh**	-0.0035	9.95E-01	NA	7.852569	6.859591	8.8782888	7.093543	8.062345	8.103072	8.113103	Down_tDCS
**Cyp11b2**	-0.0033	9.95E-01	NA	6.870998	8.819474	7.8918123	9.120269	8.062345	7.090188	7.098965	Down_tDCS
**Hmgcl**	-0.0033	9.92E-01	NA	22.57614	25.47848	22.68896	23.30735	24.18703	24.30922	22.31103	Down_tDCS
**Bckdha**	-0.0026	9.89E-01	NA	60.85741	62.71626	51.29678	60.80179	58.452	56.72151	56.79172	Down_tDCS
**Dhrs1**	-0.0025	9.94E-01	NA	22.57614	21.55871	19.729531	21.28063	22.17145	18.23191	23.32517	Down_tDCS
**Tfb1m**	-0.0025	9.94E-01	NA	21.59456	21.55871	20.716007	22.29399	20.15586	22.28345	20.28276	Down_tDCS
**Bckdhb**	-0.0025	9.91E-01	NA	43.18913	44.09737	40.445538	44.58798	40.31172	43.55401	41.57965	Down_tDCS
**Exog**	-0.0021	9.96E-01	NA	11.77885	14.69912	13.810671	12.16036	13.10131	14.18038	14.19793	Down_tDCS
**Nipsnap3b**	-0.0021	9.94E-01	NA	23.55771	29.39825	27.621343	28.37417	25.19483	27.34787	26.36758	Down_tDCS
**Ccdc58**	-0.0006	9.99E-01	NA	10.79728	11.7593	10.851242	10.13363	12.09352	11.14172	11.15552	Down_tDCS
**Mrpl16**	-0.0006	9.98E-01	NA	33.37342	33.31801	33.540202	32.42762	34.26497	33.42517	33.46655	Down_tDCS
**Sfxn1**	-0.0006	9.98E-01	NA	44.1707	44.09737	45.377921	45.60135	44.3429	45.57978	42.59379	Down_tDCS
**Golph3**	-0.0005	9.98E-01	NA	40.24442	38.21772	38.472585	39.52117	37.28835	39.50248	39.55138	Down_tDCS
**Abat**	0.00016	9.99E-01	NA	82.45198	78.39533	85.823459	84.10915	82.63904	77.99207	84.17344	Up_tDCS
**Mrpl44**	0.00058	9.98E-01	NA	37.2997	43.11743	39.459061	37.49444	39.30393	40.51536	42.59379	Up_tDCS
**Dhrs7b**	0.0008	9.97E-01	NA	48.09699	49.97702	48.33735	48.64144	47.36628	49.63132	49.69275	Up_tDCS
**Mrpl14**	0.00145	9.95E-01	NA	46.13384	50.95696	42.418491	49.6548	44.3429	47.60555	44.62207	Up_tDCS
**Mmaa**	0.00166	9.97E-01	NA	8.83414	7.839533	9.8647653	8.106906	9.070138	9.115956	9.127241	Up_tDCS
**Bak1**	0.00204	9.96E-01	NA	18.64985	18.61889	15.783625	17.22718	17.13248	18.23191	18.25448	Up_tDCS
**Acsf2**	0.00239	9.92E-01	NA	40.24442	42.13749	33.540202	39.52117	39.30393	37.47671	38.53724	Up_tDCS
**Hadha**	0.00251	9.90E-01	NA	53.00484	52.91684	56.229162	51.68153	57.44421	52.66997	54.76344	Up_tDCS
**Pmpca**	0.00265	9.89E-01	NA	56.93113	52.91684	52.283256	56.74834	53.41304	52.66997	53.74931	Up_tDCS
**Agpat5**	0.00267	9.92E-01	NA	34.35499	31.35813	33.540202	33.44099	32.24938	33.42517	33.46655	Up_tDCS
**Mrpl36**	0.00366	9.87E-01	NA	43.18913	49.97702	45.377921	45.60135	46.35848	47.60555	45.6362	Up_tDCS
**Coq2**	0.00413	9.83E-01	NA	56.93113	56.83661	54.256209	54.72162	52.40524	58.74727	58.82	Up_tDCS
**Ptcd3**	0.00461	9.87E-01	NA	28.46556	26.45842	30.580773	26.34744	28.21821	29.37364	30.42414	Up_tDCS
**Slc25a20**	0.00489	9.86E-01	NA	28.46556	30.37819	26.634866	28.37417	26.20262	29.37364	30.42414	Up_tDCS
**Pdk4**	0.00542	9.95E-01	NA	3.926285	2.939825	2.9594296	3.04009	5.038966	3.038652	2.028276	Up_tDCS
**Mrpl47**	0.00588	9.88E-01	NA	16.68671	15.67907	16.770101	16.21381	17.13248	17.21903	15.21207	Up_tDCS
**Acly**	0.00695	9.67E-01	NA	76.56255	71.53573	73.98574	74.98888	74.57669	74.95342	73.01792	Up_tDCS
**Pccb**	0.00732	9.79E-01	NA	30.42871	32.33807	28.60782	32.42762	29.226	29.37364	31.43827	Up_tDCS
**Acss1**	0.00831	9.75E-01	NA	34.35499	40.1776	29.594296	33.44099	35.27276	33.42517	37.5231	Up_tDCS
**Gsr**	0.00923	9.72E-01	NA	31.41028	31.35813	34.526679	33.44099	33.25717	33.42517	30.42414	Up_tDCS
**Mrps9**	0.00961	9.67E-01	NA	38.28127	41.15755	40.445538	39.52117	40.31172	41.52825	39.55138	Up_tDCS
**Mrps2**	0.00963	9.67E-01	NA	40.24442	40.1776	39.459061	41.54789	40.31172	38.48959	40.56551	Up_tDCS
**Abcb10**	0.01002	9.78E-01	NA	18.64985	19.59883	16.770101	17.22718	19.14807	19.2448	18.25448	Up_tDCS
**Mrpl2**	0.01049	9.61E-01	NA	46.13384	51.9369	47.350874	47.62807	50.38966	49.63132	47.66448	Up_tDCS
**Rfk**	0.01055	9.64E-01	NA	41.22599	42.13749	39.459061	44.58798	37.28835	41.52825	41.57965	Up_tDCS
**Tmlhe**	0.011	9.84E-01	NA	7.852569	7.839533	6.9053357	8.106906	7.054552	7.090188	8.113103	Up_tDCS
**Tmem186**	0.0114	9.69E-01	NA	26.50242	27.43836	26.634866	25.33408	27.21041	27.34787	28.39586	Up_tDCS
**Echdc2**	0.01232	9.78E-01	NA	11.77885	12.73924	10.851242	12.16036	11.08572	12.15461	12.16965	Up_tDCS
**Crat**	0.01257	9.42E-01	NA	69.69155	75.4555	70.039834	73.97552	73.5689	73.94053	67.94724	Up_tDCS
**Htra2**	0.01267	9.55E-01	NA	43.18913	47.0372	41.432014	45.60135	44.3429	44.5669	42.59379	Up_tDCS
**Tomm40**	0.01267	9.35E-01	NA	91.28612	90.15462	81.877552	86.13588	88.68579	88.12091	91.27241	Up_tDCS
**Dnajc11**	0.0128	9.52E-01	NA	48.09699	49.97702	46.364397	49.6548	49.38186	46.59267	48.67862	Up_tDCS

**Hint2**	0.01329	9.41E-01	NA	65.76527	68.59591	61.161545	65.86861	65.50655	67.86323	63.89068	Up_tDCS
**Fam185a**	0.01449	9.62E-01	NA	24.53928	26.45842	25.64839	23.30735	26.20262	26.33498	27.38172	Up_tDCS
**Tmem126a**	0.01464	9.78E-01	NA	8.83414	8.819474	7.8918123	9.120269	8.062345	9.115956	8.113103	Up_tDCS
**Pgam5**	0.01531	9.52E-01	NA	34.35499	36.25784	34.526679	36.48108	35.27276	36.46383	33.46655	Up_tDCS
**Immt**	0.01544	9.39E-01	NA	50.06013	51.9369	57.215639	53.70825	54.42083	52.66997	53.74931	Up_tDCS
**Slc25a1**	0.01644	9.41E-01	NA	49.07856	47.0372	40.445538	46.61471	47.36628	43.55401	46.65034	Up_tDCS
**Yars2**	0.01659	9.68E-01	NA	14.72357	12.73924	13.810671	13.17372	12.09352	16.20614	14.19793	Up_tDCS
**Cpt1c**	0.01788	9.32E-01	NA	49.07856	47.0372	49.323827	49.6548	50.38966	47.60555	48.67862	Up_tDCS
**Nln**	0.0179	9.54E-01	NA	22.57614	23.5186	26.634866	24.32072	22.17145	25.3221	26.36758	Up_tDCS
**Ppox**	0.01846	9.63E-01	NA	16.68671	14.69912	12.824195	15.20045	14.1091	15.19326	15.21207	Up_tDCS
**Myg1**	0.01862	9.25E-01	NA	54.96798	55.85667	53.269733	55.73498	53.41304	56.72151	55.77758	Up_tDCS
**Slc25a19**	0.01977	9.47E-01	NA	25.52085	27.43836	25.64839	26.34744	28.21821	25.3221	26.36758	Up_tDCS
**Timm44**	0.01986	9.31E-01	NA	41.22599	43.11743	41.432014	42.56126	40.31172	43.55401	43.60793	Up_tDCS
**Rhot1**	0.02035	9.33E-01	NA	36.31813	37.23778	39.459061	37.49444	37.28835	39.50248	38.53724	Up_tDCS
**Acad9**	0.02079	9.26E-01	NA	43.18913	43.11743	45.377921	44.58798	43.3351	45.57978	44.62207	Up_tDCS
**Idi1**	0.02107	9.55E-01	NA	17.66828	14.69912	17.756578	19.2539	16.12469	15.19326	17.24034	Up_tDCS
**Atpaf1**	0.02114	9.35E-01	NA	32.39185	33.31801	34.526679	35.46771	32.24938	33.42517	34.48069	Up_tDCS
**Lias**	0.02115	9.35E-01	NA	33.37342	34.29795	32.553726	33.44099	34.26497	35.45094	32.45241	Up_tDCS
**Spg7**	0.02123	9.36E-01	NA	32.39185	37.23778	30.580773	35.46771	35.27276	33.42517	31.43827	Up_tDCS
**Pick1**	0.02264	9.22E-01	NA	42.20756	44.09737	38.472585	43.57462	40.31172	41.52825	43.60793	Up_tDCS
**Agk**	0.02281	9.34E-01	NA	28.46556	31.35813	30.580773	31.41426	29.226	31.3994	30.42414	Up_tDCS
**Tomm34**	0.02283	8.81E-01	NA	91.28612	91.13457	88.782888	94.24278	87.678	95.2111	90.25827	Up_tDCS
**Ptges2**	0.02297	9.19E-01	NA	42.20756	44.09737	41.432014	42.56126	44.3429	43.55401	42.59379	Up_tDCS
**Lyrm1**	0.02338	9.70E-01	NA	5.889427	6.859591	5.9188592	6.080179	7.054552	6.077304	6.084827	Up_tDCS
**Fdx1**	0.02342	9.47E-01	NA	19.63142	17.63895	18.743054	17.22718	18.14028	20.25768	20.28276	Up_tDCS
**Echdc1**	0.02345	9.31E-01	NA	30.42871	32.33807	30.580773	31.41426	31.24159	31.3994	32.45241	Up_tDCS
**Mpv17l**	0.02352	9.40E-01	NA	22.57614	29.39825	22.68896	23.30735	26.20262	28.36075	23.32517	Up_tDCS
**Afg3l2**	0.02382	9.14E-01	NA	44.1707	44.09737	45.377921	44.58798	44.3429	45.57978	46.65034	Up_tDCS
**Etfb**	0.02509	8.85E-01	NA	74.59941	79.37527	69.053357	79.04233	68.52993	85.08226	69.97551	Up_tDCS
**Timm22**	0.02802	9.04E-01	NA	41.22599	42.13749	39.459061	39.52117	43.3351	43.55401	40.56551	Up_tDCS
**Ak3**	0.02812	8.93E-01	NA	51.0417	49.97702	46.364397	50.66816	47.36628	50.6442	51.72103	Up_tDCS
**Sfxn4**	0.02896	9.37E-01	NA	17.66828	17.63895	16.770101	18.24054	16.12469	18.23191	18.25448	Up_tDCS
**Mmadhc**	0.02934	8.94E-01	NA	44.1707	47.0372	43.404968	46.61471	45.35069	45.57978	45.6362	Up_tDCS
**Dld**	0.02954	8.62E-01	NA	74.59941	68.59591	76.94517	79.04233	71.55331	70.90188	78.08861	Up_tDCS
**Mcee**	0.02977	9.05E-01	NA	36.31813	40.1776	33.540202	36.48108	35.27276	37.47671	40.56551	Up_tDCS
**Mpst**	0.02979	9.05E-01	NA	37.2997	40.1776	32.553726	37.49444	35.27276	38.48959	38.53724	Up_tDCS
**Mrpl10**	0.02988	8.89E-01	NA	47.11541	47.0372	46.364397	46.61471	46.35848	49.63132	48.67862	Up_tDCS
**Timm9**	0.02998	9.17E-01	NA	26.50242	29.39825	29.594296	24.32072	29.226	32.41229	30.42414	Up_tDCS
**Mrps31**	0.03001	9.16E-01	NA	26.50242	29.39825	29.594296	28.37417	28.21821	30.38652	29.41	Up_tDCS
**Gatm**	0.03124	8.48E-01	NA	91.28612	86.23486	72.012787	86.13588	85.66241	89.13379	79.10275	Up_tDCS
**Acot9**	0.03188	8.86E-01	NA	42.20756	45.07731	46.364397	44.58798	42.32731	48.61843	46.65034	Up_tDCS
**Dnaja3**	0.03239	8.65E-01	NA	60.85741	56.83661	58.202116	55.73498	60.46759	60.77304	62.87655	Up_tDCS
**Nudt5**	0.03271	9.40E-01	NA	10.79728	12.73924	12.824195	11.147	12.09352	13.16749	13.18379	Up_tDCS
**Oxr1**	0.03279	8.26E-01	NA	98.15711	91.13457	104.56651	106.4031	92.71697	101.2884	100.3996	Up_tDCS
**Mtx2**	0.0328	8.95E-01	NA	36.31813	37.23778	35.513155	37.49444	35.27276	39.50248	36.50896	Up_tDCS
**Hsd17b4**	0.03393	8.83E-01	NA	41.22599	42.13749	40.445538	39.52117	42.32731	42.54113	44.62207	Up_tDCS
**Me2**	0.03401	9.34E-01	NA	16.68671	11.7593	13.810671	12.16036	15.1169	15.19326	15.21207	Up_tDCS
**mrpl11**	0.0341	8.60E-01	NA	53.00484	58.79649	57.215639	58.77507	57.44421	57.73439	56.79172	Up_tDCS
**Maoa**	0.03427	8.78E-01	NA	43.18913	42.13749	44.391444	43.57462	43.3351	44.5669	45.6362	Up_tDCS
**Trap1**	0.03432	8.58E-01	NA	58.89427	58.79649	54.256209	60.80179	57.44421	59.76016	56.79172	Up_tDCS
**Mfn2**	0.03461	8.35E-01	NA	74.59941	74.47556	76.94517	78.02897	77.60007	76.97919	76.06034	Up_tDCS
**Ptcd2**	0.03477	9.29E-01	NA	15.70514	15.67907	13.810671	16.21381	15.1169	15.19326	15.21207	Up_tDCS
**Surf1**	0.03503	8.28E-01	NA	79.50726	79.37527	78.918123	80.0557	81.63124	83.05649	80.11689	Up_tDCS
**Cox18**	0.0354	9.26E-01	NA	16.68671	17.63895	13.810671	14.18709	15.1169	19.2448	17.24034	Up_tDCS
**Eri3**	0.03608	8.31E-01	NA	70.67312	76.43544	72.012787	79.04233	72.5611	73.94053	74.03206	Up_tDCS
**Gldc**	0.0362	9.21E-01	NA	20.61299	19.59883	13.810671	18.24054	19.14807	19.2448	17.24034	Up_tDCS
**Bnip3l**	0.03682	8.41E-01	NA	62.82055	61.73632	61.161545	65.86861	60.46759	63.81169	63.89068	Up_tDCS
**Sdhaf2**	0.03752	8.61E-01	NA	47.11541	47.0372	46.364397	47.62807	45.35069	48.61843	50.70689	Up_tDCS
**Mrpl4**	0.03858	8.39E-01	NA	59.87584	60.75638	55.242686	63.84188	57.44421	59.76016	59.83413	Up_tDCS
**Fahd2a**	0.03868	8.36E-01	NA	57.9127	63.6962	60.175069	61.81516	58.452	64.82458	63.89068	Up_tDCS
**Pgs1**	0.03879	8.32E-01	NA	60.85741	65.65608	61.161545	64.85525	62.48317	64.82458	64.90482	Up_tDCS
**Tfam**	0.03882	8.84E-01	NA	30.42871	33.31801	31.567249	31.41426	32.24938	34.43806	32.45241	Up_tDCS
**Bola1**	0.03908	8.82E-01	NA	32.39185	36.25784	29.594296	34.45435	32.24938	34.43806	33.46655	Up_tDCS
**Hadh**	0.03941	8.73E-01	NA	37.2997	39.19766	33.540202	38.5078	37.28835	37.47671	37.5231	Up_tDCS
**Ung**	0.04206	9.72E-01	NA	1.963142	1.959883	1.9729531	1.013363	3.023379	1.012884	3.042414	Up_tDCS
**Bco2**	0.04251	9.79E-01	NA	0.981571	0.979942	0.9864765	1.013363	1.007793	1.012884	1.014138	Up_tDCS
**Gpat2**	0.04251	9.79E-01	NA	0.981571	0.979942	0.9864765	1.013363	1.007793	1.012884	1.014138	Up_tDCS
**Recql4**	0.04251	9.79E-01	NA	0.981571	0.979942	0.9864765	1.013363	1.007793	1.012884	1.014138	Up_tDCS
**Bid**	0.04252	9.17E-01	NA	13.742	13.71918	13.810671	12.16036	14.1091	14.18038	16.22621	Up_tDCS
**Ecsit**	0.04255	8.24E-01	NA	56.93113	58.79649	55.242686	58.77507	58.452	58.74727	58.82	Up_tDCS

**Timm17b**	0.0426	8.86E-01	NA	28.46556	27.43836	23.675437	30.4009	28.21821	27.34787	23.32517	Up_tDCS
**Diablo**	0.04263	8.64E-01	NA	35.33656	39.19766	34.526679	35.46771	36.28055	38.48959	39.55138	Up_tDCS
**Eci1**	0.04266	8.68E-01	NA	36.31813	36.25784	30.580773	33.44099	35.27276	35.45094	37.5231	Up_tDCS
**Mrpl23**	0.04488	8.27E-01	NA	49.07856	53.89679	49.323827	49.6548	52.40524	55.70862	51.72103	Up_tDCS
**Cyb5r3**	0.0449	8.25E-01	NA	52.02327	54.87673	48.33735	54.72162	52.40524	52.66997	53.74931	Up_tDCS
**Mrps30**	0.04536	8.43E-01	NA	43.18913	41.15755	41.432014	43.57462	41.31952	46.59267	41.57965	Up_tDCS
**Pdhx**	0.04559	8.50E-01	NA	36.31813	38.21772	39.459061	39.52117	38.29614	39.50248	39.55138	Up_tDCS
**Ogdh**	0.04606	7.53E-01	NA	96.19397	96.03427	102.59356	104.3764	101.7871	98.24975	101.4138	Up_tDCS
**Armc10**	0.04614	8.59E-01	NA	32.39185	35.2779	31.567249	33.44099	34.26497	35.45094	33.46655	Up_tDCS
**Mettl5**	0.04655	8.66E-01	NA	28.46556	32.33807	29.594296	32.42762	28.21821	33.42517	30.42414	Up_tDCS
**Prepl**	0.04681	7.47E-01	NA	104.0465	97.01421	119.36366	109.4432	104.8105	111.4172	115.6117	Up_tDCS
**Coq10a**	0.04722	8.17E-01	NA	52.02327	53.89679	48.33735	51.68153	51.39745	51.65709	57.80586	Up_tDCS
**Gls**	0.04725	7.47E-01	NA	98.15711	95.05433	103.58004	105.3898	97.75593	105.3399	100.3996	Up_tDCS
**Supv3l1**	0.04737	8.77E-01	NA	23.55771	25.47848	23.675437	24.32072	26.20262	25.3221	24.33931	Up_tDCS
**Bok**	0.04772	8.79E-01	NA	23.55771	24.49854	21.702484	24.32072	23.17924	23.29633	25.35345	Up_tDCS
**Fxn**	0.04773	8.26E-01	NA	46.13384	51.9369	41.432014	47.62807	48.37407	48.61843	47.66448	Up_tDCS
**Oxa1l**	0.04832	7.62E-01	NA	81.4704	87.2148	77.931646	83.09579	84.65462	87.10803	85.18758	Up_tDCS
**Mrpl55**	0.04895	8.45E-01	NA	42.20756	32.33807	35.513155	40.53453	36.28055	39.50248	35.49482	Up_tDCS
**Ndufaf3**	0.04929	8.45E-01	NA	34.35499	34.29795	35.513155	36.48108	35.27276	35.45094	36.50896	Up_tDCS
**Mtrf1l**	0.04965	8.96E-01	NA	18.64985	15.67907	14.797148	14.18709	19.14807	19.2448	15.21207	Up_tDCS
**Bcl2l2**	0.05049	7.62E-01	NA	74.59941	75.4555	72.012787	76.00224	77.60007	76.97919	76.06034	Up_tDCS
**Efhd1**	0.05061	8.21E-01	NA	47.11541	45.07731	40.445538	44.58798	46.35848	42.54113	49.69275	Up_tDCS
**Mrpl37**	0.05085	7.92E-01	NA	54.96798	58.79649	56.229162	57.76171	58.452	57.73439	60.84827	Up_tDCS
**Mto1**	0.05204	8.64E-01	NA	23.55771	26.45842	24.661913	23.30735	25.19483	26.33498	28.39586	Up_tDCS
**Aadat**	0.05267	9.07E-01	NA	9.815711	12.73924	11.837718	11.147	13.10131	12.15461	11.15552	Up_tDCS
**Mrps18b**	0.05316	7.85E-01	NA	53.98641	56.83661	55.242686	57.76171	56.43641	58.74727	56.79172	Up_tDCS
**Coq9**	0.05337	7.86E-01	NA	53.98641	56.83661	52.283256	56.74834	57.44421	54.69574	56.79172	Up_tDCS
**Oxnad1**	0.05369	9.08E-01	NA	9.815711	10.77936	10.851242	10.13363	11.08572	11.14172	11.15552	Up_tDCS
**Triap1**	0.05384	8.43E-01	NA	30.42871	34.29795	29.594296	28.37417	32.24938	36.46383	33.46655	Up_tDCS
**Chchd6**	0.05419	7.72E-01	NA	61.83898	63.6962	57.215639	68.9087	61.47538	63.81169	58.82	Up_tDCS
**Slc25a14**	0.05483	8.20E-01	NA	36.31813	38.21772	39.459061	36.48108	40.31172	41.52825	39.55138	Up_tDCS
**Fundc2**	0.055	8.46E-01	NA	31.41028	27.43836	26.634866	29.38753	28.21821	30.38652	30.42414	Up_tDCS
**Pmpcb**	0.05527	7.58E-01	NA	61.83898	65.65608	66.093928	66.88197	65.50655	67.86323	67.94724	Up_tDCS
**Cpt1a**	0.05561	8.76E-01	NA	17.66828	19.59883	16.770101	19.2539	20.15586	17.21903	18.25448	Up_tDCS
**Acat1**	0.05698	7.50E-01	NA	66.74684	65.65608	63.134498	70.93543	67.52214	64.82458	67.94724	Up_tDCS
**Mrpl19**	0.05815	8.32E-01	NA	28.46556	32.33807	29.594296	30.4009	31.24159	32.41229	31.43827	Up_tDCS
**Rdh14**	0.05886	8.56E-01	NA	21.59456	21.55871	21.702484	23.30735	21.16366	23.29633	22.31103	Up_tDCS
**Coq5**	0.05901	8.12E-01	NA	34.35499	37.23778	35.513155	37.49444	36.28055	36.46383	38.53724	Up_tDCS
**Mrpl15**	0.05929	7.30E-01	NA	72.63626	71.53573	67.080404	73.97552	68.52993	76.97919	74.03206	Up_tDCS
**Mrps11**	0.05974	7.78E-01	NA	47.11541	50.95696	45.377921	47.62807	51.39745	51.65709	48.67862	Up_tDCS
**Acads**	0.05993	8.60E-01	NA	23.55771	22.53866	15.783625	20.26726	21.16366	21.27056	23.32517	Up_tDCS
**Oat**	0.05995	7.32E-01	NA	66.74684	69.57585	66.093928	69.92206	69.53772	72.92765	68.96137	Up_tDCS
**Mrpl51**	0.0605	8.37E-01	NA	26.50242	26.45842	25.64839	27.36081	26.20262	27.34787	28.39586	Up_tDCS
**Bola3**	0.06108	8.18E-01	NA	30.42871	34.29795	30.580773	32.42762	32.24938	34.43806	33.46655	Up_tDCS
**Mrpl18**	0.06116	7.83E-01	NA	43.18913	44.09737	44.391444	42.56126	48.37407	47.60555	44.62207	Up_tDCS
**Dguok**	0.06196	8.40E-01	NA	24.53928	24.49854	23.675437	27.36081	24.18703	23.29633	26.36758	Up_tDCS
**Mrpl35**	0.06198	8.01E-01	NA	36.31813	37.23778	35.513155	38.5078	35.27276	38.48959	39.55138	Up_tDCS
**Hspa9**	0.06211	6.85E-01	NA	86.37826	86.23486	95.688224	91.20269	92.71697	95.2111	94.31482	Up_tDCS
**Slc25a33**	0.06361	7.73E-01	NA	45.15227	47.0372	41.432014	47.62807	49.38186	44.5669	44.62207	Up_tDCS
**Cox19**	0.06374	8.06E-01	NA	32.39185	36.25784	31.567249	34.45435	32.24938	36.46383	36.50896	Up_tDCS
**Atad1**	0.06389	7.30E-01	NA	59.87584	58.79649	62.148022	62.82852	61.47538	64.82458	62.87655	Up_tDCS
**Nipsnap1**	0.06436	6.87E-01	NA	77.54412	83.29503	80.891076	83.09579	82.63904	86.09514	85.18758	Up_tDCS
**Acadvl**	0.06536	7.61E-01	NA	47.11541	47.0372	44.391444	47.62807	50.38966	47.60555	47.66448	Up_tDCS
**Timm8a1**	0.06589	8.47E-01	NA	20.61299	23.5186	16.770101	20.26726	20.15586	25.3221	19.26862	Up_tDCS
**Hibadh**	0.06721	7.38E-01	NA	53.98641	53.89679	49.323827	54.72162	52.40524	55.70862	56.79172	Up_tDCS
**Hagh**	0.06751	6.75E-01	NA	79.50726	82.31509	77.931646	87.14924	78.60786	85.08226	84.17344	Up_tDCS
**Serac1**	0.06777	8.98E-01	NA	6.870998	11.7593	8.8782888	6.080179	13.10131	11.14172	8.113103	Up_tDCS
**Nif3l1**	0.068	8.68E-01	NA	14.72357	13.71918	12.824195	12.16036	16.12469	15.19326	14.19793	Up_tDCS
**Mrps23**	0.06809	8.12E-01	NA	26.50242	29.39825	26.634866	27.36081	29.226	30.38652	28.39586	Up_tDCS
**Kars**	0.0681	7.05E-01	NA	61.83898	67.61597	63.134498	65.86861	65.50655	70.90188	66.9331	Up_tDCS
**Abcd1**	0.06815	8.92E-01	NA	8.83414	9.799416	8.8782888	10.13363	9.070138	10.12884	9.127241	Up_tDCS
**Sdr39u1**	0.06858	7.41E-01	NA	47.11541	51.9369	49.323827	54.72162	50.38966	52.66997	49.69275	Up_tDCS
**mrpl9**	0.06871	7.54E-01	NA	43.18913	48.99708	42.418491	45.60135	46.35848	47.60555	48.67862	Up_tDCS
**Dnajc4**	0.06873	7.68E-01	NA	40.24442	43.11743	37.486108	43.57462	43.3351	41.52825	40.56551	Up_tDCS
**Mrps35**	0.0688	7.68E-01	NA	42.20756	41.15755	37.486108	43.57462	39.30393	44.5669	41.57965	Up_tDCS
**Nfu1**	0.06888	7.97E-01	NA	31.41028	31.35813	30.580773	32.42762	32.24938	32.41229	33.46655	Up_tDCS
**Fastk**	0.0689	6.82E-01	NA	78.52569	79.37527	70.039834	83.09579	70.54552	83.05649	82.14517	Up_tDCS
**Nudt9**	0.07004	6.97E-01	NA	63.80212	66.63603	61.161545	68.9087	67.52214	64.82458	66.9331	Up_tDCS
**Lyrm2**	0.07038	8.30E-01	NA	19.63142	21.55871	21.702484	21.28063	22.17145	22.28345	22.31103	Up_tDCS
**Prdx3**	0.07071	6.88E-01	NA	65.76527	68.59591	65.107451	68.9087	68.52993	69.889	72.00379	Up_tDCS

**Apex1**	0.07115	6.88E-01	NA	65.76527	67.61597	63.134498	70.93543	66.51435	68.87611	68.96137	Up_tDCS
**Sptlc2**	0.07136	8.39E-01	NA	22.57614	13.71918	23.675437	20.26726	22.17145	20.25768	21.29689	Up_tDCS
**Bad**	0.07141	6.83E-01	NA	68.70998	74.47556	64.120975	69.92206	69.53772	77.99207	73.01792	Up_tDCS
**Ndufaf2**	0.07159	8.02E-01	NA	27.48399	30.37819	26.634866	27.36081	31.24159	32.41229	27.38172	Up_tDCS
**Ivd**	0.07189	7.01E-01	NA	62.82055	62.71626	54.256209	60.80179	62.48317	64.82458	63.89068	Up_tDCS
**Coq10b**	0.07234	8.16E-01	NA	24.53928	22.53866	23.675437	25.33408	22.17145	25.3221	26.36758	Up_tDCS
**Dlst**	0.07252	6.55E-01	NA	77.54412	78.39533	77.931646	79.04233	80.62345	85.08226	83.1593	Up_tDCS
**Fmc1**	0.07339	8.32E-01	NA	19.63142	19.59883	17.756578	20.26726	19.14807	21.27056	19.26862	Up_tDCS
**Acaa2**	0.07493	8.20E-01	NA	18.64985	24.49854	21.702484	24.32072	23.17924	24.30922	19.26862	Up_tDCS
**Etfdh**	0.075	8.02E-01	NA	24.53928	26.45842	24.661913	25.33408	26.20262	26.33498	28.39586	Up_tDCS
**Coq7**	0.07509	7.62E-01	NA	36.31813	39.19766	32.553726	37.49444	37.28835	38.48959	38.53724	Up_tDCS
**Nudt2**	0.07722	6.87E-01	NA	57.9127	59.77644	54.256209	56.74834	59.45979	60.77304	64.90482	Up_tDCS
**Slc25a22**	0.07752	5.57E-01	6.02E-01	122.6964	126.4125	121.33661	136.804	126.9819	131.6749	125.7531	Up_tDCS
**Pdk2**	0.07761	5.97E-01	NA	97.17554	100.934	91.742318	102.3497	101.7871	99.26263	104.4562	Up_tDCS
**Mavs**	0.07998	8.51E-01	NA	12.76042	12.73924	11.837718	12.16036	14.1091	13.16749	13.18379	Up_tDCS
**Aurkaip1**	0.08082	6.27E-01	NA	75.58098	84.27497	69.053357	83.09579	81.63124	80.01784	78.08861	Up_tDCS
**Rab24**	0.08139	6.39E-01	NA	69.69155	73.49562	64.120975	68.9087	73.5689	74.95342	75.0462	Up_tDCS
**Pdss2**	0.08158	7.91E-01	NA	23.55771	25.47848	22.68896	24.32072	25.19483	27.34787	24.33931	Up_tDCS
**Vdac1**	0.08164	7.91E-01	NA	24.53928	25.47848	21.702484	25.33408	25.19483	24.30922	26.36758	Up_tDCS
**Mrpl28**	0.08298	6.47E-01	NA	62.82055	68.59591	59.188592	67.89534	64.49876	67.86323	68.96137	Up_tDCS
**Sod2**	0.08346	5.86E-01	NA	91.28612	83.29503	89.769365	92.21606	91.70917	96.22398	93.30068	Up_tDCS
**Bdh1**	0.08347	7.31E-01	NA	37.2997	38.21772	35.513155	40.53453	39.30393	38.48959	38.53724	Up_tDCS
**mrpl24**	0.08437	7.42E-01	NA	33.37342	34.29795	32.553726	34.45435	35.27276	35.45094	36.50896	Up_tDCS
**Chchd4**	0.08494	7.92E-01	NA	21.59456	21.55871	22.68896	26.34744	22.17145	22.28345	22.31103	Up_tDCS
**Mrps25**	0.08584	6.06E-01	NA	73.61784	81.33515	71.026311	74.98888	80.62345	83.05649	81.13103	Up_tDCS
**Gars**	0.0864	5.91E-01	NA	77.54412	80.35521	80.891076	84.10915	79.61566	87.10803	87.21585	Up_tDCS
**Idh3a**	0.08693	5.42E-01	NA	104.0465	100.934	101.60708	108.4299	105.8183	109.3915	110.541	Up_tDCS
**C1qbp**	0.08751	5.96E-01	NA	75.58098	77.41538	72.012787	80.0557	80.62345	79.00495	79.10275	Up_tDCS
**Mrps24**	0.0879	6.83E-01	NA	47.11541	45.07731	46.364397	50.66816	45.35069	47.60555	52.73517	Up_tDCS
**Prdx4**	0.08839	7.44E-01	NA	30.42871	31.35813	29.594296	30.4009	31.24159	33.42517	34.48069	Up_tDCS
**Gtpbp6**	0.08885	6.74E-01	NA	48.09699	51.9369	43.404968	51.68153	49.38186	52.66997	49.69275	Up_tDCS
**Paics**	0.08934	6.56E-01	NA	50.06013	52.91684	53.269733	54.72162	55.42862	58.74727	52.73517	Up_tDCS
**Slc25a25**	0.08997	7.56E-01	NA	24.53928	27.43836	28.60782	27.36081	30.23379	29.37364	27.38172	Up_tDCS
**Ptpmt1**	0.09008	6.92E-01	NA	40.24442	44.09737	40.445538	44.58798	42.32731	46.59267	43.60793	Up_tDCS
**Mrpl32**	0.09185	7.56E-01	NA	24.53928	26.45842	26.634866	26.34744	27.21041	30.38652	26.36758	Up_tDCS
**Mrpl53**	0.09299	6.05E-01	NA	64.78369	69.57585	59.188592	69.92206	68.52993	70.90188	65.91896	Up_tDCS
**Prodh**	0.09322	6.11E-01	NA	63.80212	65.65608	56.229162	63.84188	68.52993	64.82458	66.9331	Up_tDCS
**Cyp27a1**	0.09326	8.75E-01	NA	5.889427	6.859591	7.8918123	9.120269	5.038966	8.103072	7.098965	Up_tDCS
**Mrps28**	0.09391	7.56E-01	NA	25.52085	26.45842	22.68896	27.36081	26.20262	28.36075	24.33931	Up_tDCS
**Msrb2**	0.09394	6.74E-01	NA	41.22599	45.07731	42.418491	43.57462	45.35069	46.59267	47.66448	Up_tDCS
**Mrpl38**	0.09413	6.32E-01	NA	53.00484	57.81655	51.29678	59.78843	56.43641	57.73439	56.79172	Up_tDCS
**Mrps12**	0.09468	6.64E-01	NA	45.15227	46.05725	42.418491	47.62807	46.35848	48.61843	47.66448	Up_tDCS
**Mrps15**	0.09619	7.12E-01	NA	32.39185	33.31801	31.567249	32.42762	34.26497	36.46383	35.49482	Up_tDCS
**Timm10**	0.09625	7.13E-01	NA	31.41028	35.2779	30.580773	35.46771	34.26497	35.45094	33.46655	Up_tDCS
**Cs**	0.09631	4.52E-01	5.13E-01	128.5858	127.3924	138.10671	140.8575	137.0599	142.8166	140.9652	Up_tDCS
**Sirt5**	0.09647	5.04E-01	NA	99.13868	100.934	96.6747	103.3631	103.8027	108.3786	107.4986	Up_tDCS
**Slc25a11**	0.09649	4.88E-01	5.42E-01	109.936	105.8337	106.53947	118.5635	111.865	115.4688	113.5834	Up_tDCS
**Mrpl49**	0.09651	5.94E-01	NA	60.85741	61.73632	64.120975	66.88197	65.50655	68.87611	64.90482	Up_tDCS
**Samm50**	0.09861	5.75E-01	NA	64.78369	68.59591	65.107451	69.92206	67.52214	73.94053	72.00379	Up_tDCS
**Mrps18c**	0.0991	7.42E-01	NA	23.55771	25.47848	24.661913	26.34744	26.20262	26.33498	26.36758	Up_tDCS
**Fh1**	0.0999	5.51E-01	NA	69.69155	76.43544	72.012787	78.02897	75.58448	76.97919	81.13103	Up_tDCS
**Decr1**	0.10048	7.07E-01	NA	29.44713	37.23778	29.594296	33.44099	31.24159	34.43806	38.53724	Up_tDCS
**Eci2**	0.10056	6.76E-01	NA	38.28127	40.1776	35.513155	37.49444	38.29614	42.54113	44.62207	Up_tDCS
**Dut**	0.10093	6.93E-01	NA	35.33656	34.29795	31.567249	36.48108	35.27276	38.48959	34.48069	Up_tDCS
**Mrps10**	0.10159	7.57E-01	NA	16.68671	27.43836	31.567249	28.37417	33.25717	22.28345	24.33931	Up_tDCS
**Slc30a9**	0.10211	5.33E-01	NA	76.56255	72.51568	77.931646	79.04233	80.62345	83.05649	82.14517	Up_tDCS
**Mrps16**	0.10343	6.17E-01	NA	49.07856	52.91684	46.364397	50.66816	51.39745	56.72151	53.74931	Up_tDCS
**Aldh7a1**	0.10367	6.07E-01	NA	52.02327	51.9369	49.323827	52.69489	54.42083	56.72151	55.77758	Up_tDCS
**Mrps14**	0.10438	6.56E-01	NA	39.26285	41.15755	37.486108	41.54789	40.31172	45.57978	41.57965	Up_tDCS
**Mrpl45**	0.10449	6.92E-01	NA	32.39185	33.31801	29.594296	32.42762	33.25717	35.45094	35.49482	Up_tDCS
**Ndufs1**	0.10523	4.89E-01	NA	86.37826	87.2148	91.742318	94.24278	91.70917	98.24975	96.3431	Up_tDCS
**Rpia**	0.10556	8.32E-01	NA	8.83414	9.799416	8.8782888	9.120269	10.07793	10.12884	10.14138	Up_tDCS
**Nit1**	0.10603	5.77E-01	NA	60.85741	59.77644	54.256209	56.74834	61.47538	66.85035	65.91896	Up_tDCS
**Mrps17**	0.10749	7.20E-01	NA	24.53928	26.45842	23.675437	25.33408	28.21821	27.34787	26.36758	Up_tDCS
**Gcdh**	0.10751	5.99E-01	NA	50.06013	51.9369	47.350874	54.72162	54.42083	53.68285	51.72103	Up_tDCS
**Agpat4**	0.10832	5.23E-01	NA	77.54412	73.49562	65.107451	74.98888	77.60007	77.99207	80.11689	Up_tDCS
**Htatip2**	0.10886	7.62E-01	NA	15.70514	16.65901	19.729531	18.24054	18.14028	19.2448	19.26862	Up_tDCS
**Acot13**	0.10913	6.64E-01	NA	35.33656	35.2779	33.540202	37.49444	34.26497	38.48959	39.55138	Up_tDCS
**Idh2**	0.10933	5.72E-01	NA	55.94955	58.79649	51.29678	59.78843	59.45979	60.77304	58.82	Up_tDCS
**Tmem126b**	0.10943	6.46E-01	NA	37.2997	39.19766	37.486108	40.53453	38.29614	42.54113	42.59379	Up_tDCS

**Higd1a**	0.10956	4.96E-01	NA	81.4704	80.35521	75.958693	83.09579	83.64683	84.06937	91.27241	Up_tDCS
**Pxmp2**	0.10961	7.52E-01	NA	17.66828	22.53866	16.770101	20.26726	19.14807	21.27056	21.29689	Up_tDCS
**Sucla2**	0.10962	4.43E-01	NA	101.1018	101.9139	101.60708	109.4432	103.8027	112.4301	112.5693	Up_tDCS
**Sdhaf1**	0.1097	5.46E-01	NA	61.83898	62.71626	61.161545	66.88197	62.48317	68.87611	68.96137	Up_tDCS
**Acsf3**	0.10978	7.40E-01	NA	20.61299	22.53866	18.743054	21.28063	22.17145	21.27056	24.33931	Up_tDCS
**Gcsh**	0.11072	5.64E-01	NA	57.9127	61.73632	53.269733	57.76171	58.452	64.82458	67.94724	Up_tDCS
**Acadl**	0.11116	6.47E-01	NA	36.31813	40.1776	34.526679	37.49444	41.31952	38.48959	42.59379	Up_tDCS
**Bax**	0.11176	5.72E-01	NA	54.96798	56.83661	48.33735	57.76171	57.44421	57.73439	57.80586	Up_tDCS
**Ahcyl1**	0.11209	4.59E-01	NA	87.35983	87.2148	93.715271	94.24278	95.74035	99.26263	97.35723	Up_tDCS
**Gls2**	0.11287	6.09E-01	NA	42.20756	44.09737	46.364397	43.57462	47.36628	46.59267	53.74931	Up_tDCS
**Immp1l**	0.11291	6.36E-01	NA	38.28127	37.23778	37.486108	39.52117	40.31172	40.51536	42.59379	Up_tDCS
**Mrpl22**	0.11292	6.73E-01	NA	33.37342	30.37819	29.594296	30.4009	34.26497	36.46383	33.46655	Up_tDCS
**Tomm22**	0.11296	4.88E-01	NA	76.56255	80.35521	73.98574	81.06906	81.63124	88.12091	82.14517	Up_tDCS
**Dcakd**	0.11309	6.48E-01	NA	36.31813	40.1776	31.567249	40.53453	38.29614	38.48959	38.53724	Up_tDCS
**Mrps7**	0.11401	5.23E-01	NA	65.76527	67.61597	60.175069	65.86861	71.55331	70.90188	70.98965	Up_tDCS
**Ssbp1**	0.11486	6.46E-01	NA	35.33656	36.25784	33.540202	35.46771	38.29614	41.52825	36.50896	Up_tDCS
**Phb2**	0.11598	4.43E-01	NA	89.32297	93.09445	85.823459	96.26951	95.74035	99.26263	96.3431	Up_tDCS
**Mrpl41**	0.1161	5.15E-01	NA	61.83898	66.63603	64.120975	67.89534	66.51435	73.94053	69.97551	Up_tDCS
**Dnajc15**	0.11618	6.03E-01	NA	42.20756	44.09737	40.445538	46.61471	44.3429	45.57978	46.65034	Up_tDCS
**Mtif2**	0.11713	7.58E-01	NA	14.72357	14.69912	16.770101	16.21381	17.13248	17.21903	16.22621	Up_tDCS
**Mrpl46**	0.11785	6.04E-01	NA	41.22599	43.11743	39.459061	41.54789	45.35069	44.5669	47.66448	Up_tDCS
**Sdha**	0.11797	3.51E-01	4.20E-01	130.549	133.2721	134.16081	145.9243	142.0988	145.8553	141.9793	Up_tDCS
**Nrd1**	0.11807	5.72E-01	NA	46.13384	45.07731	59.188592	53.70825	52.40524	55.70862	55.77758	Up_tDCS
**Mtch2**	0.11866	4.52E-01	NA	79.50726	80.35521	84.836982	87.14924	85.66241	92.17245	89.24413	Up_tDCS
**Phyh**	0.1188	4.55E-01	NA	82.45198	86.23486	75.958693	86.13588	85.66241	89.13379	93.30068	Up_tDCS
**Dap3**	0.11935	6.52E-01	NA	31.41028	31.35813	31.567249	31.41426	33.25717	35.45094	36.50896	Up_tDCS
**Rexo2**	0.12009	5.32E-01	NA	54.96798	56.83661	57.215639	56.74834	58.452	66.85035	62.87655	Up_tDCS
**Ghitm**	0.1202	3.26E-01	4.01E-01	140.3647	144.0514	154.87682	157.0713	156.2079	161.0486	162.2621	Up_tDCS
**Chchd3**	0.12081	5.46E-01	NA	52.02327	51.9369	50.310303	55.73498	54.42083	54.69574	58.82	Up_tDCS
**Ndufa9**	0.12116	4.02E-01	NA	96.19397	99.95404	97.661177	105.3898	106.8261	104.3271	109.5269	Up_tDCS
**Comt**	0.12129	5.60E-01	NA	48.09699	49.97702	46.364397	50.66816	55.42862	50.6442	52.73517	Up_tDCS
**Grpel1**	0.12153	5.01E-01	NA	58.89427	65.65608	63.134498	67.89534	66.51435	69.889	67.94724	Up_tDCS
**Slc25a39**	0.12157	5.32E-01	NA	56.93113	59.77644	49.323827	57.76171	59.45979	60.77304	62.87655	Up_tDCS
**Suclg2**	0.12169	5.71E-01	NA	44.1707	51.9369	43.404968	47.62807	51.39745	48.61843	54.76344	Up_tDCS
**Prkaca**	0.12295	4.04E-01	NA	94.23083	97.01421	91.742318	103.3631	98.76373	104.3271	104.4562	Up_tDCS
**Hmgcs2**	0.12299	8.36E-01	NA	8.83414	8.819474	3.9459061	6.080179	7.054552	10.12884	8.113103	Up_tDCS
**Ech1**	0.12384	4.99E-01	NA	63.80212	67.61597	55.242686	67.89534	69.53772	63.81169	69.97551	Up_tDCS
**Scp2**	0.12422	4.59E-01	NA	71.65469	74.47556	69.053357	77.01561	76.59228	77.99207	81.13103	Up_tDCS
**Slc25a18**	0.12434	4.51E-01	NA	75.58098	84.27497	72.012787	80.0557	82.63904	82.04361	92.28654	Up_tDCS
**Mrps33**	0.12442	4.91E-01	NA	62.82055	70.55579	60.175069	65.86861	72.5611	76.97919	65.91896	Up_tDCS
**Ndufv3**	0.12508	4.58E-01	NA	68.70998	79.37527	69.053357	80.0557	76.59228	79.00495	80.11689	Up_tDCS
**Auh**	0.12624	4.24E-01	NA	81.4704	83.29503	77.931646	88.1626	87.678	89.13379	88.22999	Up_tDCS
**Casp3**	0.12637	7.42E-01	NA	15.70514	14.69912	14.797148	16.21381	15.1169	17.21903	17.24034	Up_tDCS
**Etfa**	0.12665	5.38E-01	NA	51.0417	49.97702	46.364397	51.68153	52.40524	56.72151	53.74931	Up_tDCS
**Mrpl12**	0.12742	5.29E-01	NA	52.02327	56.83661	45.377921	54.72162	56.43641	56.72151	56.79172	Up_tDCS
**Nme3**	0.1277	4.91E-01	NA	59.87584	62.71626	55.242686	63.84188	65.50655	65.83746	63.89068	Up_tDCS
**Gstk1**	0.12833	6.33E-01	NA	29.44713	34.29795	28.60782	31.41426	32.24938	35.45094	35.49482	Up_tDCS
**Mrpl3**	0.12842	5.18E-01	NA	51.0417	53.89679	51.29678	54.72162	55.42862	58.74727	58.82	Up_tDCS
**Mecr**	0.12871	6.92E-01	NA	21.59456	22.53866	19.729531	21.28063	25.19483	21.27056	25.35345	Up_tDCS
**Mrpl1**	0.13022	6.76E-01	NA	22.57614	25.47848	22.68896	25.33408	21.16366	29.37364	27.38172	Up_tDCS
**Mgst1**	0.13022	8.09E-01	NA	7.852569	8.819474	6.9053357	8.106906	8.062345	9.115956	9.127241	Up_tDCS
**Msra**	0.13077	5.84E-01	NA	37.2997	39.19766	36.499632	42.56126	41.31952	37.47671	43.60793	Up_tDCS
**Acadm**	0.13096	6.30E-01	NA	30.42871	31.35813	27.621343	30.4009	34.26497	32.41229	33.46655	Up_tDCS
**Mrps34**	0.13242	4.78E-01	NA	57.9127	62.71626	55.242686	63.84188	64.49876	63.81169	64.90482	Up_tDCS
**Mrpl48**	0.13302	4.84E-01	NA	57.9127	59.77644	53.269733	60.80179	59.45979	64.82458	64.90482	Up_tDCS
**Mrpl52**	0.13528	5.16E-01	NA	47.11541	52.91684	44.391444	52.69489	52.40524	53.68285	52.73517	Up_tDCS
**Pdha1**	0.13549	3.41E-01	NA	103.065	98.9741	100.62061	112.4833	107.8339	112.4301	110.541	Up_tDCS
**Lap3**	0.13555	5.05E-01	NA	53.00484	50.95696	47.350874	51.68153	55.42862	54.69574	59.83413	Up_tDCS
**Mrpl21**	0.13691	5.73E-01	NA	35.33656	38.21772	35.513155	39.52117	38.29614	42.54113	39.55138	Up_tDCS
**Mpv17l2**	0.1377	5.33E-01	NA	42.20756	46.05725	41.432014	48.64144	49.38186	44.5669	47.66448	Up_tDCS
**Txn2**	0.13832	3.65E-01	NA	88.3414	91.13457	81.877552	94.24278	95.74035	95.2111	98.37137	Up_tDCS
**Glrx5**	0.14068	5.94E-01	NA	31.41028	34.29795	28.60782	33.44099	34.26497	35.45094	35.49482	Up_tDCS
**Pld6**	0.14231	8.99E-01	NA	2.944713	2.939825	0.9864765	1.013363	4.031172	2.025768	3.042414	Up_tDCS
**Lonp1**	0.14541	4.44E-01	NA	54.96798	64.67614	53.269733	60.80179	65.50655	64.82458	63.89068	Up_tDCS
**Sirt3**	0.14604	4.25E-01	NA	59.87584	64.67614	57.215639	66.88197	65.50655	66.85035	68.96137	Up_tDCS
**Ppa2**	0.14659	5.13E-01	NA	40.24442	43.11743	41.432014	44.58798	46.35848	47.60555	45.6362	Up_tDCS
**Isca1**	0.1468	2.83E-01	3.59E-01	108.9544	114.6532	107.52594	121.6036	119.9274	121.5461	125.7531	Up_tDCS
**Cmpk2**	0.14685	6.71E-01	NA	19.63142	18.61889	20.716007	19.2539	19.14807	28.36075	20.28276	Up_tDCS
**Abcb6**	0.14689	5.26E-01	NA	39.26285	41.15755	37.486108	42.56126	44.3429	44.5669	42.59379	Up_tDCS
**Endog**	0.14807	5.73E-01	NA	34.35499	35.2779	27.621343	34.45435	36.28055	33.42517	39.55138	Up_tDCS

**Mrpl34**	0.14826	4.15E-01	NA	64.78369	66.63603	56.229162	69.92206	64.49876	73.94053	68.96137	Up_tDCS
**Oxct1**	0.14826	3.19E-01	NA	93.24926	97.99416	86.809935	102.3497	98.76373	106.3528	103.4421	Up_tDCS
**Tmem14c**	0.14889	3.61E-01	NA	71.65469	80.35521	75.958693	81.06906	83.64683	86.09514	86.20172	Up_tDCS
**Idh3B**	0.14921	2.59E-01	3.38E-01	117.7885	124.4526	116.40423	130.7239	128.9975	135.7265	134.8803	Up_tDCS
**Echs1**	0.14927	3.94E-01	NA	68.70998	68.59591	61.161545	71.94879	72.5611	71.91477	77.07448	Up_tDCS
**Oma1**	0.15079	8.23E-01	NA	4.907856	4.899708	5.9188592	6.080179	6.046759	6.077304	5.070689	Up_tDCS
**Ndufv1**	0.1508	2.29E-01	3.10E-01	136.4384	141.1116	129.22843	153.0179	149.1534	151.9326	148.0641	Up_tDCS
**Timm17a**	0.15084	3.74E-01	NA	72.63626	73.49562	65.107451	76.00224	76.59228	82.04361	78.08861	Up_tDCS
**Hscb**	0.15109	6.41E-01	NA	20.61299	21.55871	20.716007	22.29399	22.17145	25.3221	23.32517	Up_tDCS
**Hspd1**	0.15343	3.15E-01	NA	86.37826	86.23486	88.782888	97.28287	89.69359	100.2755	100.3996	Up_tDCS
**Hadhb**	0.15418	3.77E-01	NA	68.70998	66.63603	63.134498	68.9087	73.5689	75.9663	76.06034	Up_tDCS
**Mrps18a**	0.15454	3.21E-01	NA	80.48883	87.2148	81.877552	91.20269	89.69359	96.22398	93.30068	Up_tDCS
**Sdhd**	0.15461	2.92E-01	NA	94.23083	98.9741	89.769365	106.4031	101.7871	105.3399	106.4845	Up_tDCS
**Dhrs4**	0.15481	7.28E-01	NA	11.77885	11.7593	9.8647653	12.16036	13.10131	12.15461	12.16965	Up_tDCS
**Higd2a**	0.15498	2.66E-01	3.43E-01	106.0097	109.7535	102.59356	117.5501	112.8728	123.5719	118.6541	Up_tDCS
**Bnip3**	0.15509	3.09E-01	NA	90.30454	84.27497	85.823459	99.3096	92.71697	98.24975	96.3431	Up_tDCS
**Idh3g**	0.15558	2.39E-01	3.20E-01	118.7701	122.4927	114.43128	133.7639	132.0209	132.6878	129.8096	Up_tDCS
**Slc25a28**	0.1556	4.93E-01	NA	44.1707	47.0372	35.513155	49.6548	47.36628	47.60555	43.60793	Up_tDCS
**Ndufs4**	0.15623	2.71E-01	NA	96.19397	107.7936	104.56651	113.4967	111.865	119.5203	113.5834	Up_tDCS
**Ndufs8**	0.157	2.71E-01	NA	101.1018	107.7936	94.701747	114.51	111.865	113.443	111.5552	Up_tDCS
**Polg2**	0.15803	8.64E-01	NA	2.944713	2.939825	2.9594296	3.04009	3.023379	4.051536	3.042414	Up_tDCS
**Fkbp10**	0.15808	8.01E-01	NA	5.889427	5.879649	5.9188592	7.093543	6.046759	6.077304	7.098965	Up_tDCS
**Cycs**	0.15854	2.92E-01	NA	103.065	94.07439	87.796412	114.51	95.74035	106.3528	107.4986	Up_tDCS
**Got2**	0.15908	1.99E-01	2.83E-01	136.4384	141.1116	137.12024	155.0446	149.1534	156.997	156.1772	Up_tDCS
**Ndufab1**	0.15958	5.95E-01	NA	24.53928	24.49854	23.675437	27.36081	25.19483	28.36075	27.38172	Up_tDCS
**Tomm20**	0.15962	2.34E-01	3.15E-01	115.8254	117.593	112.45833	133.7639	123.9586	131.6749	125.7531	Up_tDCS
**Bloc1s1**	0.16029	3.09E-01	NA	86.37826	88.19474	73.98574	92.21606	93.72476	94.19821	90.25827	Up_tDCS
**Mrpl13**	0.16146	3.65E-01	NA	61.83898	65.65608	61.161545	67.89534	72.5611	70.90188	69.97551	Up_tDCS
**Mrps26**	0.16169	3.80E-01	NA	56.93113	65.65608	57.215639	63.84188	67.52214	68.87611	67.94724	Up_tDCS
**Uqcrfs1**	0.1622	2.29E-01	3.10E-01	113.8623	119.5529	110.48537	129.7105	121.943	130.662	130.8238	Up_tDCS
**Pde2a**	0.16455	2.29E-01	3.10E-01	105.0281	115.6331	110.48537	124.6437	125.9741	123.5719	120.6824	Up_tDCS
**Pdhb**	0.16689	3.10E-01	NA	71.65469	76.43544	72.999264	83.09579	79.61566	85.08226	83.1593	Up_tDCS
**Timm23**	0.16711	2.60E-01	NA	90.30454	94.07439	90.755841	99.3096	100.7793	105.3399	106.4845	Up_tDCS
**Pcca**	0.1681	6.66E-01	NA	14.72357	14.69912	13.810671	15.20045	16.12469	16.20614	17.24034	Up_tDCS
**Arl2**	0.16937	2.46E-01	NA	100.1203	99.95404	88.782888	112.4833	106.8261	105.3399	108.5127	Up_tDCS
**Ndufa12**	0.17226	2.15E-01	2.97E-01	106.0097	111.7133	103.58004	118.5635	114.8884	126.6105	122.7107	Up_tDCS
**Pxmp4**	0.17444	6.07E-01	NA	21.59456	17.63895	24.661913	21.28063	30.23379	18.23191	26.36758	Up_tDCS
**Tst**	0.17485	3.11E-01	NA	73.61784	81.33515	60.175069	78.02897	79.61566	83.05649	83.1593	Up_tDCS
**Gadd45gip1**	0.17631	2.68E-01	NA	80.48883	83.29503	73.98574	88.1626	87.678	93.18533	89.24413	Up_tDCS
**Fdps**	0.17681	2.20E-01	NA	97.17554	100.934	96.6747	115.5234	103.8027	113.443	111.5552	Up_tDCS
**Nit2**	0.17682	4.78E-01	NA	38.28127	30.37819	39.459061	40.53453	41.31952	45.57978	35.49482	Up_tDCS
**Ndufa7**	0.17817	2.00E-01	2.83E-01	116.807	117.593	99.63413	129.7105	131.0131	123.5719	119.6683	Up_tDCS
**Ociad1**	0.17963	1.40E-01	2.26E-01	142.3278	149.9311	144.02557	162.1381	157.2157	172.1903	167.3327	Up_tDCS
**Cisd1**	0.18009	1.83E-01	2.68E-01	111.8991	118.5729	108.51242	125.657	124.9663	132.6878	128.7955	Up_tDCS
**Mff**	0.18035	2.04E-01	2.87E-01	107.9728	107.7936	95.688224	113.4967	112.8728	123.5719	120.6824	Up_tDCS
**Suclg1**	0.18292	1.75E-01	2.61E-01	110.9175	117.593	110.48537	125.657	124.9663	130.662	131.8379	Up_tDCS
**Sdhb**	0.18326	1.52E-01	2.37E-01	133.4937	135.2319	122.32309	153.0179	142.0988	145.8553	151.1065	Up_tDCS
**Fam162a**	0.18335	2.22E-01	NA	91.28612	97.99416	88.782888	97.28287	100.7793	114.4559	108.5127	Up_tDCS
**Uqcrc2**	0.18405	1.65E-01	2.52E-01	116.807	117.593	118.37718	129.7105	128.9975	137.7522	137.9227	Up_tDCS
**Ndufs7**	0.1852	1.41E-01	2.27E-01	154.1067	151.8909	129.22843	173.2851	161.2469	168.1387	157.1914	Up_tDCS
**Atp5c1**	0.18713	1.03E-01	1.88E-01	162.9408	174.4296	170.66044	185.4455	186.4417	200.551	198.771	Up_tDCS
**Pink1**	0.18744	1.25E-01	2.12E-01	152.1435	149.9311	135.14729	166.1916	168.3015	160.0357	169.361	Up_tDCS
**Glud1**	0.18848	1.49E-01	2.33E-01	125.6411	130.3322	120.35014	147.951	138.0677	134.7136	151.1065	Up_tDCS
**Maob**	0.18856	3.76E-01	NA	45.15227	51.9369	41.432014	51.68153	50.38966	52.66997	55.77758	Up_tDCS
**Uqcrc1**	0.19124	1.09E-01	1.95E-01	150.1804	152.8709	145.01205	169.2317	168.3015	171.1774	173.4176	Up_tDCS
**Ckmt1**	0.19157	8.77E-02	1.67E-01	171.7749	177.3694	174.60635	198.6192	192.4885	205.6155	200.7993	Up_tDCS
**Aco2**	0.19254	9.68E-02	1.80E-01	159.0145	161.6904	156.84977	180.3787	180.395	182.3191	184.5731	Up_tDCS
**Arf5**	0.19303	9.89E-02	1.82E-01	159.9961	173.4497	153.89034	188.4856	179.3872	188.3964	186.6014	Up_tDCS
**Ndufb3**	0.19341	1.92E-01	NA	89.32297	96.03427	88.782888	100.323	102.7949	107.3657	107.4986	Up_tDCS
**Mrpl54**	0.19371	2.36E-01	NA	73.61784	83.29503	70.039834	89.17597	83.64683	87.10803	86.20172	Up_tDCS
**Sdhc**	0.19462	1.48E-01	2.32E-01	113.8623	122.4927	108.51242	130.7239	127.9897	131.6749	135.8945	Up_tDCS
**Prelid1**	0.19774	1.22E-01	2.08E-01	128.5858	135.2319	121.33661	147.951	143.1066	148.894	149.0783	Up_tDCS
**Stoml2**	0.19812	3.10E-01	NA	50.06013	63.6962	50.310303	61.81516	61.47538	63.81169	63.89068	Up_tDCS
**Ndufa5**	0.19832	1.66E-01	NA	96.19397	103.8738	101.60708	119.5769	107.8339	122.559	111.5552	Up_tDCS
**Mccc2**	0.20111	4.75E-01	NA	28.46556	30.37819	23.675437	32.42762	32.24938	30.38652	31.43827	Up_tDCS
**Grsf1**	0.20152	2.82E-01	NA	62.82055	53.89679	56.229162	64.85525	62.48317	66.85035	70.98965	Up_tDCS
**Slc25a5**	0.20198	9.02E-02	1.70E-01	147.2357	152.8709	151.91739	174.2985	165.2781	178.2676	175.4458	Up_tDCS
**Mrpl30**	0.20244	4.37E-01	NA	30.42871	33.31801	30.580773	34.45435	35.27276	39.50248	35.49482	Up_tDCS
**Ndufs3**	0.20266	1.61E-01	NA	94.23083	101.9139	92.728794	109.4432	107.8339	114.4559	111.5552	Up_tDCS
**Chchd10**	0.20372	6.02E-02	1.35E-01	214.9641	231.2662	192.36292	248.274	236.8314	251.1952	244.4072	Up_tDCS

**Mrpl20**	0.20568	2.25E-01	NA	66.74684	74.47556	66.093928	83.09579	76.59228	81.03072	78.08861	Up_tDCS
**Ndufa2**	0.20575	1.60E-01	NA	92.26769	97.01421	91.742318	104.3764	104.8105	114.4559	108.5127	Up_tDCS
**Romo1**	0.20731	1.75E-01	NA	90.30454	97.99416	79.904599	109.4432	99.77152	105.3399	98.37137	Up_tDCS
**Ndufa3**	0.20891	1.08E-01	1.94E-01	126.6227	143.0715	118.37718	148.9644	149.1534	153.9584	146.0358	Up_tDCS
**Timm13**	0.20924	1.52E-01	NA	95.2124	102.8939	88.782888	108.4299	108.8417	115.4688	109.5269	Up_tDCS
**Prodh2**	0.21059	9.21E-01	NA	0.981571	0	0.9864765	1.013363	1.007793	1.012884	0	Up_tDCS
**Cpt1b**	0.21205	8.52E-01	NA	1.963142	1.959883	1.9729531	2.026726	3.023379	2.025768	2.028276	Up_tDCS
**Mrps36**	0.21238	5.09E-01	NA	17.66828	23.5186	23.675437	23.30735	24.18703	28.36075	24.33931	Up_tDCS
**Nubpl**	0.2126	6.54E-01	NA	11.77885	9.799416	7.8918123	10.13363	12.09352	11.14172	12.16965	Up_tDCS
**Car5b**	0.21271	7.67E-01	NA	4.907856	4.899708	3.9459061	5.066816	5.038966	5.06442	6.084827	Up_tDCS
**Dmgdh**	0.21273	8.54E-01	NA	0.981571	2.939825	1.9729531	2.026726	2.015586	3.038652	2.028276	Up_tDCS
**Vdac3**	0.21378	9.90E-02	1.82E-01	122.6964	124.4526	121.33661	142.8842	135.0443	145.8553	146.0358	Up_tDCS
**Mdh2**	0.21441	6.11E-02	1.36E-01	166.8671	175.4095	160.79568	197.6058	187.4495	193.4608	199.7852	Up_tDCS
**Acot7**	0.2145	6.62E-02	1.42E-01	156.0698	161.6904	151.91739	184.4321	178.3794	182.3191	181.5307	Up_tDCS
**Atp5g1**	0.2195	6.26E-02	1.37E-01	155.0882	166.5901	150.93091	177.3386	179.3872	190.4222	186.6014	Up_tDCS
**Ucp2**	0.22292	4.13E-01	NA	28.46556	33.31801	28.60782	33.44099	37.28835	29.37364	40.56551	Up_tDCS
**Ndufv2**	0.22349	8.01E-02	1.58E-01	126.6227	127.3924	130.2149	146.9377	143.1066	148.894	159.2196	Up_tDCS
**Ndufb10**	0.22529	8.24E-02	1.61E-01	119.7517	128.3723	119.36366	138.8308	141.091	146.8682	146.0358	Up_tDCS
**Ifi27**	0.22714	2.28E-01	NA	79.50726	104.8537	105.55299	143.8976	91.70917	125.5976	91.27241	Up_tDCS
**Ndufb5**	0.22946	1.59E-01	NA	71.65469	78.39533	72.012787	88.1626	81.63124	88.12091	89.24413	Up_tDCS
**Fkbp8**	0.22961	3.32E-02	8.63E-02	194.3511	197.9482	184.47111	230.0335	219.6989	229.9247	222.0962	Up_tDCS
**Ndufb7**	0.22999	7.93E-02	1.57E-01	119.7517	140.1316	117.39071	146.9377	145.1222	150.9197	147.05	Up_tDCS
**Fis1**	0.23006	5.88E-02	1.34E-01	158.033	162.6703	136.13376	179.3653	178.3794	173.2032	183.559	Up_tDCS
**Hspe1**	0.23072	1.27E-01	NA	83.43355	89.17468	84.836982	102.3497	97.75593	101.2884	101.4138	Up_tDCS
**Prdx5**	0.23119	5.24E-02	1.22E-01	151.162	152.8709	147.97148	173.2851	169.3092	186.3707	178.4883	Up_tDCS
**Vdac2**	0.23397	8.52E-02	1.63E-01	107.9728	109.7535	109.4989	125.657	122.9508	132.6878	131.8379	Up_tDCS
**Iscu**	0.23462	8.19E-02	1.60E-01	114.8438	120.5328	104.56651	131.7372	131.0131	136.7393	133.8662	Up_tDCS
**Mtch1**	0.23466	2.53E-02	7.25E-02	216.9272	222.4467	197.29531	253.3408	243.8859	248.1566	253.5345	Up_tDCS
**Nme6**	0.23571	6.86E-01	NA	7.852569	7.839533	4.9323827	6.080179	7.054552	9.115956	10.14138	Up_tDCS
**Uqcrq**	0.23881	2.80E-01	NA	42.20756	44.09737	38.472585	48.64144	48.37407	50.6442	48.67862	Up_tDCS
**Mrpl27**	0.2396	1.44E-01	NA	73.61784	81.33515	70.039834	82.08242	85.66241	95.2111	91.27241	Up_tDCS
**Guk1**	0.24058	5.84E-02	1.34E-01	132.5121	143.0715	125.28252	153.0179	154.1923	154.9713	169.361	Up_tDCS
**Cox7a2l**	0.24189	6.42E-02	1.39E-01	123.678	127.3924	115.41775	137.8174	140.0832	150.9197	149.0783	Up_tDCS
**Atpif1**	0.24348	3.11E-02	8.32E-02	183.5538	194.0284	166.71453	216.8597	210.6288	223.8474	207.8983	Up_tDCS
**Ndufc2**	0.24421	6.86E-02	1.46E-01	114.8438	116.613	107.52594	135.7907	132.0209	138.7651	128.7955	Up_tDCS
**Mrpl42**	0.24488	1.59E-01	NA	65.76527	74.47556	62.148022	71.94879	78.60786	85.08226	84.17344	Up_tDCS
**Bphl**	0.24556	5.37E-01	NA	12.76042	12.73924	16.770101	16.21381	13.10131	19.2448	18.25448	Up_tDCS
**Chchd1**	0.24662	1.74E-01	NA	59.87584	60.75638	57.215639	67.89534	68.52993	74.95342	69.97551	Up_tDCS
**Slc25a3**	0.24999	1.85E-02	5.68E-02	203.1852	207.7476	199.26826	240.1671	227.7612	246.1308	253.5345	Up_tDCS
**Chchd2**	0.2501	4.42E-02	1.09E-01	139.3831	143.0715	127.25547	162.1381	159.2313	168.1387	160.2338	Up_tDCS
**Atp5g2**	0.25268	3.17E-02	8.42E-02	174.7197	176.3895	148.95796	198.6192	194.5041	194.4737	206.8841	Up_tDCS
**Ndufb6**	0.253	8.14E-02	NA	98.15711	100.934	88.782888	108.4299	111.865	120.5332	116.6259	Up_tDCS
**Ndufb11**	0.25353	7.93E-02	NA	93.24926	98.9741	91.742318	114.51	107.8339	115.4688	113.5834	Up_tDCS
**Tomm7**	0.26077	6.00E-02	1.35E-01	101.1018	112.6933	103.58004	119.5769	128.9975	132.6878	125.7531	Up_tDCS
**Uqcrb**	0.26123	2.20E-01	NA	45.15227	48.01714	40.445538	49.6548	53.41304	55.70862	54.76344	Up_tDCS
**Cox4i2**	0.26165	5.88E-01	NA	8.83414	11.7593	7.8918123	9.120269	11.08572	13.16749	12.16965	Up_tDCS
**Ndufb8**	0.26215	1.91E-02	5.83E-02	173.7381	191.0886	168.68749	211.7929	210.6288	216.7572	213.9831	Up_tDCS
**Atp5d**	0.26536	1.35E-02	4.46E-02	199.2589	212.6473	185.45759	241.1805	234.8158	244.1051	237.3083	Up_tDCS
**Tomm6**	0.26647	4.84E-02	1.15E-01	109.936	119.5529	105.55299	129.7105	136.0521	138.7651	132.8521	Up_tDCS
**Cbr3**	0.27383	6.10E-01	NA	7.852569	7.839533	6.9053357	8.106906	10.07793	9.115956	9.127241	Up_tDCS
**Atp5e**	0.27598	5.72E-02	NA	89.32297	101.9139	92.728794	110.4566	111.865	117.4945	118.6541	Up_tDCS
**Cox8a**	0.28147	1.34E-03	7.40E-03	335.6973	346.8993	314.68601	399.2651	395.0549	415.2825	406.6693	Up_tDCS
**Atp5a1**	0.28643	3.33E-03	1.53E-02	235.5771	243.0255	249.57856	291.8486	287.221	305.891	299.1707	Up_tDCS
**Ndufa6**	0.28671	2.65E-02	7.44E-02	123.678	141.1116	120.35014	154.0312	149.1534	165.1001	158.2055	Up_tDCS
**Slc25a4**	0.28993	2.20E-03	1.10E-02	255.2085	272.4238	256.4839	322.2495	310.4003	325.1358	320.4676	Up_tDCS
**Prdx2**	0.29022	9.31E-03	3.33E-02	170.7934	188.1488	173.61987	209.7662	212.6443	225.8731	220.0679	Up_tDCS
**Chdh**	0.29038	7.05E-01	NA	3.926285	3.919766	3.9459061	4.053453	5.038966	5.06442	5.070689	Up_tDCS
**Mgst3**	0.29101	1.40E-02	4.56E-02	146.2541	156.7907	147.97148	185.4455	175.356	184.3449	190.6579	Up_tDCS
**Ndufa1**	0.29191	7.21E-02	NA	73.61784	78.39533	68.066881	90.18933	86.67021	92.17245	90.25827	Up_tDCS
**Ndufb2**	0.29399	2.10E-02	6.25E-02	131.5305	140.1316	123.30957	157.0713	151.169	171.1774	166.3186	Up_tDCS
**Slc25a13**	0.29521	6.51E-01	NA	5.889427	6.859591	3.9459061	7.093543	8.062345	4.051536	8.113103	Up_tDCS
**Uqcr11**	0.30049	3.50E-03	1.57E-02	223.7982	248.9052	224.91665	290.8353	290.2444	298.8008	265.7041	Up_tDCS
**Cox5a**	0.30165	5.05E-03	2.04E-02	188.4617	199.9081	192.36292	235.1003	225.7457	249.1695	244.4072	Up_tDCS
**Timm8b**	0.30465	1.62E-02	5.08E-02	122.6964	132.2921	122.32309	158.0847	154.1923	155.9841	153.1348	Up_tDCS
**Ndufa8**	0.3047	7.43E-03	2.78E-02	170.7934	207.7476	177.56578	220.9132	227.7612	232.9633	234.2658	Up_tDCS
**Atp5o**	0.30885	6.73E-03	2.62E-02	163.9224	176.3895	157.83625	204.6994	199.543	207.6412	210.9407	Up_tDCS
**Hint1**	0.30937	5.64E-03	2.27E-02	173.7381	184.229	165.72806	214.833	208.6132	221.8216	220.0679	Up_tDCS
**Atp5h**	0.31035	4.51E-03	1.89E-02	182.5722	192.0685	185.45759	223.9533	221.7145	233.9762	246.4355	Up_tDCS
**Cox5b**	0.31182	3.81E-03	1.65E-02	206.1299	235.186	193.3494	264.4878	263.034	266.3885	256.5769	Up_tDCS
**Uqcrh**	0.31912	6.82E-03	2.64E-02	148.2172	160.7104	147.97148	181.392	187.4495	195.4866	195.7286	Up_tDCS

**Ndufb4**	0.32238	6.31E-03	2.48E-02	157.0514	162.6703	144.02557	186.4588	187.4495	199.5382	199.7852	Up_tDCS
**Prdx6**	0.32621	1.19E-02	4.05E-02	119.7517	130.3322	117.39071	144.9109	152.1768	153.9584	163.2762	Up_tDCS
**Cox17**	0.32734	1.45E-02	4.62E-02	118.7701	114.6532	106.53947	135.7907	140.0832	152.9455	139.951	Up_tDCS
**Atp5j**	0.33407	4.84E-03	1.99E-02	152.1435	159.7305	139.09319	189.4989	185.4339	192.448	190.6579	Up_tDCS
**Cox6a1**	0.33419	3.18E-04	2.33E-03	317.0475	326.3205	280.15934	396.225	384.977	394.0119	377.2593	Up_tDCS
**Cox7c**	0.34156	1.36E-03	7.47E-03	201.2221	197.9482	185.45759	254.3542	236.8314	254.2339	242.3789	Up_tDCS
**Qdpr**	0.34955	4.90E-03	1.99E-02	194.3511	183.2491	157.83625	202.6726	256.9872	222.8345	227.1669	Up_tDCS
**Ndufb9**	0.34985	1.57E-03	8.32E-03	171.7749	185.209	167.70101	220.9132	219.6989	228.9118	222.0962	Up_tDCS
**Atp5b**	0.35431	1.96E-05	3.09E-04	352.384	363.5583	346.25326	453.9867	447.4601	456.8107	452.3055	Up_tDCS
**Atp5l**	0.3617	1.73E-03	9.01E-03	164.904	176.3895	151.91739	203.686	205.5898	218.783	217.0255	Up_tDCS
**Sod1**	0.36452	1.38E-03	7.47E-03	168.8302	174.4296	154.87682	206.7261	214.6599	223.8474	209.9265	Up_tDCS
**Ndufa4**	0.37239	4.17E-03	1.78E-02	117.7885	127.3924	115.41775	154.0312	146.13	166.113	156.1772	Up_tDCS
**Ldhb**	0.3759	5.78E-05	6.37E-04	284.6556	294.9624	267.33514	355.6905	354.7432	368.6898	386.3865	Up_tDCS
**Gpx4**	0.37661	6.77E-04	4.33E-03	181.5907	192.0685	164.74158	228.0067	233.808	238.0277	232.2376	Up_tDCS
**Cox4i1**	0.38123	3.45E-05	4.46E-04	284.6556	311.6214	280.15934	372.9177	379.938	397.0505	372.1886	Up_tDCS
**Nmnat3**	0.38679	5.97E-01	NA	2.944713	5.879649	3.9459061	6.080179	5.038966	6.077304	5.070689	Up_tDCS
**Cox7b**	0.38853	1.24E-03	6.87E-03	156.0698	175.4095	150.93091	202.6726	193.4963	227.8989	218.0396	Up_tDCS
**Atp5j2**	0.38907	3.87E-04	2.78E-03	186.4985	207.7476	179.53873	239.1537	243.8859	262.337	256.5769	Up_tDCS
**Dbi**	0.3931	1.57E-01	NA	26.50242	27.43836	24.661913	32.42762	34.26497	33.42517	37.5231	Up_tDCS
**Ndufs5**	0.39652	2.90E-03	1.36E-02	108.9544	123.4726	109.4989	141.8709	145.1222	156.997	156.1772	Up_tDCS
**Cox6b1**	0.39848	2.08E-04	1.70E-03	183.5538	190.1087	178.55225	240.1671	239.8548	253.221	237.3083	Up_tDCS
**Cox6b2**	0.40488	5.60E-01	NA	3.926285	4.899708	4.9323827	5.066816	7.054552	5.06442	7.098965	Up_tDCS
**Cox6c**	0.41493	1.28E-04	1.17E-03	195.3327	210.6874	185.45759	248.274	260.0106	282.5946	260.6334	Up_tDCS
**Mthfs**	0.41615	4.45E-01	NA	6.870998	8.819474	5.9188592	8.106906	10.07793	12.15461	8.113103	Up_tDCS
**Casp8**	0.43393	7.49E-01	NA	0.981571	0.979942	1.9729531	1.013363	2.015586	2.025768	2.028276	Up_tDCS
**Cox7a2**	0.43602	7.02E-04	4.45E-03	124.6595	128.3723	118.37718	156.0579	162.2547	168.1387	183.559	Up_tDCS
**Fth1**	0.53097	6.49E-10	4.62E-08	525.1406	614.4234	496.1977	801.5703	771.9695	798.1526	779.872	Up_tDCS
**Tspo**	0.55714	3.07E-01	NA	6.870998	6.859591	6.9053357	8.106906	9.070138	14.18038	9.127241	Up_tDCS
**Adcy10**	0.62631	5.82E-01	NA	2.944713	0	2.9594296	2.026726	3.023379	4.051536	3.042414	Up_tDCS
**Ndufa13**	0.76382	8.41E-01	NA	0	0	0	0	0	0	1.014138	Up_tDCS
**Fars2**	0.94924	5.96E-01	NA	0	0.979942	0.9864765	2.026726	1.007793	1.012884	1.014138	Up_tDCS
**Cps1**	1.52339	4.75E-01	NA	0.981571	0	0	1.013363	1.007793	1.012884	1.014138	Up_tDCS

Supplementary Table S5: Expression profiles of seventeen 'mitochondria-related' leading-edge genes common to the five mitochondrial pathways found to be significant by the GSEA.

Gene name	log2Fold Change	pvalue	padj	Control Sample16	Control Sample15	Control Sample13	250tDCS Sample8	250tDCS Sample7	250tDCS Sample6	250tDCS Sample5	Up/Down tDCS
**Ndufb9**	0.349852	1.57E-03	8.32E-03	171.77495	185.208955	167.70101	220.9132	219.6989	228.9118	222.0962	Up_tDCS
**Uqcrh**	0.319118	6.82E-03	2.64E-02	148.21724	160.710416	147.97148	181.392	187.4495	195.4866	195.7286	Up_tDCS
**Ndufa8**	0.304696	7.43E-03	2.78E-02	170.79338	207.747611	177.56578	220.9132	227.7612	232.9633	234.2658	Up_tDCS
**Ndufb8**	0.26215	1.91E-02	5.83E-02	173.73809	191.088605	168.68749	211.7929	210.6288	216.7572	213.9831	Up_tDCS
**Ndufc2**	0.244207	6.86E-02	1.46E-01	114.84382	116.613046	107.52594	135.7907	132.0209	138.7651	128.7955	Up_tDCS
**Ndufv2**	0.223489	8.01E-02	1.58E-01	126.62268	127.392403	130.2149	146.9377	143.1066	148.894	159.2196	Up_tDCS
**Uqcrc1**	0.191238	1.09E-01	1.95E-01	150.18038	152.870884	145.01205	169.2317	168.3015	171.1774	173.4176	Up_tDCS
**Sdhc**	0.194621	1.48E-01	2.32E-01	113.86225	122.492695	108.51242	130.7239	127.9897	131.6749	135.8945	Up_tDCS
**Ndufa7**	0.178173	2.00E-01	2.83E-01	116.80696	117.592988	99.63413	129.7105	131.0131	123.5719	119.6683	Up_tDCS
**Ndufa12**	0.172259	2.15E-01	2.97E-01	106.00968	111.713338	103.58004	118.5635	114.8884	126.6105	122.7107	Up_tDCS
**Uqcrfs1**	0.162199	2.29E-01	3.10E-01	113.86225	119.552871	110.48537	129.7105	121.943	130.662	130.8238	Up_tDCS
**Ndufv1**	0.150799	2.29E-01	3.10E-01	136.43839	141.111585	129.22843	153.0179	149.1534	151.9326	148.0641	Up_tDCS
**Ndufb6**	0.253005	8.14E-02	0.00E+00	98.157113	100.933981	88.782888	108.4299	111.865	120.5332	116.6259	Up_tDCS
**Ndufs8**	0.157001	2.71E-01	0.00E+00	101.10183	107.793572	94.701747	114.51	111.865	113.443	111.5552	Up_tDCS
**Sdhd**	0.154606	2.92E-01	0.00E+00	94.230829	98.9740978	89.769365	106.4031	101.7871	105.3399	106.4845	Up_tDCS
**Uqcrb**	0.261226	2.20E-01	0.00E+00	45.152272	48.0171366	40.445538	49.6548	53.41304	55.70862	54.76344	Up_tDCS
**Uqcrq**	0.238805	2.80E-01	0.00E+00	42.207559	44.0973703	38.472585	48.64144	48.37407	50.6442	48.67862	Up_tDCS

Supplementary Table S6: Expression profiles of genes in the 'TCA cycle' pathway.

Gene name	log2Fold Change	pvalue	padj	Control Sample16	Control Sample15	Control Sample13	250tDCS Sample8	250tDCS Sample7	250tDCS Sample6	250tDCS Sample5	upregulated/ downregulated
**Dlat**	-0.1285	0.5690	NA	46.1338	41.1575	51.2968	43.5746	42.3273	41.5282	41.5797	down
**Pck2**	-0.0315	0.9110	NA	28.4656	30.3782	29.5943	27.3608	29.2260	31.3994	27.3817	down
**Ogdhl**	-0.0263	0.9140	NA	38.2813	40.1776	37.4861	42.5613	37.2883	36.4638	35.4948	down
**Aco1**	-0.0182	0.9520	NA	25.5208	25.4785	26.6349	25.3341	26.2026	25.3221	25.3534	down
**Pc**	-0.0059	0.9740	NA	61.8390	65.6561	66.0939	66.8820	63.4910	61.7859	64.9048	down
**Acly**	0.0069	0.9670	NA	76.5625	71.5357	73.9857	74.9889	74.5767	74.9534	73.0179	up
**Dld**	0.0295	0.8620	NA	74.5994	68.5959	76.9452	79.0423	71.5533	70.9019	78.0886	up
**Ogdh**	0.0461	0.7530	NA	96.1940	96.0343	102.5936	104.3764	101.7871	98.2498	101.4138	up
**Dlst**	0.0725	0.6550	NA	77.5441	78.3953	77.9316	79.0423	80.6234	85.0823	83.1593	up
**Idh1**	0.0770	0.7270	NA	43.1891	44.0974	44.3914	45.6013	48.3741	43.5540	47.6645	up
**Idh3a**	0.0869	0.5420	NA	104.0465	100.9340	101.6071	108.4299	105.8183	109.3915	110.5410	up
**Idh3a**	0.0869	0.5420	NA	104.0465	100.9340	101.6071	108.4299	105.8183	109.3915	110.5410	up
**Cs**	0.0963	0.4520	0.5130	128.5858	127.3924	138.1067	140.8575	137.0599	142.8166	140.9652	up
**Idh2**	0.1093	0.5720	NA	55.9496	58.7965	51.2968	59.7884	59.4598	60.7730	58.8200	up
**Sucla2**	0.1096	0.4430	NA	101.1018	101.9139	101.6071	109.4432	103.8027	112.4301	112.5693	up
**Sdha**	0.1180	0.3510	0.4200	130.5490	133.2721	134.1608	145.9243	142.0988	145.8553	141.9793	up
**Suclg2**	0.1217	0.5710	NA	44.1707	51.9369	43.4050	47.6281	51.3974	48.6184	54.7634	up
**Pdha1**	0.1355	0.3410	NA	103.0650	98.9741	100.6206	112.4833	107.8339	112.4301	110.5410	up
**Idh3B**	0.1492	0.2590	0.3380	117.7885	124.4526	116.4042	130.7239	128.9975	135.7265	134.8803	up
**Sdhd**	0.1546	0.2920	NA	94.2308	98.9741	89.7694	106.4031	101.7871	105.3399	106.4845	up
**Idh3g**	0.1556	0.2390	0.3200	118.7701	122.4927	114.4313	133.7639	132.0209	132.6878	129.8096	up
**Pdhb**	0.1669	0.3100	NA	71.6547	76.4354	72.9993	83.0958	79.6157	85.0823	83.1593	up
**Suclg1**	0.1829	0.1750	0.2610	110.9175	117.5930	110.4854	125.6570	124.9663	130.6620	131.8379	up
**Sdhb**	0.1833	0.1520	0.2370	133.4937	135.2319	122.3231	153.0179	142.0988	145.8553	151.1065	up
**Aco2**	0.1925	0.0968	0.1800	159.0145	161.6904	156.8498	180.3787	180.3950	182.3191	184.5731	up
**Sdhc**	0.1946	0.1480	0.2320	113.8623	122.4927	108.5124	130.7239	127.9897	131.6749	135.8945	up
**Mdh2**	0.2144	0.0611	0.1360	166.8671	175.4095	160.7957	197.6058	187.4495	193.4608	199.7852	up
**Mdh1**	0.3976	0.0000	0.0003	261.0979	284.1831	275.2270	356.7039	345.6730	366.6640	372.1886	up

Supplementary Table S7: Expression profiles of 'calcium related' genes. The genes belonging to the 'calcium related' pathway was identified utilizing the CaGeDb database.

Gene name	log2Fold Change	pvalue	padj	Control Sample16	Control Sample15	Control Sample13	250tDCS Sample8	250tDCS Sample7	250tDCS Sample6	250tDCS Sample5	Up/Down tDCS
**Apoe**	0.487374	2.32E-08	8.89E-07	814.704	848.62939	671.7905	1133.953	1069.268	1079.734	1082.085	Up_tDCS
**Actg1**	0.471818	2.70E-07	8.69E-06	295.4529	303.78188	269.3081	409.3988	383.9692	406.1665	406.6693	Up_tDCS
**Sparcl1**	0.407426	3.32E-07	1.03E-05	425.0203	434.11411	429.1173	579.6438	552.2706	546.9574	599.3555	Up_tDCS
**Snap25**	0.409237	1.33E-06	3.14E-05	462.32	445.87341	531.7109	643.4857	622.8161	655.336	627.7513	Up_tDCS
**S100b**	0.484268	9.68E-06	1.81E-04	340.6052	391.97663	289.0376	449.9333	482.7329	456.8107	516.1962	Up_tDCS
**Calm2**	0.376448	1.14E-05	2.08E-04	326.8632	341.01966	325.5373	430.6794	414.203	440.6046	434.051	Up_tDCS
**Thy1**	0.34118	5.44E-05	6.11E-04	333.7342	341.01966	346.2533	434.7328	432.3432	419.334	438.1075	Up_tDCS
**Tpt1**	0.43644	7.85E-05	8.09E-04	226.7429	238.1258	216.0384	280.7016	305.3613	291.7106	350.8917	Up_tDCS
**Calm1**	0.337551	8.64E-05	8.59E-04	340.6052	347.87925	366.9693	434.7328	433.351	467.9524	442.1641	Up_tDCS
**Nrgn**	0.263065	3.74E-04	2.71E-03	600.7215	670.28003	603.7236	752.9289	740.7279	747.5084	758.5751	Up_tDCS
**Atp1b1**	0.307652	5.20E-04	3.51E-03	310.1765	308.68159	317.6454	392.1716	366.8367	395.0248	391.4572	Up_tDCS
**App**	0.429425	5.59E-04	3.69E-03	125.6411	121.51275	132.1879	166.1916	169.3092	170.1645	175.4458	Up_tDCS
**Sncb**	0.291788	8.85E-04	5.40E-03	334.7158	360.6185	319.6184	413.4522	412.1874	421.3598	409.7117	Up_tDCS
**Ywhae**	0.316916	1.07E-03	6.28E-03	249.3191	254.78481	246.6191	309.0758	294.2756	322.0971	321.4817	Up_tDCS
**Myl6b**	0.360604	2.45E-03	1.20E-02	142.3278	154.83077	139.0932	185.4455	185.4339	191.4351	184.5731	Up_tDCS
**Prnp**	0.298758	2.69E-03	1.27E-02	231.6508	239.10574	230.8355	278.6749	279.1587	303.8652	289.0293	Up_tDCS
**Pebp1**	0.278447	3.98E-03	1.70E-02	275.8215	288.10282	249.5786	334.4099	317.4548	335.2646	328.5807	Up_tDCS
**Vsnl1**	0.271453	5.96E-03	2.39E-02	249.3191	251.84498	237.7408	299.9555	278.1509	302.8523	308.2979	Up_tDCS
**Pcp4**	0.308395	6.99E-03	2.67E-02	165.8855	187.16884	161.7822	205.7127	211.6366	220.8087	211.9548	Up_tDCS
**Sparc**	0.303233	1.05E-02	3.65E-02	166.8671	185.20896	152.9039	196.5925	210.6288	202.5768	221.0821	Up_tDCS
**Hpca**	0.247256	1.95E-02	5.91E-02	205.1484	216.56709	197.2953	251.3141	231.7924	247.1437	249.4779	Up_tDCS
**Gdi1**	0.232965	2.55E-02	7.25E-02	206.1299	206.76767	205.1871	239.1537	234.8158	249.1695	245.4214	Up_tDCS
**Ppp1r9b**	0.240676	2.81E-02	7.68E-02	181.5907	188.14878	177.5658	220.9132	217.6833	212.7056	210.9407	Up_tDCS
**Fkbp1a**	0.246017	2.94E-02	8.01E-02	177.6644	185.20896	167.701	215.8464	197.5275	217.7701	207.8983	Up_tDCS
**Calr**	0.239733	3.11E-02	8.32E-02	179.6275	189.12872	181.5117	208.7528	205.5898	225.8731	226.1527	Up_tDCS
**Pvalb**	0.272987	3.21E-02	8.45E-02	137.42	146.99123	137.1202	169.2317	150.1612	173.2032	186.6014	Up_tDCS
**Npy**	0.311243	3.80E-02	9.78E-02	94.23083	106.81363	90.75584	134.7773	106.8261	113.443	127.7814	Up_tDCS
**Arf1**	0.222672	4.13E-02	1.03E-01	181.5907	190.10866	182.4982	216.8597	210.6288	220.8087	213.9831	Up_tDCS
**Gap43**	0.227489	5.45E-02	1.27E-01	155.0882	164.63018	150.9309	191.5257	179.3872	189.4093	174.4317	Up_tDCS
**Fis1**	0.230064	5.88E-02	1.34E-01	158.033	162.6703	136.1338	179.3653	178.3794	173.2032	183.559	Up_tDCS
**Syn1**	0.195204	7.13E-02	1.49E-01	192.3879	191.0886	195.3224	230.0335	211.6366	224.8603	217.0255	Up_tDCS
**Nptn**	0.222575	7.68E-02	1.55E-01	126.6227	137.19182	149.9444	155.0446	161.2469	164.0872	163.2762	Up_tDCS
**Stxbp1**	0.174503	8.03E-02	1.58E-01	233.6139	229.30633	244.6462	268.5413	260.0106	269.4272	266.7183	Up_tDCS
**Got1**	0.20085	8.39E-02	1.62E-01	163.9224	166.59007	156.8498	185.4455	177.3716	191.4351	192.6862	Up_tDCS
**Atf4**	0.211371	8.82E-02	1.67E-01	135.4568	145.03135	137.1202	155.0446	155.2001	166.113	168.3469	Up_tDCS
**Rgs4**	0.197264	1.02E-01	1.86E-01	155.0882	155.81071	157.8362	165.1782	172.3326	185.3578	193.7003	Up_tDCS
**Capns1**	0.222115	1.04E-01	1.88E-01	117.7885	118.57293	108.5124	125.657	128.9975	133.7007	148.0641	Up_tDCS
**Gnai2**	0.212567	1.09E-01	1.95E-01	122.6964	129.35229	109.4989	138.8308	139.0755	136.7393	144.0076	Up_tDCS
**Atp1a2**	0.176987	1.13E-01	1.97E-01	201.2221	208.72755	198.2818	228.0067	250.9405	206.6283	231.2234	Up_tDCS
**Hsp90b1**	0.204993	1.14E-01	1.98E-01	125.6411	127.3924	126.269	137.8174	138.0677	154.9713	152.1207	Up_tDCS
**Cx3cl1**	0.178437	1.19E-01	2.04E-01	161.9592	170.50983	174.6063	197.6058	193.4963	187.3835	186.6014	Up_tDCS
**Pink1**	0.187443	1.25E-01	2.12E-01	152.1435	149.93106	135.1473	166.1916	168.3015	160.0357	169.361	Up_tDCS
**Dlg4**	0.172877	1.34E-01	2.20E-01	161.9592	170.50983	163.7551	190.5123	184.4261	191.4351	179.5024	Up_tDCS
**Snca**	0.198301	1.39E-01	2.26E-01	136.4384	128.37234	140.0797	170.245	149.1534	167.1259	132.8521	Up_tDCS
**Ddx5**	0.184254	1.43E-01	2.29E-01	135.4568	130.33223	141.0661	150.9911	148.1456	164.0872	153.1348	Up_tDCS
**Ppp3r1**	0.168894	1.66E-01	2.53E-01	149.1988	145.03135	145.9985	169.2317	155.2001	174.2161	161.2479	Up_tDCS
**Hspa5**	0.184542	1.76E-01	2.62E-01	112.8807	111.71334	117.3907	122.617	121.943	138.7651	134.8803	Up_tDCS
**Camk2b**	0.153278	1.77E-01	2.62E-01	167.8487	170.50983	175.5928	196.5925	190.4729	187.3835	187.6155	Up_tDCS
**Nucb1**	0.150061	2.13E-01	2.96E-01	147.2357	146.01129	147.9715	163.1515	160.2391	165.1001	164.2903	Up_tDCS
**Pde2a**	0.164551	2.29E-01	3.10E-01	105.0281	115.6331	110.4854	124.6437	125.9741	123.5719	120.6824	Up_tDCS
**Stx1a**	0.132869	2.81E-01	3.58E-01	146.2541	157.77059	141.0661	171.2584	159.2313	166.113	154.149	Up_tDCS
**Gja1**	0.155694	2.85E-01	3.61E-01	112.8807	107.79357	92.72879	107.4165	116.904	114.4559	126.7672	Up_tDCS
**Gnao1**	0.108309	3.35E-01	4.09E-01	174.7197	175.40954	186.4441	191.5257	191.4807	195.4866	192.6862	Up_tDCS
**Grin1**	0.111929	3.67E-01	4.35E-01	136.4384	139.1517	143.0391	152.0045	146.13	152.9455	152.1207	Up_tDCS
**Slc6a1**	0.113488	3.94E-01	4.63E-01	119.7517	119.55287	116.4042	130.7239	128.9975	122.559	130.8238	Up_tDCS
**Stx1b**	0.091826	4.30E-01	4.93E-01	161.9592	164.63018	167.701	179.3653	176.3638	169.1516	177.4741	Up_tDCS

**Jph3**	0.094015	4.89E-01	5.42E-01	114.8438	118.57293	111.4718	128.6971	123.9586	119.5203	118.6541	Up_tDCS
**Gnb1**	0.087292	5.09E-01	5.62E-01	141.3462	126.41246	130.2149	133.7639	131.0131	154.9713	144.0076	Up_tDCS
**Ppp3cb**	0.059941	6.62E-01	6.96E-01	115.8254	108.77351	118.3772	124.6437	114.8884	121.5461	115.6117	Up_tDCS
**Cplx2**	0.024567	8.49E-01	8.66E-01	136.4384	129.35229	131.2014	141.8709	131.0131	131.6749	133.8662	Up_tDCS
**Atp2a2**	-0.02233	8.62E-01	8.79E-01	155.0882	134.25199	164.7416	159.098	151.169	144.8424	140.9652	Down_tDCS
**Ppp3ca**	0.020383	8.76E-01	8.89E-01	131.5305	127.3924	145.9985	143.8976	131.0131	133.7007	138.9369	Up_tDCS
**A2m**	0.678646	3.21E-01	0.00E+00	3.926285	4.8997078	4.932383	6.080179	4.031172	8.103072	11.15552	Up_tDCS
**Arsa**	-0.16534	5.00E-01	0.00E+00	41.22599	41.157546	38.47258	34.45435	40.31172	33.42517	35.49482	Down_tDCS
**Asah2**	-0.25021	5.59E-01	0.00E+00	11.77885	12.73924	19.72953	12.16036	13.10131	11.14172	13.18379	Down_tDCS
**Aspn**	-1.67884	4.85E-01	0.00E+00	0.981571	0.9799416	0.986477	1.013363	0	0	0	Down_tDCS
**Abl1**	-0.11231	7.03E-01	0.00E+00	30.42871	27.438364	26.63487	26.34744	28.21821	23.29633	26.36758	Down_tDCS
**Acsl4**	-0.18023	6.02E-01	0.00E+00	21.59456	17.638948	29.5943	20.26726	17.13248	23.29633	20.28276	Down_tDCS
**Actn1**	-0.18967	3.65E-01	0.00E+00	50.06013	55.856669	61.16155	50.66816	52.40524	50.6442	41.57965	Down_tDCS
**Actn2**	0.08449	8.19E-01	0.00E+00	18.64985	16.659007	14.79715	17.22718	17.13248	18.23191	18.25448	Up_tDCS
**Actn4**	0.099033	5.80E-01	0.00E+00	74.59941	70.555793	58.20212	78.02897	74.57669	67.86323	69.97551	Up_tDCS
**Adam8**	0.212507	8.51E-01	0.00E+00	1.963142	1.9598831	1.972953	2.026726	2.015586	3.038652	2.028276	Up_tDCS
**Adam9**	-0.17036	5.50E-01	0.00E+00	29.44713	29.398247	32.55373	29.38753	28.21821	25.3221	25.35345	Down_tDCS
**Adcy1**	-0.32783	1.17E-01	0.00E+00	61.83898	52.916844	71.02631	52.69489	56.43641	43.55401	44.62207	Down_tDCS
**Adcy3**	-0.13879	5.93E-01	0.00E+00	35.33656	34.297955	36.49963	33.44099	32.24938	32.41229	30.42414	Down_tDCS
**Adcy5**	-0.10389	6.60E-01	0.00E+00	41.22599	41.157546	42.41849	40.53453	40.31172	36.46383	37.5231	Down_tDCS
**Adcy8**	0.014456	9.73E-01	0.00E+00	12.76042	11.759299	13.81067	12.16036	12.09352	12.15461	15.21207	Up_tDCS
**Adcyap1r1**	-0.10325	6.89E-01	0.00E+00	36.31813	35.277896	38.47258	28.37417	38.29614	33.42517	36.50896	Down_tDCS
**Add1**	0.801168	2.51E-02	0.00E+00	11.77885	19.598831	14.79715	23.30735	22.17145	29.37364	32.45241	Up_tDCS
**Add3**	-0.01955	9.18E-01	0.00E+00	62.82055	59.776435	58.20212	59.78843	60.46759	55.70862	61.86241	Down_tDCS
**Adm**	-0.2345	8.04E-01	0.00E+00	2.944713	3.9197663	2.95943	3.04009	2.015586	3.038652	3.042414	Down_tDCS
**Adora1**	-0.0162	9.27E-01	0.00E+00	69.69155	64.676143	69.05336	67.89534	67.52214	63.81169	68.96137	Down_tDCS
**Adora2a**	-0.00992	9.90E-01	0.00E+00	4.907856	4.8997078	3.945906	5.066816	5.038966	5.06442	3.042414	Down_tDCS
**Adra1a**	-0.6368	5.05E-01	0.00E+00	3.926285	1.9598831	5.918859	3.04009	3.023379	2.025768	2.028276	Down_tDCS
**Afg3l2**	0.023821	9.14E-01	0.00E+00	44.1707	44.09737	45.37792	44.58798	44.3429	45.57978	46.65034	Up_tDCS
**Ager**	-0.37258	6.53E-01	0.00E+00	3.926285	5.8796494	3.945906	3.04009	5.038966	2.025768	4.056551	Down_tDCS
**Agrn**	-0.31615	2.66E-01	0.00E+00	32.39185	29.398247	36.49963	28.37417	26.20262	27.34787	23.32517	Down_tDCS
**Agt**	0.212512	1.48E-01	0.00E+00	101.1018	105.83369	87.79641	105.3898	120.9352	116.4817	112.5693	Up_tDCS
**Aif1**	0.508318	3.96E-02	0.00E+00	32.39185	39.197663	33.5402	40.53453	43.3351	54.69574	60.84827	Up_tDCS
**Akap5**	-0.22412	4.56E-01	0.00E+00	26.50242	25.478481	38.47258	27.36081	26.20262	25.3221	24.33931	Down_tDCS
**Akap6**	-0.26365	3.84E-01	0.00E+00	27.48399	24.498539	35.51316	25.33408	24.18703	24.30922	23.32517	Down_tDCS
**Alg2**	0.087502	5.90E-01	0.00E+00	76.56255	79.375267	76.94517	80.0557	79.61566	85.08226	85.18758	Up_tDCS
**Alox15**	0.130211	8.72E-01	0.00E+00	3.926285	3.9197663	3.945906	5.066816	3.023379	6.077304	3.042414	Up_tDCS
**Alox5ap**	0.240545	7.06E-01	0.00E+00	5.889427	5.8796494	4.932383	6.080179	7.054552	6.077304	7.098965	Up_tDCS
**Ank2**	0.126611	4.77E-01	0.00E+00	65.76527	66.636026	60.17507	71.94879	67.52214	69.889	70.98965	Up_tDCS
**Ano10**	-0.01326	9.60E-01	0.00E+00	32.39185	31.35813	33.5402	31.41426	32.24938	32.41229	32.45241	Down_tDCS
**Ano4**	-0.0474	8.66E-01	0.00E+00	31.41028	29.398247	28.60782	28.37417	29.226	28.36075	29.41	Down_tDCS
**Ano6**	0.042574	9.47E-01	0.00E+00	4.907856	6.8595909	5.918859	6.080179	6.046759	6.077304	6.084827	Up_tDCS
**Anxa1**	-0.07284	9.27E-01	0.00E+00	3.926285	4.8997078	3.945906	5.066816	4.031172	4.051536	3.042414	Down_tDCS
**Anxa11**	-0.03493	8.39E-01	0.00E+00	69.69155	74.475559	72.01279	73.97552	69.53772	70.90188	66.9331	Down_tDCS
**Anxa2**	0.183815	6.14E-01	0.00E+00	15.70514	16.659007	17.75658	17.22718	18.14028	22.28345	18.25448	Up_tDCS
**Anxa3**	0.494514	3.31E-02	0.00E+00	36.31813	46.057253	34.52668	46.61471	50.38966	56.72151	65.91896	Up_tDCS
**Anxa4**	-0.05522	8.14E-01	0.00E+00	39.26285	41.157546	43.40497	40.53453	38.29614	41.52825	38.53724	Down_tDCS
**Anxa5**	0.133437	4.48E-01	0.00E+00	63.80212	68.595909	64.12097	71.94879	68.52993	73.94053	73.01792	Up_tDCS
**Anxa6**	0.019322	9.18E-01	0.00E+00	59.87584	57.816552	68.06688	59.78843	61.47538	61.78593	67.94724	Up_tDCS
**Anxa7**	-0.06911	7.54E-01	0.00E+00	46.13384	46.057253	46.3644	45.60135	42.32731	44.5669	43.60793	Down_tDCS
**Apobec1**	0.951144	5.95E-01	0.00E+00	0.981571	0.9799416	0	1.013363	1.007793	1.012884	2.028276	Up_tDCS
**Atg5**	0.019812	9.52E-01	0.00E+00	20.61299	21.558714	20.71601	20.26726	21.16366	22.28345	21.29689	Up_tDCS
**Atp2a3**	-0.08317	9.32E-01	0.00E+00	2.944713	2.9398247	2.95943	3.04009	3.023379	3.038652	2.028276	Down_tDCS
**Atp2b1**	-0.05827	7.18E-01	0.00E+00	83.43355	80.355208	102.5936	85.12251	83.64683	86.09514	86.20172	Down_tDCS
**Atp2b2**	0.08095	6.72E-01	0.00E+00	55.94955	61.736318	55.24269	56.74834	64.49876	59.76016	62.87655	Up_tDCS
**Atp2b3**	-0.18994	4.01E-01	0.00E+00	46.13384	42.137487	57.21564	42.56126	42.32731	43.55401	41.57965	Down_tDCS
**Atp2c1**	0.007735	9.68E-01	0.00E+00	58.89427	53.896786	63.1345	57.76171	58.452	60.77304	58.82	Up_tDCS
**Atp7b**	-0.5643	6.20E-01	0.00E+00	2.944713	2.9398247	1.972953	2.026726	1.007793	2.025768	2.028276	Down_tDCS
**Avp**	-0.72001	4.60E-01	0.00E+00	8.83414	1.9598831	2.95943	4.053453	2.015586	4.051536	1.014138	Down_tDCS
**Avpr1a**	0.042505	9.79E-01	0.00E+00	0.981571	0.9799416	0.986477	1.013363	1.007793	1.012884	1.014138	Up_tDCS
**Bad**	0.071412	6.83E-01	0.00E+00	68.70998	74.475559	64.12097	69.92206	69.53772	77.99207	73.01792	Up_tDCS
**Bak1**	0.002043	9.96E-01	0.00E+00	18.64985	18.61889	15.78362	17.22718	17.13248	18.23191	18.25448	Up_tDCS
**Bax**	0.111756	5.72E-01	0.00E+00	54.96798	56.836611	48.33735	57.76171	57.44421	57.73439	57.80586	Up_tDCS
**Bcap31**	0.133406	3.97E-01	0.00E+00	81.4704	83.295033	80.89108	86.13588	87.678	90.14668	95.32896	Up_tDCS
**Bcl2**	-0.10977	9.06E-01	0.00E+00	3.926285	2.9398247	2.95943	2.026726	4.031172	3.038652	3.042414	Down_tDCS
**Bdkrb2**	0.044419	9.80E-01	0.00E+00	0.981571	1.9598831	0	1.013363	1.007793	1.012884	1.014138	Up_tDCS
**Best1**	-0.15023	9.02E-01	0.00E+00	1.963142	1.9598831	1.972953	2.026726	2.015586	1.012884	2.028276	Down_tDCS
**Bglap**	-0.19795	9.59E-01	0.00E+00	0.981571	0	0	0	1.007793	0	0	Down_tDCS
**Bin1**	0.208314	2.01E-01	0.00E+00	79.50726	73.495617	74.97222	81.06906	85.66241	89.13379	95.32896	Up_tDCS
**Bmp1**	-0.0924	7.81E-01	0.00E+00	27.48399	20.578773	20.71601	24.32072	22.17145	21.27056	18.25448	Down_tDCS

**Bnip3**	0.155085	3.09E-01	0.00E+00	90.30454	84.274974	85.82346	99.3096	92.71697	98.24975	96.3431	Up_tDCS
**Braf**	-0.65709	2.85E-01	0.00E+00	6.870998	6.8595909	13.81067	4.053453	5.038966	8.103072	6.084827	Down_tDCS
**C1r**	0.276134	5.28E-01	0.00E+00	11.77885	11.759299	12.82419	12.16036	11.08572	21.27056	14.19793	Up_tDCS
**C1s**	0.244252	7.26E-01	0.00E+00	4.907856	4.8997078	4.932383	4.053453	5.038966	8.103072	6.084827	Up_tDCS
**Cabp4**	-0.0729	9.26E-01	0.00E+00	4.907856	3.9197663	3.945906	4.053453	4.031172	4.051536	4.056551	Down_tDCS
**Cacna1a**	-0.1705	3.91E-01	0.00E+00	55.94955	57.816552	69.05336	55.73498	59.45979	52.66997	48.67862	Down_tDCS
**Cacna1b**	-0.53969	1.88E-01	0.00E+00	16.68671	14.699123	22.68896	15.20045	13.10131	11.14172	10.14138	Down_tDCS
**Cacna1c**	-0.61394	4.87E-01	0.00E+00	3.926285	3.9197663	4.932383	3.04009	3.023379	3.038652	2.028276	Down_tDCS
**Cacna1d**	-0.58455	3.80E-01	0.00E+00	6.870998	5.8796494	8.878289	6.080179	5.038966	5.06442	3.042414	Down_tDCS
**Cacna1g**	-0.37302	3.41E-01	0.00E+00	14.72357	16.659007	23.67544	15.20045	15.1169	13.16749	13.18379	Down_tDCS
**Cacna1h**	-0.37326	4.83E-01	0.00E+00	10.79728	9.7994156	11.83772	7.093543	12.09352	10.12884	4.056551	Down_tDCS
**Cacna2d1**	-0.23548	4.43E-01	0.00E+00	25.52085	24.498539	38.47258	25.33408	24.18703	26.33498	24.33931	Down_tDCS
**Cacna2d2**	-0.29184	4.55E-01	0.00E+00	15.70514	15.679065	19.72953	14.18709	14.1091	13.16749	14.19793	Down_tDCS
**Cacna2d3**	0.240684	2.11E-01	0.00E+00	50.06013	52.916844	62.14802	61.81516	61.47538	67.86323	68.96137	Up_tDCS
**Cacnb2**	-0.02235	9.36E-01	0.00E+00	29.44713	29.398247	30.58077	30.4009	28.21821	30.38652	28.39586	Down_tDCS
**Cacnb3**	0.098393	5.02E-01	0.00E+00	95.2124	104.85375	92.72879	108.4299	102.7949	102.3013	104.4562	Up_tDCS
**Cacnb4**	-0.35804	2.29E-01	0.00E+00	32.39185	25.478481	37.48611	28.37417	24.18703	23.29633	23.32517	Down_tDCS
**Cacng2**	-0.20991	3.71E-01	0.00E+00	42.20756	42.137487	47.35087	39.52117	38.29614	36.46383	37.5231	Down_tDCS
**Cadps2**	-0.05245	7.95E-01	0.00E+00	53.98641	49.97702	62.14802	51.68153	51.39745	54.69574	55.77758	Down_tDCS
**Calb1**	-0.08305	7.31E-01	0.00E+00	36.31813	42.137487	45.37792	44.58798	35.27276	39.50248	36.50896	Down_tDCS
**Calb2**	0.014489	9.69E-01	0.00E+00	14.72357	18.61889	17.75658	18.24054	17.13248	16.20614	17.24034	Up_tDCS
**Calca**	0.057798	9.19E-01	0.00E+00	6.870998	7.8395325	7.891812	7.093543	10.07793	6.077304	8.113103	Up_tDCS
**Calcrl**	0.393208	6.40E-01	0.00E+00	3.926285	2.9398247	2.95943	5.066816	4.031172	3.038652	5.070689	Up_tDCS
**Cald1**	0.063758	8.88E-01	0.00E+00	11.77885	11.759299	9.864765	12.16036	11.08572	12.15461	11.15552	Up_tDCS
**Calu**	0.027207	9.09E-01	0.00E+00	40.24442	36.257838	40.44554	39.52117	39.30393	41.52825	38.53724	Up_tDCS
**Camk1g**	-0.07577	7.47E-01	0.00E+00	40.24442	44.09737	40.44554	38.5078	40.31172	38.48959	40.56551	Down_tDCS
**Camk4**	-0.20972	4.71E-01	0.00E+00	27.48399	28.418305	34.52668	26.34744	24.18703	26.33498	27.38172	Down_tDCS
**Camkk2**	-0.07735	6.68E-01	0.00E+00	65.76527	67.615968	70.03983	69.92206	63.49097	59.76016	63.89068	Down_tDCS
**Camta1**	-0.1849	6.88E-01	0.00E+00	12.76042	10.779357	11.83772	11.147	10.07793	9.115956	11.15552	Down_tDCS
**Cant1**	-0.41953	4.34E-01	0.00E+00	9.815711	12.73924	7.891812	9.120269	8.062345	8.103072	5.070689	Down_tDCS
**Capn1**	-0.17439	7.15E-01	0.00E+00	11.77885	12.73924	8.878289	9.120269	11.08572	9.115956	10.14138	Down_tDCS
**Capn2**	0.037861	8.53E-01	0.00E+00	50.06013	51.936903	50.3103	49.6548	52.40524	52.66997	53.74931	Up_tDCS
**Capn5**	-0.17394	5.53E-01	0.00E+00	27.48399	28.418305	30.58077	25.33408	28.21821	25.3221	23.32517	Down_tDCS
**Cask**	-0.04673	8.84E-01	0.00E+00	22.57614	20.578773	26.63487	22.29399	22.17145	21.27056	24.33931	Down_tDCS
**Casq1**	0.627159	6.76E-01	0.00E+00	0.981571	0.9799416	0.986477	1.013363	2.015586	1.012884	2.028276	Up_tDCS
**Casq2**	0.042505	9.79E-01	0.00E+00	0.981571	0.9799416	0.986477	1.013363	1.007793	1.012884	1.014138	Up_tDCS
**Casr**	-0.78724	6.49E-01	0.00E+00	0.981571	1.9598831	0.986477	0	1.007793	1.012884	1.014138	Down_tDCS
**Cast**	-0.2594	5.24E-01	0.00E+00	17.66828	12.73924	17.75658	13.17372	11.08572	16.20614	13.18379	Down_tDCS
**Catsper2**	-0.05067	9.51E-01	0.00E+00	3.926285	3.9197663	3.945906	3.04009	4.031172	4.051536	4.056551	Down_tDCS
**Cav1**	0.11833	6.90E-01	0.00E+00	29.44713	26.458422	21.70248	28.37417	27.21041	28.36075	28.39586	Up_tDCS
**Cblb**	-0.30259	6.43E-01	0.00E+00	5.889427	5.8796494	7.891812	5.066816	6.046759	5.06442	5.070689	Down_tDCS
**Ccbe1**	-0.37337	8.09E-01	0.00E+00	0.981571	0.9799416	1.972953	1.013363	1.007793	1.012884	1.014138	Down_tDCS
**Cckbr**	-0.02899	9.05E-01	0.00E+00	42.20756	36.257838	38.47258	39.52117	39.30393	38.48959	35.49482	Down_tDCS
**Ccl19**	0.743791	4.84E-01	0.00E+00	1.963142	2.9398247	0.986477	5.066816	3.023379	3.038652	2.028276	Up_tDCS
**Ccl2**	0.797063	5.65E-01	0.00E+00	0.981571	0.9799416	1.972953	1.013363	1.007793	6.077304	1.014138	Up_tDCS
**Ccl3**	0.628222	7.54E-01	0.00E+00	0	0.9799416	0.986477	1.013363	0	2.025768	1.014138	Up_tDCS
**Ccl4**	0.94857	5.96E-01	0.00E+00	0.981571	0	0.986477	1.013363	1.007793	2.025768	1.014138	Up_tDCS
**Ccl5**	-0.37172	7.91E-01	0.00E+00	1.963142	1.9598831	0.986477	1.013363	1.007793	2.025768	1.014138	Down_tDCS
**Ccl7**	1.211543	5.22E-01	0.00E+00	0.981571	0	0.986477	0	1.007793	4.051536	1.014138	Up_tDCS
**Ccnd1**	0.003029	9.94E-01	0.00E+00	11.77885	12.73924	11.83772	12.16036	12.09352	12.15461	12.16965	Up_tDCS
**Ccr10**	0.11235	9.30E-01	0.00E+00	1.963142	0.9799416	1.972953	2.026726	2.015586	1.012884	2.028276	Up_tDCS
**Ccr5**	0.73738	1.85E-01	0.00E+00	5.889427	6.8595909	5.918859	8.106906	9.070138	10.12884	14.19793	Up_tDCS
**Ccrl2**	0.042505	9.79E-01	0.00E+00	0.981571	0.9799416	0.986477	1.013363	1.007793	1.012884	1.014138	Up_tDCS
**Cd24**	0.069228	7.43E-01	0.00E+00	47.11541	53.896786	44.39144	49.6548	49.38186	51.65709	52.73517	Up_tDCS
**Cd320**	0.068089	8.12E-01	0.00E+00	27.48399	28.418305	26.63487	28.37417	29.226	27.34787	30.42414	Up_tDCS
**Cd38**	0.171802	7.49E-01	0.00E+00	8.83414	7.8395325	6.905336	8.106906	10.07793	8.103072	9.127241	Up_tDCS
**Cd4**	0.167854	7.67E-01	0.00E+00	5.889427	7.8395325	7.891812	6.080179	9.070138	8.103072	9.127241	Up_tDCS
**Cd40**	0.43519	7.48E-01	0.00E+00	0.981571	1.9598831	0.986477	1.013363	2.015586	2.025768	2.028276	Up_tDCS
**Cd55**	0.212507	8.51E-01	0.00E+00	1.963142	1.9598831	1.972953	2.026726	2.015586	3.038652	2.028276	Up_tDCS
**Cd84**	0.45832	6.27E-01	0.00E+00	0.981571	4.8997078	2.95943	3.04009	3.023379	3.038652	7.098965	Up_tDCS
**Cdh10**	-0.10028	7.47E-01	0.00E+00	23.55771	24.498539	27.62134	26.34744	23.17924	23.29633	21.29689	Down_tDCS
**Cdh12**	-0.49882	4.31E-01	0.00E+00	5.889427	5.8796494	11.83772	6.080179	5.038966	6.077304	5.070689	Down_tDCS
**Cdh13**	0.013875	9.49E-01	0.00E+00	43.18913	47.037195	47.35087	49.6548	45.35069	45.57978	44.62207	Up_tDCS
**Cdh15**	0.06842	9.26E-01	0.00E+00	4.907856	4.8997078	3.945906	5.066816	4.031172	5.06442	5.070689	Up_tDCS
**Cdh2**	-0.02762	9.01E-01	0.00E+00	45.15227	45.077312	44.39144	44.58798	44.3429	44.5669	42.59379	Down_tDCS
**Cdh3**	-0.11063	9.36E-01	0.00E+00	0.981571	0.9799416	2.95943	1.013363	1.007793	3.038652	1.014138	Down_tDCS
**Cdh5**	0.182162	6.90E-01	0.00E+00	9.815711	11.759299	9.864765	12.16036	12.09352	11.14172	12.16965	Up_tDCS
**Cdh7**	-0.24718	5.63E-01	0.00E+00	12.76042	12.73924	17.75658	13.17372	10.07793	13.16749	12.16965	Down_tDCS
**Cdh9**	-0.31638	5.88E-01	0.00E+00	6.870998	6.8595909	10.85124	7.093543	6.046759	7.090188	6.084827	Down_tDCS
**Cdk5**	0.115329	4.55E-01	0.00E+00	83.43355	90.154624	82.86403	93.22942	90.70138	94.19821	92.28654	Up_tDCS

**Cdk5r1**	-0.09584	5.79E-01	0.00E+00	72.63626	73.495617	76.94517	76.00224	69.53772	68.87611	63.89068	Down_tDCS
**Ceacam1**	-0.37193	8.10E-01	0.00E+00	0.981571	1.9598831	0.986477	1.013363	1.007793	1.012884	1.014138	Down_tDCS
**Celsr2**	-0.19736	3.25E-01	0.00E+00	55.94955	54.876728	64.12097	52.69489	51.39745	50.6442	48.67862	Down_tDCS
**Cgref1**	0.009047	9.71E-01	0.00E+00	33.37342	39.197663	34.52668	35.46771	38.29614	34.43806	35.49482	Up_tDCS
**Chrna4**	-0.13247	5.60E-01	0.00E+00	44.1707	43.117429	48.33735	41.54789	43.3351	40.51536	39.55138	Down_tDCS
**Chrna7**	-0.37336	6.26E-01	0.00E+00	3.926285	3.9197663	7.891812	4.053453	4.031172	4.051536	4.056551	Down_tDCS
**Chrnb2**	-0.17163	5.16E-01	0.00E+00	34.35499	32.338072	38.47258	32.42762	31.24159	30.38652	30.42414	Down_tDCS
**Cib2**	-0.03302	9.03E-01	0.00E+00	29.44713	33.318013	33.5402	28.37417	29.226	35.45094	32.45241	Down_tDCS
**Clic2**	-0.37193	8.10E-01	0.00E+00	0.981571	1.9598831	0.986477	1.013363	1.007793	1.012884	1.014138	Down_tDCS
**Clic4**	0.011758	9.55E-01	0.00E+00	53.98641	50.956961	46.3644	49.6548	55.42862	45.57978	52.73517	Up_tDCS
**Cln3**	-0.09791	7.76E-01	0.00E+00	20.61299	20.578773	19.72953	18.24054	20.15586	17.21903	20.28276	Down_tDCS
**Clstn2**	-0.42062	1.70E-01	0.00E+00	27.48399	26.458422	36.49963	24.32072	21.16366	21.27056	23.32517	Down_tDCS
**Cmklr1**	0.042506	9.71E-01	0.00E+00	1.963142	1.9598831	1.972953	2.026726	2.015586	2.025768	2.028276	Up_tDCS
**Cnn3**	0.186427	2.50E-01	0.00E+00	82.45198	85.254916	69.05336	91.20269	87.678	94.19821	86.20172	Up_tDCS
**Cnr1**	-0.09951	6.84E-01	0.00E+00	40.24442	37.237779	40.44554	39.52117	34.26497	37.47671	35.49482	Down_tDCS
**Colec11**	-0.08263	9.33E-01	0.00E+00	3.926285	2.9398247	1.972953	3.04009	3.023379	2.025768	3.042414	Down_tDCS
**Coro1a**	0.144767	3.46E-01	0.00E+00	85.39669	92.114507	86.80994	92.21606	94.73255	107.3657	95.32896	Up_tDCS
**Cpne3**	-0.06993	8.88E-01	0.00E+00	9.815711	9.7994156	9.864765	9.120269	9.070138	9.115956	10.14138	Down_tDCS
**Cps1**	1.523394	4.75E-01	0.00E+00	0.981571	0	0	1.013363	1.007793	1.012884	1.014138	Up_tDCS
**Crb2**	0.042505	9.79E-01	0.00E+00	0.981571	0.9799416	0.986477	1.013363	1.007793	1.012884	1.014138	Up_tDCS
**Creld1**	0.053414	7.47E-01	0.00E+00	78.52569	77.415383	72.99926	81.06906	73.5689	81.03072	81.13103	Up_tDCS
**Crh**	-0.20255	6.81E-01	0.00E+00	9.815711	10.779357	10.85124	10.13363	8.062345	10.12884	8.113103	Down_tDCS
**Crhbp**	0.078315	7.80E-01	0.00E+00	27.48399	31.35813	29.5943	35.46771	26.20262	30.38652	32.45241	Up_tDCS
**Crhr1**	-0.0414	9.04E-01	0.00E+00	19.63142	20.578773	20.71601	20.26726	21.16366	17.21903	20.28276	Down_tDCS
**Crp**	0.042505	9.79E-01	0.00E+00	0.981571	0.9799416	0.986477	1.013363	1.007793	1.012884	1.014138	Up_tDCS
**Cth**	0.252116	6.32E-01	0.00E+00	8.83414	7.8395325	6.905336	9.120269	9.070138	10.12884	9.127241	Up_tDCS
**Ctnnb1**	0.086423	5.57E-01	0.00E+00	95.2124	94.07439	96.6747	103.3631	101.7871	101.2884	98.37137	Up_tDCS
**Cul5**	-0.20803	6.81E-01	0.00E+00	7.852569	8.8194741	15.78362	10.13363	8.062345	10.12884	9.127241	Down_tDCS
**Cx3cr1**	0.195972	4.90E-01	0.00E+00	27.48399	32.338072	25.64839	28.37417	32.24938	30.38652	39.55138	Up_tDCS
**Cxcl10**	2.850851	3.51E-02	0.00E+00	0.981571	0.9799416	0.986477	1.013363	1.007793	23.29633	3.042414	Up_tDCS
**Cxcl13**	1.845741	3.62E-01	0.00E+00	0	0	0.986477	1.013363	1.007793	1.012884	2.028276	Up_tDCS
**Cxcr4**	0.08719	9.31E-01	0.00E+00	2.944713	2.9398247	1.972953	2.026726	3.023379	3.038652	3.042414	Up_tDCS
**Cyba**	0.590024	1.30E-01	0.00E+00	12.76042	13.719182	11.83772	16.21381	20.15586	22.28345	18.25448	Up_tDCS
**D2hgdh**	-0.26351	5.01E-01	0.00E+00	16.68671	17.638948	15.78362	12.16036	14.1091	14.18038	15.21207	Down_tDCS
**Dag1**	-0.11276	5.98E-01	0.00E+00	50.06013	47.037195	52.28326	46.61471	47.36628	44.5669	45.6362	Down_tDCS
**Dapk1**	-0.26748	4.43E-01	0.00E+00	20.61299	18.61889	25.64839	19.2539	18.14028	17.21903	17.24034	Down_tDCS
**Dapk2**	0.259622	6.55E-01	0.00E+00	6.870998	5.8796494	6.905336	8.106906	8.062345	7.090188	8.113103	Up_tDCS
**Dchs1**	-0.05093	9.61E-01	0.00E+00	1.963142	2.9398247	2.95943	1.013363	3.023379	3.038652	3.042414	Down_tDCS
**Ddit3**	0.036202	8.53E-01	0.00E+00	53.00484	59.776435	55.24269	52.69489	57.44421	60.77304	58.82	Up_tDCS
**Dgkb**	-0.46817	2.79E-01	0.00E+00	12.76042	13.719182	19.72953	10.13363	12.09352	10.12884	12.16965	Down_tDCS
**Dkk1**	0.042505	9.79E-01	0.00E+00	0.981571	0.9799416	0.986477	1.013363	1.007793	1.012884	1.014138	Up_tDCS
**Dlk1**	-0.08354	9.32E-01	0.00E+00	1.963142	2.9398247	3.945906	3.04009	3.023379	2.025768	3.042414	Down_tDCS
**Dll1**	-0.13789	8.41E-01	0.00E+00	5.889427	5.8796494	4.932383	5.066816	5.038966	5.06442	5.070689	Down_tDCS
**Dll4**	0.043186	9.53E-01	0.00E+00	4.907856	6.8595909	2.95943	4.053453	5.038966	6.077304	5.070689	Up_tDCS
**Drd2**	-0.29467	6.70E-01	0.00E+00	5.889427	4.8997078	6.905336	5.066816	4.031172	5.06442	5.070689	Down_tDCS
**Drd5**	-0.37308	8.39E-01	0.00E+00	0.981571	0.9799416	0.986477	1.013363	1.007793	1.012884	0	Down_tDCS
**Dusp1**	0.228386	2.27E-01	0.00E+00	63.80212	62.71626	52.28326	69.92206	65.50655	81.03072	62.87655	Up_tDCS
**Dyrk2**	-0.05062	9.21E-01	0.00E+00	8.83414	8.8194741	9.864765	9.120269	8.062345	9.115956	9.127241	Down_tDCS
**Dysf**	0.213722	9.20E-01	0.00E+00	0.981571	0.9799416	0	0	1.007793	1.012884	1.014138	Up_tDCS
**Edem1**	-0.08732	8.56E-01	0.00E+00	9.815711	9.7994156	11.83772	10.13363	10.07793	9.115956	10.14138	Down_tDCS
**Edem2**	-0.02608	9.41E-01	0.00E+00	21.59456	19.598831	16.7701	19.2539	17.13248	18.23191	21.29689	Down_tDCS
**Edn1**	-0.03139	9.67E-01	0.00E+00	2.944713	6.8595909	4.932383	3.04009	5.038966	6.077304	5.070689	Down_tDCS
**Edn3**	0.075919	9.30E-01	0.00E+00	3.926285	4.8997078	1.972953	4.053453	3.023379	4.051536	4.056551	Up_tDCS
**Ednra**	-0.37153	7.91E-01	0.00E+00	1.963142	1.9598831	0.986477	1.013363	1.007793	1.012884	2.028276	Down_tDCS
**Ednrb**	0.074072	7.60E-01	0.00E+00	36.31813	38.217721	36.49963	38.5078	40.31172	38.48959	38.53724	Up_tDCS
**Efemp1**	-0.21899	8.48E-01	0.00E+00	2.944713	2.9398247	2.95943	2.026726	0	2.025768	6.084827	Down_tDCS
**Efemp2**	0.002525	9.94E-01	0.00E+00	20.61299	21.558714	20.71601	22.29399	21.16366	20.25768	20.28276	Up_tDCS
**Efhc1**	0.086387	9.33E-01	0.00E+00	1.963142	1.9598831	3.945906	2.026726	2.015586	4.051536	3.042414	Up_tDCS
**Efhd1**	0.050605	8.21E-01	0.00E+00	47.11541	45.077312	40.44554	44.58798	46.35848	42.54113	49.69275	Up_tDCS
**Egf**	0.211068	9.28E-01	0.00E+00	0.981571	0	0.986477	0	1.007793	0	2.028276	Up_tDCS
**Egfr**	-0.37288	7.16E-01	0.00E+00	2.944713	2.9398247	2.95943	2.026726	3.023379	2.025768	2.028276	Down_tDCS
**Ehd3**	0.005964	9.73E-01	0.00E+00	63.80212	65.656085	67.0804	65.86861	65.50655	64.82458	66.9331	Up_tDCS
**Eif2ak3**	-0.12468	7.51E-01	0.00E+00	15.70514	14.699123	16.7701	14.18709	14.1091	14.18038	15.21207	Down_tDCS
**Enpp1**	-0.17967	8.17E-01	0.00E+00	4.907856	4.8997078	3.945906	4.053453	4.031172	4.051536	4.056551	Down_tDCS
**Entpd6**	0.016177	9.38E-01	0.00E+00	50.06013	49.97702	49.32383	49.6548	50.38966	50.6442	50.70689	Up_tDCS
**Epdr1**	0.082027	6.13E-01	0.00E+00	76.56255	79.375267	82.86403	88.1626	79.61566	90.14668	79.10275	Up_tDCS
**Erbb3**	-0.29881	4.41E-01	0.00E+00	21.59456	16.659007	17.75658	12.16036	20.15586	12.15461	16.22621	Down_tDCS
**Ewsr1**	0.009873	9.55E-01	0.00E+00	73.61784	71.535734	74.97222	71.94879	78.60786	62.79881	82.14517	Up_tDCS
**Ezr**	0.099592	6.24E-01	0.00E+00	51.0417	50.956961	50.3103	57.76171	51.39745	55.70862	52.73517	Up_tDCS
**F10**	0.85034	5.62E-01	0.00E+00	0.981571	0.9799416	0.986477	1.013363	1.007793	3.038652	2.028276	Up_tDCS

**F12**	-0.03519	9.56E-01	0.00E+00	6.870998	6.8595909	4.932383	6.080179	6.046759	6.077304	6.084827	Down_tDCS
**F2r**	-0.17101	6.32E-01	0.00E+00	18.64985	20.578773	19.72953	16.21381	20.15586	15.19326	18.25448	Down_tDCS
**F2rl1**	-0.20213	8.49E-01	0.00E+00	2.944713	2.9398247	1.972953	2.026726	2.015586	3.038652	2.028276	Down_tDCS
**Fam20a**	-0.14984	8.24E-01	0.00E+00	4.907856	6.8595909	5.918859	6.080179	4.031172	6.077304	5.070689	Down_tDCS
**Fam20c**	0.01002	9.68E-01	0.00E+00	37.2997	38.217721	34.52668	36.48108	37.28835	35.45094	38.53724	Up_tDCS
**Fas**	0.042505	9.79E-01	0.00E+00	0.981571	0.9799416	0.986477	1.013363	1.007793	1.012884	1.014138	Up_tDCS
**Fat1**	-0.05081	9.31E-01	0.00E+00	6.870998	8.8194741	7.891812	6.080179	10.07793	4.051536	10.14138	Down_tDCS
**Fat3**	-0.10981	9.05E-01	0.00E+00	2.944713	2.9398247	3.945906	3.04009	3.023379	3.038652	3.042414	Down_tDCS
**Fat4**	0.042506	9.71E-01	0.00E+00	1.963142	1.9598831	1.972953	2.026726	2.015586	2.025768	2.028276	Up_tDCS
**Fbln1**	0.042675	8.89E-01	0.00E+00	24.53928	26.458422	22.68896	26.34744	24.18703	24.30922	26.36758	Up_tDCS
**Fbln2**	-1.09546	7.16E-01	0.00E+00	0.981571	0	0.986477	0	1.007793	0	0	Down_tDCS
**Fbn1**	-0.10957	9.35E-01	0.00E+00	1.963142	0.9799416	1.972953	1.013363	1.007793	2.025768	2.028276	Down_tDCS
**Fgf1**	-0.37337	8.09E-01	0.00E+00	0.981571	0.9799416	1.972953	1.013363	1.007793	1.012884	1.014138	Down_tDCS
**Fgf14**	-0.20724	6.45E-01	0.00E+00	10.79728	10.779357	18.74305	12.16036	13.10131	10.12884	11.15552	Down_tDCS
**Fgf2**	-0.08327	8.76E-01	0.00E+00	8.83414	8.8194741	8.878289	9.120269	10.07793	8.103072	6.084827	Down_tDCS
**Fhl2**	-0.02969	8.83E-01	0.00E+00	51.0417	54.876728	55.24269	53.70825	53.41304	54.69574	48.67862	Down_tDCS
**Fkbp10**	0.15808	8.01E-01	0.00E+00	5.889427	5.8796494	5.918859	7.093543	6.046759	6.077304	7.098965	Up_tDCS
**Fkbp14**	0.058132	9.17E-01	0.00E+00	7.852569	6.8595909	7.891812	7.093543	7.054552	8.103072	9.127241	Up_tDCS
**Fos**	0.635403	1.18E-02	0.00E+00	30.42871	29.398247	31.56725	48.64144	37.28835	48.61843	54.76344	Up_tDCS
**Fstl1**	-0.05741	8.43E-01	0.00E+00	29.44713	26.458422	28.60782	26.34744	28.21821	27.34787	26.36758	Down_tDCS
**Fus**	0.139508	3.46E-01	0.00E+00	95.2124	91.134565	92.72879	107.4165	100.7793	102.3013	99.38551	Up_tDCS
**Fxyd5**	0.237646	4.23E-01	0.00E+00	22.57614	27.438364	24.66191	26.34744	27.21041	32.41229	31.43827	Up_tDCS
**Fyn**	0.063624	6.86E-01	0.00E+00	83.43355	86.234858	80.89108	85.12251	86.67021	86.09514	91.27241	Up_tDCS
**Fzd2**	-0.05831	8.96E-01	0.00E+00	13.742	12.73924	9.864765	12.16036	13.10131	11.14172	10.14138	Down_tDCS
**Fzd4**	-0.12483	8.62E-01	0.00E+00	5.889427	4.8997078	4.932383	5.066816	6.046759	4.051536	4.056551	Down_tDCS
**Fzd6**	-0.17956	8.73E-01	0.00E+00	1.963142	2.9398247	1.972953	2.026726	2.015586	2.025768	2.028276	Down_tDCS
**G6pd**	0.084246	6.37E-01	0.00E+00	63.80212	65.656085	63.1345	67.89534	68.52993	65.83746	69.97551	Up_tDCS
**Gal**	-0.28404	7.20E-01	0.00E+00	8.83414	4.8997078	1.972953	5.066816	4.031172	3.038652	5.070689	Down_tDCS
**Galr2**	-0.01002	9.87E-01	0.00E+00	6.870998	6.8595909	6.905336	7.093543	7.054552	7.090188	6.084827	Down_tDCS
**Gas6**	0.062931	6.75E-01	0.00E+00	92.26769	92.114507	91.74232	96.26951	92.71697	99.26263	96.3431	Up_tDCS
**Gata2**	0.168422	8.40E-01	0.00E+00	2.944713	4.8997078	2.95943	4.053453	4.031172	4.051536	4.056551	Up_tDCS
**Gch1**	0.627051	7.38E-01	0.00E+00	0	0.9799416	0.986477	1.013363	1.007793	1.012884	1.014138	Up_tDCS
**Ghrh**	1.523394	4.75E-01	0.00E+00	0	0.9799416	0	1.013363	1.007793	1.012884	1.014138	Up_tDCS
**Ghrl**	-0.83205	4.07E-01	0.00E+00	2.944713	3.9197663	3.945906	2.026726	2.015586	2.025768	2.028276	Down_tDCS
**Gipr**	0.255352	7.62E-01	0.00E+00	4.907856	2.9398247	2.95943	5.066816	5.038966	5.06442	2.028276	Up_tDCS
**Gja4**	0.141703	8.48E-01	0.00E+00	3.926285	4.8997078	4.932383	6.080179	6.046759	5.06442	3.042414	Up_tDCS
**Gjc2**	-0.06448	7.51E-01	0.00E+00	70.67312	62.71626	48.33735	56.74834	61.47538	50.6442	62.87655	Down_tDCS
**Glrb**	0.025372	8.69E-01	0.00E+00	90.30454	83.295033	98.64765	92.21606	90.70138	94.19821	92.28654	Up_tDCS
**Gna13**	-0.00588	9.89E-01	0.00E+00	13.742	10.779357	12.82419	11.147	13.10131	12.15461	13.18379	Down_tDCS
**Gnptab**	-0.15033	6.57E-01	0.00E+00	20.61299	19.598831	24.66191	20.26726	19.14807	19.2448	19.26862	Down_tDCS
**Gpd2**	-0.2093	5.95E-01	0.00E+00	15.70514	15.679065	17.75658	12.16036	16.12469	15.19326	13.18379	Down_tDCS
**Gpr17**	-0.08493	7.24E-01	0.00E+00	45.15227	44.09737	35.51316	40.53453	41.31952	34.43806	40.56551	Down_tDCS
**Grin2a**	0.141139	8.52E-01	0.00E+00	3.926285	2.9398247	6.905336	6.080179	5.038966	6.077304	3.042414	Up_tDCS
**Grin2b**	-0.46037	5.33E-01	0.00E+00	4.907856	4.8997078	6.905336	4.053453	4.031172	4.051536	4.056551	Down_tDCS
**Grin2d**	-0.21227	7.62E-01	0.00E+00	5.889427	4.8997078	5.918859	5.066816	5.038966	5.06442	4.056551	Down_tDCS
**Grm1**	-0.15024	7.42E-01	0.00E+00	11.77885	10.779357	12.82419	11.147	10.07793	11.14172	10.14138	Down_tDCS
**Grm2**	-0.01709	9.54E-01	0.00E+00	30.42871	30.378188	23.67544	33.44099	24.18703	28.36075	25.35345	Down_tDCS
**Grm3**	-0.07669	7.51E-01	0.00E+00	45.15227	37.237779	38.47258	36.48108	41.31952	36.46383	38.53724	Down_tDCS
**Grm5**	-0.33694	3.08E-01	0.00E+00	24.53928	20.578773	32.55373	22.29399	21.16366	20.25768	18.25448	Down_tDCS
**Grm6**	-0.37275	8.39E-01	0.00E+00	0.981571	0.9799416	0.986477	1.013363	1.007793	0	1.014138	Down_tDCS
**Grm7**	-0.10962	7.11E-01	0.00E+00	27.48399	25.478481	30.58077	27.36081	25.19483	24.30922	26.36758	Down_tDCS
**Gsk3b**	-0.20775	3.91E-01	0.00E+00	41.22599	37.237779	51.29678	39.52117	37.28835	36.46383	36.50896	Down_tDCS
**Gstm2**	0.019874	9.77E-01	0.00E+00	4.907856	4.8997078	5.918859	6.080179	4.031172	5.06442	6.084827	Up_tDCS
**Gsto1**	0.125414	4.16E-01	0.00E+00	85.39669	88.194741	83.85051	94.24278	97.75593	94.19821	88.22999	Up_tDCS
**Gtf2i**	0.094506	5.42E-01	0.00E+00	85.39669	86.234858	82.86403	88.1626	90.70138	91.15956	92.28654	Up_tDCS
**Hcrt**	-1.67533	6.01E-01	0.00E+00	0	2.9398247	0	0	1.007793	0	0	Down_tDCS
**Hcrtr1**	-0.51022	5.98E-01	0.00E+00	2.944713	4.8997078	2.95943	2.026726	4.031172	2.025768	2.028276	Down_tDCS
**Hcrtr2**	-0.11042	9.34E-01	0.00E+00	1.963142	0.9799416	1.972953	1.013363	2.015586	2.025768	1.014138	Down_tDCS
**Hdac4**	-0.53805	2.95E-01	0.00E+00	13.742	9.7994156	12.82419	10.13363	11.08572	4.051536	8.113103	Down_tDCS
**Herpud1**	0.03792	8.52E-01	0.00E+00	53.98641	50.956961	50.3103	54.72162	55.42862	50.6442	51.72103	Up_tDCS
**Homer2**	-0.1188	8.27E-01	0.00E+00	7.852569	8.8194741	8.878289	8.106906	8.062345	6.077304	9.127241	Down_tDCS
**Homer3**	-0.09219	7.78E-01	0.00E+00	24.53928	24.498539	19.72953	21.28063	21.16366	19.2448	24.33931	Down_tDCS
**Hpcal1**	0.049528	8.47E-01	0.00E+00	32.39185	35.277896	33.5402	36.48108	34.26497	35.45094	33.46655	Up_tDCS
**Hpgds**	0.042505	9.79E-01	0.00E+00	0.981571	0.9799416	0.986477	1.013363	1.007793	1.012884	1.014138	Up_tDCS
**Hrc**	0.364573	8.16E-01	0.00E+00	0.981571	0.9799416	0.986477	1.013363	1.007793	2.025768	1.014138	Up_tDCS
**Hspa2**	-0.02456	9.51E-01	0.00E+00	14.72357	14.699123	13.81067	15.20045	14.1091	14.18038	13.18379	Down_tDCS
**Htr1b**	-0.25742	6.63E-01	0.00E+00	7.852569	5.8796494	9.864765	8.106906	6.046759	6.077304	6.084827	Down_tDCS
**Htr2a**	-0.47613	3.97E-01	0.00E+00	8.83414	6.8595909	12.82419	8.106906	6.046759	7.090188	6.084827	Down_tDCS
**Htr2c**	-0.00462	9.92E-01	0.00E+00	9.815711	10.779357	9.864765	13.17372	9.070138	8.103072	10.14138	Down_tDCS
**Icam1**	0.212404	7.67E-01	0.00E+00	3.926285	4.8997078	4.932383	5.066816	5.038966	6.077304	5.070689	Up_tDCS

**Igf1**	-0.37212	7.37E-01	0.00E+00	1.963142	2.9398247	2.95943	3.04009	1.007793	2.025768	2.028276	Down_tDCS
**Ihh**	-0.37142	8.40E-01	0.00E+00	0.981571	0.9799416	0.986477	1.013363	0	1.012884	1.014138	Down_tDCS
**Il1rapl1**	-0.59442	6.29E-01	0.00E+00	2.944713	1.9598831	1.972953	1.013363	1.007793	2.025768	2.028276	Down_tDCS
**Immt**	0.015438	9.39E-01	0.00E+00	50.06013	51.936903	57.21564	53.70825	54.42083	52.66997	53.74931	Up_tDCS
**Iqcb1**	-0.00747	9.82E-01	0.00E+00	19.63142	23.518598	21.70248	22.29399	20.15586	24.30922	19.26862	Down_tDCS
**Iqgap1**	0.264394	6.72E-01	0.00E+00	6.870998	3.9197663	6.905336	7.093543	8.062345	7.090188	6.084827	Up_tDCS
**Itpkc**	-0.10952	7.99E-01	0.00E+00	11.77885	13.719182	13.81067	12.16036	12.09352	11.14172	13.18379	Down_tDCS
**Itpr1**	-0.22096	3.07E-01	0.00E+00	53.00484	47.037195	68.06688	49.6548	52.40524	46.59267	43.60793	Down_tDCS
**Itpr3**	0.627942	6.02E-01	0.00E+00	0.981571	2.9398247	0.986477	3.04009	3.023379	3.038652	1.014138	Up_tDCS
**Itsn1**	-0.46159	2.01E-01	0.00E+00	20.61299	20.578773	24.66191	16.21381	16.12469	19.2448	12.16965	Down_tDCS
**Jag1**	-0.17975	8.73E-01	0.00E+00	2.944713	1.9598831	1.972953	2.026726	2.015586	2.025768	2.028276	Down_tDCS
**Jag2**	0.052862	9.06E-01	0.00E+00	12.76042	10.779357	10.85124	12.16036	11.08572	13.16749	11.15552	Up_tDCS
**Jak2**	-0.20911	5.96E-01	0.00E+00	15.70514	14.699123	18.74305	14.18709	12.09352	15.19326	15.21207	Down_tDCS
**Jph1**	-0.30269	6.46E-01	0.00E+00	6.870998	4.8997078	7.891812	6.080179	6.046759	5.06442	4.056551	Down_tDCS
**Jsrp1**	0.042505	9.79E-01	0.00E+00	0.981571	0.9799416	0.986477	1.013363	1.007793	1.012884	1.014138	Up_tDCS
**Jun**	0.053903	7.51E-01	0.00E+00	77.54412	76.435442	66.09393	78.02897	71.55331	79.00495	76.06034	Up_tDCS
**Junb**	0.311434	1.20E-01	0.00E+00	55.94955	47.037195	49.32383	66.88197	56.43641	69.889	58.82	Up_tDCS
**Kcna5**	0.042506	9.71E-01	0.00E+00	1.963142	1.9598831	1.972953	2.026726	2.015586	2.025768	2.028276	Up_tDCS
**Kcne2**	-0.42496	5.72E-01	0.00E+00	5.889427	3.9197663	18.74305	5.066816	4.031172	16.20614	3.042414	Down_tDCS
**Kcnk3**	-0.22325	5.49E-01	0.00E+00	17.66828	17.638948	18.74305	15.20045	16.12469	15.19326	15.21207	Down_tDCS
**Kcnma1**	-0.1478	6.42E-01	0.00E+00	23.55771	21.558714	30.58077	23.30735	23.17924	21.27056	23.32517	Down_tDCS
**Kcnn2**	-0.07399	7.97E-01	0.00E+00	28.46556	28.418305	28.60782	27.36081	26.20262	28.36075	26.36758	Down_tDCS
**Kcnn3**	-0.23508	8.04E-01	0.00E+00	3.926285	2.9398247	2.95943	3.04009	3.023379	3.038652	2.028276	Down_tDCS
**Kcnn4**	0.364573	8.16E-01	0.00E+00	0.981571	0.9799416	0.986477	1.013363	1.007793	2.025768	1.014138	Up_tDCS
**Kcnq3**	-0.43074	3.00E-01	0.00E+00	15.70514	12.73924	21.70248	14.18709	12.09352	12.15461	11.15552	Down_tDCS
**Kcnq4**	-0.37195	6.61E-01	0.00E+00	4.907856	4.8997078	2.95943	4.053453	3.023379	3.038652	3.042414	Down_tDCS
**Kctd17**	0.055917	7.47E-01	0.00E+00	72.63626	76.435442	64.12097	80.0557	70.54552	70.90188	74.03206	Up_tDCS
**Kl**	-0.05405	9.68E-01	0.00E+00	0.981571	0	10.85124	1.013363	1.007793	12.15461	1.014138	Down_tDCS
**Lat**	-0.17963	8.75E-01	0.00E+00	1.963142	2.9398247	1.972953	3.04009	2.015586	2.025768	1.014138	Down_tDCS
**Lck**	0.212507	8.51E-01	0.00E+00	1.963142	1.9598831	1.972953	2.026726	2.015586	3.038652	2.028276	Up_tDCS
**Lcp1**	0.355375	4.30E-01	0.00E+00	10.79728	9.7994156	10.85124	10.13363	13.10131	14.18038	16.22621	Up_tDCS
**Ldlr**	-0.106	7.48E-01	0.00E+00	24.53928	20.578773	22.68896	23.30735	20.15586	18.23191	22.31103	Down_tDCS
**Lef1**	-0.0099	9.89E-01	0.00E+00	3.926285	4.8997078	4.932383	4.053453	4.031172	5.06442	5.070689	Down_tDCS
**Letm1**	-0.08655	6.82E-01	0.00E+00	51.0417	47.037195	54.25621	48.64144	49.38186	47.60555	45.6362	Down_tDCS
**Lgals3**	-0.01918	9.77E-01	0.00E+00	3.926285	5.8796494	7.891812	6.080179	5.038966	6.077304	6.084827	Down_tDCS
**Lgmn**	0.187628	2.47E-01	0.00E+00	75.58098	81.33515	72.99926	81.06906	87.678	88.12091	92.28654	Up_tDCS
**Lmcd1**	0.162697	6.63E-01	0.00E+00	15.70514	18.61889	13.81067	18.24054	18.14028	19.2448	16.22621	Up_tDCS
**Loxl2**	-0.78743	6.48E-01	0.00E+00	0.981571	0.9799416	1.972953	1.013363	0	1.012884	1.014138	Down_tDCS
**Lpar1**	-0.04276	8.50E-01	0.00E+00	59.87584	50.956961	38.47258	48.64144	50.38966	42.54113	51.72103	Down_tDCS
**Lpar6**	-0.26568	7.47E-01	0.00E+00	3.926285	3.9197663	4.932383	4.053453	3.023379	3.038652	4.056551	Down_tDCS
**Lrp1**	0.021717	9.20E-01	0.00E+00	45.15227	46.057253	46.3644	44.58798	47.36628	48.61843	45.6362	Up_tDCS
**Lrp1b**	-0.07884	8.71E-01	0.00E+00	10.79728	9.7994156	9.864765	9.120269	10.07793	10.12884	9.127241	Down_tDCS
**Lrp4**	-0.0155	9.65E-01	0.00E+00	19.63142	19.598831	16.7701	17.22718	19.14807	19.2448	18.25448	Down_tDCS
**Lrp6**	-0.2416	7.00E-01	0.00E+00	6.870998	5.8796494	7.891812	6.080179	6.046759	6.077304	5.070689	Down_tDCS
**Lrp8**	-0.10373	8.14E-01	0.00E+00	11.77885	11.759299	14.79715	12.16036	14.1091	9.115956	12.16965	Down_tDCS
**Ltbp2**	0.627051	7.38E-01	0.00E+00	0	0.9799416	0.986477	1.013363	1.007793	1.012884	1.014138	Up_tDCS
**Ltbp3**	-0.17751	5.73E-01	0.00E+00	25.52085	24.498539	24.66191	23.30735	22.17145	20.25768	22.31103	Down_tDCS
**Lyn**	0.386655	4.32E-01	0.00E+00	8.83414	8.8194741	7.891812	10.13363	10.07793	12.15461	12.16965	Up_tDCS
**Man1b1**	0.056327	7.82E-01	0.00E+00	52.02327	50.956961	50.3103	53.70825	53.41304	51.65709	53.74931	Up_tDCS
**Map2**	-0.07967	6.34E-01	0.00E+00	80.48883	73.495617	94.70175	78.02897	83.64683	75.9663	76.06034	Down_tDCS
**Map3k7**	-0.23728	5.08E-01	0.00E+00	18.64985	19.598831	21.70248	18.24054	17.13248	14.18038	18.25448	Down_tDCS
**Map6**	0.035711	8.37E-01	0.00E+00	69.69155	69.575851	68.06688	73.97552	67.52214	70.90188	70.98965	Up_tDCS
**Mapk7**	0.003155	9.94E-01	0.00E+00	11.77885	13.719182	10.85124	12.16036	12.09352	12.15461	12.16965	Up_tDCS
**Mapkapk3**	0.112987	7.85E-01	0.00E+00	12.76042	14.699123	11.83772	13.17372	15.1169	14.18038	14.19793	Up_tDCS
**Marcks**	-0.09039	6.90E-01	0.00E+00	50.06013	46.057253	40.44554	44.58798	39.30393	43.55401	43.60793	Down_tDCS
**Masp1**	-0.0561	8.36E-01	0.00E+00	35.33656	34.297955	30.58077	36.48108	29.226	35.45094	27.38172	Down_tDCS
**Masp2**	0.079737	8.98E-01	0.00E+00	5.889427	5.8796494	6.905336	6.080179	7.054552	7.090188	6.084827	Up_tDCS
**Matn4**	0.095733	8.06E-01	0.00E+00	16.68671	17.638948	11.83772	16.21381	14.1091	17.21903	18.25448	Up_tDCS
**Mcfd2**	0.087936	6.91E-01	0.00E+00	43.18913	45.077312	42.41849	45.60135	45.35069	49.63132	44.62207	Up_tDCS
**Mcoln1**	-0.04572	8.13E-01	0.00E+00	57.9127	57.816552	58.20212	55.73498	56.43641	54.69574	57.80586	Down_tDCS
**Mef2a**	-0.37292	3.04E-01	0.00E+00	23.55771	17.638948	26.63487	19.2539	17.13248	21.27056	12.16965	Down_tDCS
**Mef2c**	-0.11535	4.93E-01	0.00E+00	83.43355	73.495617	94.70175	83.09579	77.60007	77.99207	70.98965	Down_tDCS
**Megf8**	0.068161	8.70E-01	0.00E+00	14.72357	11.759299	14.79715	16.21381	11.08572	13.16749	17.24034	Up_tDCS
**Mgmt**	-0.09669	8.82E-01	0.00E+00	4.907856	6.8595909	6.905336	6.080179	4.031172	7.090188	6.084827	Down_tDCS
**Mgp**	0.274923	2.46E-01	0.00E+00	34.35499	38.217721	38.47258	44.58798	41.31952	50.6442	42.59379	Up_tDCS
**Mip**	0.101544	8.73E-01	0.00E+00	5.889427	6.8595909	4.932383	6.080179	7.054552	6.077304	6.084827	Up_tDCS
**Mknk1**	-0.02452	9.47E-01	0.00E+00	14.72357	20.578773	18.74305	18.24054	16.12469	19.2448	17.24034	Down_tDCS
**Mtor**	-0.10682	7.19E-01	0.00E+00	26.50242	25.478481	30.58077	25.33408	26.20262	25.3221	25.35345	Down_tDCS
**Myh10**	-0.10146	6.49E-01	0.00E+00	47.11541	42.137487	53.26973	46.61471	46.35848	43.55401	40.56551	Down_tDCS
**Myh14**	-0.03168	9.66E-01	0.00E+00	6.870998	2.9398247	4.932383	4.053453	5.038966	4.051536	6.084827	Down_tDCS

**Myh2**	0.023518	9.57E-01	0.00E+00	10.79728	13.719182	12.82419	12.16036	11.08572	14.18038	13.18379	Up_tDCS
**Myh6**	-0.05044	9.61E-01	0.00E+00	2.944713	2.9398247	1.972953	3.04009	3.023379	2.025768	2.028276	Down_tDCS
**Myh7**	0.042505	9.79E-01	0.00E+00	0.981571	0.9799416	0.986477	1.013363	1.007793	1.012884	1.014138	Up_tDCS
**Myh9**	0.393158	2.66E-01	0.00E+00	14.72357	18.61889	15.78362	21.28063	20.15586	22.28345	22.31103	Up_tDCS
**Myl1**	0.158792	8.67E-01	0.00E+00	2.944713	3.9197663	1.972953	3.04009	3.023379	2.025768	5.070689	Up_tDCS
**Myl9**	0.12924	6.41E-01	0.00E+00	29.44713	32.338072	25.64839	33.44099	30.23379	32.41229	31.43827	Up_tDCS
**Myo10**	0.034713	9.29E-01	0.00E+00	15.70514	15.679065	13.81067	15.20045	16.12469	15.19326	15.21207	Up_tDCS
**Myo1b**	-0.2388	5.92E-01	0.00E+00	11.77885	10.779357	17.75658	11.147	12.09352	12.15461	10.14138	Down_tDCS
**Myo1c**	0.122551	8.36E-01	0.00E+00	3.926285	9.7994156	7.891812	9.120269	6.046759	7.090188	9.127241	Up_tDCS
**Myo1d**	-0.03258	9.23E-01	0.00E+00	23.55771	20.578773	18.74305	19.2539	21.16366	19.2448	22.31103	Down_tDCS
**Myo1f**	0.627523	5.52E-01	0.00E+00	1.963142	1.9598831	1.972953	2.026726	3.023379	3.038652	4.056551	Up_tDCS
**Myo5b**	-0.37248	6.09E-01	0.00E+00	4.907856	5.8796494	5.918859	5.066816	4.031172	3.038652	5.070689	Down_tDCS
**Myo6**	-0.13551	6.56E-01	0.00E+00	32.39185	25.478481	24.66191	25.33408	28.21821	22.28345	24.33931	Down_tDCS
**Myo9b**	-0.02786	9.34E-01	0.00E+00	22.57614	20.578773	18.74305	19.2539	22.17145	19.2448	20.28276	Down_tDCS
**Ncan**	-0.10949	6.46E-01	0.00E+00	41.22599	40.177604	41.43201	39.52117	37.28835	35.45094	39.55138	Down_tDCS
**Nedd4**	0.050542	7.43E-01	0.00E+00	87.35983	85.254916	89.76936	91.20269	91.70917	90.14668	89.24413	Up_tDCS
**Nell1**	-0.03886	8.59E-01	0.00E+00	49.07856	45.077312	49.32383	41.54789	48.37407	43.55401	52.73517	Down_tDCS
**Nfatc2**	-0.16636	8.37E-01	0.00E+00	3.926285	3.9197663	4.932383	4.053453	4.031172	3.038652	4.056551	Down_tDCS
**Ngfr**	0.112294	9.30E-01	0.00E+00	1.963142	0.9799416	1.972953	1.013363	2.015586	2.025768	2.028276	Up_tDCS
**Nid1**	-0.28493	7.02E-01	0.00E+00	5.889427	4.8997078	4.932383	6.080179	4.031172	3.038652	4.056551	Down_tDCS
**Nid2**	0.014651	9.85E-01	0.00E+00	4.907856	3.9197663	3.945906	4.053453	4.031172	4.051536	5.070689	Up_tDCS
**Nlgn1**	-0.19227	5.13E-01	0.00E+00	27.48399	26.458422	34.52668	26.34744	27.21041	25.3221	24.33931	Down_tDCS
**Nol3**	0.053392	8.70E-01	0.00E+00	20.61299	24.498539	20.71601	22.29399	21.16366	26.33498	21.29689	Up_tDCS
**Nos1**	-0.29056	6.85E-01	0.00E+00	4.907856	4.8997078	6.905336	5.066816	5.038966	4.051536	4.056551	Down_tDCS
**Nos3**	0.071287	8.95E-01	0.00E+00	8.83414	9.7994156	5.918859	8.106906	10.07793	9.115956	7.098965	Up_tDCS
**Notch1**	-0.62449	3.21E-01	0.00E+00	10.79728	5.8796494	7.891812	5.066816	7.054552	5.06442	4.056551	Down_tDCS
**Notch2**	-0.37248	6.18E-01	0.00E+00	5.889427	4.8997078	4.932383	4.053453	4.031172	4.051536	4.056551	Down_tDCS
**Notch3**	0.125074	8.50E-01	0.00E+00	5.889427	5.8796494	4.932383	7.093543	6.046759	7.090188	4.056551	Up_tDCS
**Notch4**	-0.0113	9.93E-01	0.00E+00	2.944713	0.9799416	2.95943	4.053453	3.023379	2.025768	0	Down_tDCS
**Npsr1**	0.042506	9.64E-01	0.00E+00	2.944713	2.9398247	2.95943	3.04009	3.023379	3.038652	3.042414	Up_tDCS
**Nrg1**	-0.47975	5.61E-01	0.00E+00	3.926285	4.8997078	4.932383	3.04009	4.031172	3.038652	3.042414	Down_tDCS
**Nrxn2**	0.00811	9.56E-01	0.00E+00	101.1018	99.954039	101.6071	110.4566	102.7949	99.26263	93.30068	Up_tDCS
**Ntsr1**	0.042506	9.71E-01	0.00E+00	1.963142	1.9598831	1.972953	2.026726	2.015586	2.025768	2.028276	Up_tDCS
**Nucb2**	0.237095	5.78E-01	0.00E+00	10.79728	9.7994156	16.7701	14.18709	14.1091	14.18038	16.22621	Up_tDCS
**Opa1**	-0.07119	7.72E-01	0.00E+00	34.35499	39.197663	53.26973	40.53453	38.29614	42.54113	39.55138	Down_tDCS
**Oprd1**	-0.25744	7.66E-01	0.00E+00	3.926285	2.9398247	4.932383	4.053453	3.023379	3.038652	3.042414	Down_tDCS
**Oprl1**	0.061207	8.61E-01	0.00E+00	19.63142	16.659007	19.72953	20.26726	20.15586	20.25768	17.24034	Up_tDCS
**Orai1**	0.187065	5.87E-01	0.00E+00	19.63142	20.578773	15.78362	22.29399	22.17145	20.25768	20.28276	Up_tDCS
**Otof**	-0.32967	5.07E-01	0.00E+00	9.815711	9.7994156	12.82419	8.106906	8.062345	9.115956	9.127241	Down_tDCS
**Oxt**	-0.37184	8.51E-01	0.00E+00	1.963142	0	0.986477	1.013363	0	1.012884	1.014138	Down_tDCS
**P2rx1**	0.042505	9.79E-01	0.00E+00	0.981571	0.9799416	0.986477	1.013363	1.007793	1.012884	1.014138	Up_tDCS
**P2rx2**	-2.09308	3.48E-01	0.00E+00	0.981571	1.9598831	0.986477	0	1.007793	0	0	Down_tDCS
**P2rx4**	-0.04624	8.88E-01	0.00E+00	24.53928	27.438364	17.75658	23.30735	24.18703	20.25768	22.31103	Down_tDCS
**P2rx7**	-0.12427	8.03E-01	0.00E+00	12.76042	11.759299	6.905336	10.13363	11.08572	8.103072	9.127241	Down_tDCS
**P2ry1**	0.042506	9.71E-01	0.00E+00	1.963142	1.9598831	1.972953	2.026726	2.015586	2.025768	2.028276	Up_tDCS
**P2ry12**	0.083531	7.98E-01	0.00E+00	26.50242	22.538656	19.72953	26.34744	20.15586	21.27056	29.41	Up_tDCS
**Pam**	0.106021	5.13E-01	0.00E+00	72.63626	83.295033	78.91812	83.09579	81.63124	87.10803	85.18758	Up_tDCS
**Pamr1**	0.082935	7.09E-01	0.00E+00	42.20756	44.09737	43.40497	47.62807	45.35069	44.5669	45.6362	Up_tDCS
**Pcdh11x**	-0.2136	7.80E-01	0.00E+00	4.907856	1.9598831	9.864765	6.080179	5.038966	4.051536	4.056551	Down_tDCS
**Pcdh15**	0.363727	8.17E-01	0.00E+00	0.981571	0.9799416	0.986477	1.013363	2.015586	1.012884	1.014138	Up_tDCS
**Pcdh19**	-0.48982	6.01E-01	0.00E+00	4.907856	0.9799416	6.905336	3.04009	4.031172	3.038652	2.028276	Down_tDCS
**Pcdh9**	-0.14447	5.27E-01	0.00E+00	46.13384	40.177604	51.29678	42.56126	42.32731	40.51536	40.56551	Down_tDCS
**Pcdhb8**	-0.17982	7.74E-01	0.00E+00	4.907856	7.8395325	7.891812	6.080179	5.038966	6.077304	7.098965	Down_tDCS
**Pclo**	-0.68156	2.83E-01	0.00E+00	7.852569	4.8997078	12.82419	6.080179	5.038966	5.06442	5.070689	Down_tDCS
**Pcyt1a**	-0.08761	7.62E-01	0.00E+00	28.46556	28.418305	28.60782	27.36081	28.21821	25.3221	26.36758	Down_tDCS
**Pdcd6ip**	-0.15995	5.44E-01	0.00E+00	35.33656	32.338072	37.48611	32.42762	32.24938	31.3994	29.41	Down_tDCS
**Pde1b**	-0.02375	8.89E-01	0.00E+00	69.69155	72.515676	76.94517	72.96215	72.5611	70.90188	70.98965	Down_tDCS
**Pde1c**	-0.37245	6.89E-01	0.00E+00	4.907856	2.9398247	2.95943	2.026726	3.023379	3.038652	3.042414	Down_tDCS
**Pde4b**	-0.08056	7.00E-01	0.00E+00	52.02327	48.997078	52.28326	50.66816	49.38186	45.57978	47.66448	Down_tDCS
**Pdgfb**	0.101498	7.42E-01	0.00E+00	24.53928	24.498539	21.70248	24.32072	25.19483	27.34787	24.33931	Up_tDCS
**Pdgfra**	0.065642	8.44E-01	0.00E+00	21.59456	20.578773	18.74305	20.26726	22.17145	21.27056	21.29689	Up_tDCS
**Pdgfrb**	-0.03805	9.23E-01	0.00E+00	14.72357	15.679065	14.79715	15.20045	14.1091	15.19326	14.19793	Down_tDCS
**Pdpk1**	-0.46369	1.59E-01	0.00E+00	26.50242	21.558714	32.55373	21.28063	20.15586	17.21903	19.26862	Down_tDCS
**Penk**	0.139874	3.31E-01	0.00E+00	99.13868	109.75346	97.66118	115.5234	104.8105	111.4172	118.6541	Up_tDCS
**Phka2**	0.522446	8.64E-01	0.00E+00	0	0	0.986477	1.013363	1.007793	0	0	Up_tDCS
**Phkb**	-0.55683	2.81E-01	0.00E+00	15.70514	10.779357	22.68896	14.18709	3.023379	11.14172	16.22621	Down_tDCS
**Phkg2**	0.062595	7.85E-01	0.00E+00	41.22599	41.157546	40.44554	41.54789	42.32731	42.54113	44.62207	Up_tDCS
**Pik3cb**	-0.09035	8.40E-01	0.00E+00	10.79728	11.759299	13.81067	11.147	12.09352	10.12884	12.16965	Down_tDCS
**Pitpnm1**	0.071539	6.32E-01	0.00E+00	91.28612	96.034273	90.75584	98.29623	99.77152	95.2111	96.3431	Up_tDCS
**Pitpnm3**	-0.08587	8.27E-01	0.00E+00	17.66828	14.699123	16.7701	18.24054	17.13248	15.19326	11.15552	Down_tDCS

**Pkd2**	-0.17142	7.89E-01	0.00E+00	5.889427	5.8796494	7.891812	4.053453	7.054552	6.077304	6.084827	Down_tDCS
**Pla2g12a**	0.033568	9.10E-01	0.00E+00	26.50242	27.438364	24.66191	28.37417	26.20262	26.33498	26.36758	Up_tDCS
**Pla2g2a**	0.042505	9.79E-01	0.00E+00	0.981571	0.9799416	0.986477	1.013363	1.007793	1.012884	1.014138	Up_tDCS
**Pla2g4a**	0.244122	7.24E-01	0.00E+00	4.907856	4.8997078	4.932383	4.053453	6.046759	6.077304	7.098965	Up_tDCS
**Pla2g5**	0.085228	9.42E-01	0.00E+00	0.981571	0.9799416	5.918859	2.026726	1.007793	6.077304	2.028276	Up_tDCS
**Pla2g6**	-0.1157	7.08E-01	0.00E+00	25.52085	26.458422	23.67544	23.30735	24.18703	22.28345	23.32517	Down_tDCS
**Plcb1**	-0.43313	2.46E-01	0.00E+00	17.66828	18.61889	36.49963	18.24054	18.14028	18.23191	17.24034	Down_tDCS
**Plcb4**	-0.26033	4.31E-01	0.00E+00	21.59456	21.558714	29.5943	21.28063	19.14807	20.25768	20.28276	Down_tDCS
**Plcd1**	0.021289	9.54E-01	0.00E+00	15.70514	18.61889	15.78362	17.22718	16.12469	17.21903	17.24034	Up_tDCS
**Plce1**	0.042505	9.79E-01	0.00E+00	0.981571	0.9799416	0.986477	1.013363	1.007793	1.012884	1.014138	Up_tDCS
**Plcg1**	-0.17721	4.96E-01	0.00E+00	33.37342	36.257838	45.37792	32.42762	38.29614	35.45094	29.41	Down_tDCS
**Plcg2**	0.43519	7.48E-01	0.00E+00	0.981571	1.9598831	0.986477	1.013363	2.015586	2.025768	2.028276	Up_tDCS
**Pln**	-0.18812	7.57E-01	0.00E+00	7.852569	5.8796494	7.891812	7.093543	5.038966	7.090188	6.084827	Down_tDCS
**Pls1**	-0.1422	7.07E-01	0.00E+00	17.66828	16.659007	16.7701	15.20045	15.1169	15.19326	16.22621	Down_tDCS
**Pls3**	0.04005	8.49E-01	0.00E+00	47.11541	48.017137	50.3103	48.64144	48.37407	51.65709	50.70689	Up_tDCS
**Plscr1**	0.210825	9.21E-01	0.00E+00	0.981571	0	0.986477	0	1.007793	1.012884	1.014138	Up_tDCS
**Plscr2**	0.042506	9.64E-01	0.00E+00	2.944713	2.9398247	2.95943	3.04009	3.023379	3.038652	3.042414	Up_tDCS
**Plscr4**	0.174169	8.21E-01	0.00E+00	3.926285	3.9197663	4.932383	3.04009	7.054552	5.06442	4.056551	Up_tDCS
**Pmpca**	0.002654	9.89E-01	0.00E+00	56.93113	52.916844	52.28326	56.74834	53.41304	52.66997	53.74931	Up_tDCS
**Ppif**	-0.07178	8.09E-01	0.00E+00	26.50242	28.418305	25.64839	25.33408	25.19483	25.3221	26.36758	Down_tDCS
**Ppp2r3a**	-0.07009	8.88E-01	0.00E+00	10.79728	8.8194741	9.864765	9.120269	10.07793	9.115956	9.127241	Down_tDCS
**Prkaa1**	-0.28788	5.62E-01	0.00E+00	10.79728	8.8194741	12.82419	8.106906	8.062345	10.12884	9.127241	Down_tDCS
**Prkaa2**	-0.08328	9.35E-01	0.00E+00	2.944713	1.9598831	3.945906	5.066816	2.015586	1.012884	3.042414	Down_tDCS
**Prkaca**	0.122952	4.04E-01	0.00E+00	94.23083	97.014215	91.74232	103.3631	98.76373	104.3271	104.4562	Up_tDCS
**Prkca**	-0.39082	2.29E-01	0.00E+00	26.50242	21.558714	31.56725	21.28063	21.16366	18.23191	20.28276	Down_tDCS
**Prkce**	-0.1332	4.55E-01	0.00E+00	69.69155	65.656085	76.94517	62.82852	65.50655	64.82458	64.90482	Down_tDCS
**Prkcg**	-0.00527	9.80E-01	0.00E+00	64.78369	53.896786	71.02631	77.01561	63.49097	64.82458	46.65034	Down_tDCS
**Prkcsh**	0.105063	4.77E-01	0.00E+00	93.24926	98.974098	90.75584	101.3363	100.7793	102.3013	101.4138	Up_tDCS
**Prkd1**	0.171705	7.49E-01	0.00E+00	6.870998	8.8194741	7.891812	8.106906	10.07793	8.103072	9.127241	Up_tDCS
**Prok2**	0.524818	8.63E-01	0.00E+00	0	0.9799416	0	1.013363	0	0	1.014138	Up_tDCS
**Psen1**	-0.08729	7.51E-01	0.00E+00	30.42871	29.398247	34.52668	30.4009	30.23379	28.36075	29.41	Down_tDCS
**Psen2**	-0.05421	8.60E-01	0.00E+00	27.48399	26.458422	21.70248	24.32072	25.19483	24.30922	23.32517	Down_tDCS
**Psph**	0.037446	9.06E-01	0.00E+00	22.57614	24.498539	21.70248	24.32072	23.17924	23.29633	23.32517	Up_tDCS
**Ptbp1**	0.172883	5.66E-01	0.00E+00	23.55771	24.498539	24.66191	24.32072	27.21041	30.38652	27.38172	Up_tDCS
**Ptgdr**	-0.19795	9.59E-01	0.00E+00	0	0	0.986477	0	0	0	1.014138	Down_tDCS
**Ptger1**	-0.2415	7.00E-01	0.00E+00	5.889427	6.8595909	7.891812	6.080179	6.046759	5.06442	6.084827	Down_tDCS
**Ptger3**	0.042505	9.79E-01	0.00E+00	0.981571	0.9799416	0.986477	1.013363	1.007793	1.012884	1.014138	Up_tDCS
**Ptges**	0.627016	5.90E-01	0.00E+00	0.981571	1.9598831	1.972953	2.026726	3.023379	3.038652	2.028276	Up_tDCS
**Ptgs2**	-0.09066	8.42E-01	0.00E+00	10.79728	9.7994156	15.78362	11.147	12.09352	12.15461	10.14138	Down_tDCS
**Pth1r**	0.006199	9.90E-01	0.00E+00	10.79728	10.779357	7.891812	10.13363	10.07793	11.14172	8.113103	Up_tDCS
**Ptk2b**	0.061088	6.73E-01	0.00E+00	97.17554	102.89386	102.5936	109.4432	107.8339	102.3013	101.4138	Up_tDCS
**Ptpn6**	0.235404	6.92E-01	0.00E+00	6.870998	7.8395325	5.918859	4.053453	8.062345	10.12884	10.14138	Up_tDCS
**Ptprc**	0.113368	9.29E-01	0.00E+00	1.963142	1.9598831	0.986477	1.013363	2.015586	2.025768	2.028276	Up_tDCS
**Rab32**	0.364676	7.41E-01	0.00E+00	1.963142	1.9598831	1.972953	2.026726	2.015586	3.038652	3.042414	Up_tDCS
**Ramp1**	0.083644	6.86E-01	0.00E+00	50.06013	57.816552	45.37792	56.74834	57.44421	51.65709	50.70689	Up_tDCS
**Ramp2**	0.037785	8.58E-01	0.00E+00	48.09699	53.896786	44.39144	51.68153	50.38966	49.63132	48.67862	Up_tDCS
**Rapgef2**	-0.25102	3.80E-01	0.00E+00	31.41028	27.438364	41.43201	27.36081	30.23379	29.37364	25.35345	Down_tDCS
**Rasgrp1**	-0.10735	5.05E-01	0.00E+00	96.19397	81.33515	114.4313	94.24278	89.69359	90.14668	87.21585	Down_tDCS
**Rasgrp2**	-0.01552	9.65E-01	0.00E+00	17.66828	19.598831	18.74305	18.24054	17.13248	20.25768	18.25448	Down_tDCS
**Rasgrp3**	0.0723	8.69E-01	0.00E+00	11.77885	11.759299	11.83772	12.16036	12.09352	11.14172	14.19793	Up_tDCS
**Rcan1**	0.078711	7.28E-01	0.00E+00	41.22599	44.09737	40.44554	42.56126	45.35069	45.57978	43.60793	Up_tDCS
**Rcn1**	0.057144	8.79E-01	0.00E+00	14.72357	16.659007	16.7701	17.22718	16.12469	17.21903	16.22621	Up_tDCS
**Rcn2**	0.126543	4.28E-01	0.00E+00	79.50726	82.315091	75.95869	89.17597	83.64683	86.09514	87.21585	Up_tDCS
**Rcn3**	0.062438	8.62E-01	0.00E+00	17.66828	18.61889	16.7701	18.24054	19.14807	19.2448	17.24034	Up_tDCS
**Reps2**	-0.57786	1.84E-01	0.00E+00	15.70514	11.759299	24.66191	11.147	14.1091	10.12884	11.15552	Down_tDCS
**Rest**	0.042506	9.71E-01	0.00E+00	1.963142	1.9598831	1.972953	2.026726	2.015586	2.025768	2.028276	Up_tDCS
**Rgs1**	0.950915	5.95E-01	0.00E+00	0.981571	0.9799416	0	1.013363	1.007793	2.025768	1.014138	Up_tDCS
**Rgs2**	0.118	5.49E-01	0.00E+00	51.0417	54.876728	54.25621	57.76171	54.42083	57.73439	61.86241	Up_tDCS
**Rgs9**	-0.09712	8.80E-01	0.00E+00	5.889427	5.8796494	6.905336	6.080179	6.046759	6.077304	5.070689	Down_tDCS
**Rhoa**	0.142114	4.06E-01	0.00E+00	72.63626	68.595909	65.10745	74.98888	74.57669	75.9663	78.08861	Up_tDCS
**Rims2**	-0.05393	8.48E-01	0.00E+00	28.46556	30.378188	32.55373	30.4009	33.25717	28.36075	25.35345	Down_tDCS
**Rit1**	-0.00553	9.79E-01	0.00E+00	48.09699	48.017137	46.3644	46.61471	48.37407	46.59267	47.66448	Down_tDCS
**Ror2**	-0.37229	8.10E-01	0.00E+00	1.963142	0.9799416	0.986477	1.013363	1.007793	1.012884	1.014138	Down_tDCS
**Rph3al**	0.338036	6.76E-01	0.00E+00	3.926285	3.9197663	2.95943	5.066816	5.038966	5.06442	3.042414	Up_tDCS
**Rrad**	-0.01874	9.77E-01	0.00E+00	6.870998	5.8796494	4.932383	7.093543	6.046759	5.06442	5.070689	Down_tDCS
**S100a1**	0.274207	9.08E-02	0.00E+00	82.45198	84.274974	66.09393	93.22942	92.71697	91.15956	98.37137	Up_tDCS
**S100a10**	0.079199	7.22E-01	0.00E+00	41.22599	47.037195	45.37792	46.61471	44.3429	53.68285	43.60793	Up_tDCS
**S100a11**	0.029728	9.54E-01	0.00E+00	8.83414	9.7994156	8.878289	9.120269	8.062345	12.15461	8.113103	Up_tDCS
**S100a4**	0.221347	5.53E-01	0.00E+00	21.59456	24.498539	11.83772	24.32072	21.16366	16.20614	28.39586	Up_tDCS
**S100a6**	-0.07828	8.35E-01	0.00E+00	15.70514	15.679065	20.71601	18.24054	15.1169	16.20614	16.22621	Down_tDCS

**S100a8**	0.434849	6.61E-01	0.00E+00	3.926285	1.9598831	1.972953	1.013363	4.031172	5.06442	4.056551	Up_tDCS
**S100a9**	1.005972	2.24E-01	0.00E+00	5.889427	1.9598831	1.972953	2.026726	8.062345	9.115956	7.098965	Up_tDCS
**S1pr1**	0.112093	5.12E-01	0.00E+00	75.58098	75.4555	63.1345	77.01561	76.59228	79.00495	76.06034	Up_tDCS
**Sdc1**	0.021804	9.61E-01	0.00E+00	11.77885	11.759299	10.85124	10.13363	12.09352	11.14172	13.18379	Up_tDCS
**Sdf4**	0.134699	3.88E-01	0.00E+00	85.39669	83.295033	80.89108	88.1626	88.68579	92.17245	96.3431	Up_tDCS
**Sec31a**	-0.73467	5.04E-01	0.00E+00	2.944713	2.9398247	2.95943	2.026726	1.007793	2.025768	2.028276	Down_tDCS
**Serac1**	0.067775	8.98E-01	0.00E+00	6.870998	11.759299	8.878289	6.080179	13.10131	11.14172	8.113103	Up_tDCS
**Sestd1**	-0.10054	7.87E-01	0.00E+00	16.68671	16.659007	18.74305	16.21381	16.12469	15.19326	17.24034	Down_tDCS
**Sgca**	-0.02177	9.60E-01	0.00E+00	13.742	14.699123	10.85124	10.13363	13.10131	13.16749	15.21207	Down_tDCS
**Sgk1**	0.224779	3.19E-01	0.00E+00	60.85741	42.137487	46.3644	61.81516	70.54552	55.70862	44.62207	Up_tDCS
**Shh**	-0.37253	6.70E-01	0.00E+00	3.926285	3.9197663	3.945906	3.04009	3.023379	3.038652	3.042414	Down_tDCS
**Slc24a3**	0.081798	6.61E-01	0.00E+00	59.87584	58.796494	59.18859	60.80179	64.49876	61.78593	63.89068	Up_tDCS
**Slc24a5**	0.212507	8.51E-01	0.00E+00	1.963142	1.9598831	1.972953	2.026726	2.015586	3.038652	2.028276	Up_tDCS
**Slc25a13**	0.295214	6.51E-01	0.00E+00	5.889427	6.8595909	3.945906	7.093543	8.062345	4.051536	8.113103	Up_tDCS
**Slc25a24**	-0.37283	7.32E-01	0.00E+00	2.944713	1.9598831	2.95943	2.026726	2.015586	2.025768	2.028276	Down_tDCS
**Slc30a1**	-0.16976	6.52E-01	0.00E+00	16.68671	17.638948	17.75658	15.20045	16.12469	14.18038	16.22621	Down_tDCS
**Slc37a4**	-0.06842	8.00E-01	0.00E+00	33.37342	31.35813	31.56725	30.4009	32.24938	30.38652	29.41	Down_tDCS
**Slc3a2**	0.15508	2.83E-01	0.00E+00	99.13868	101.91392	92.72879	106.4031	108.8417	111.4172	109.5269	Up_tDCS
**Slc8a1**	-0.27352	5.35E-01	0.00E+00	12.76042	10.779357	17.75658	11.147	12.09352	11.14172	11.15552	Down_tDCS
**Slc8a3**	0.30513	8.23E-01	0.00E+00	0	2.9398247	1.972953	1.013363	3.023379	1.012884	3.042414	Up_tDCS
**Slc9a1**	-0.06101	8.36E-01	0.00E+00	27.48399	27.438364	26.63487	27.36081	25.19483	25.3221	26.36758	Down_tDCS
**Slit2**	0.113276	8.98E-01	0.00E+00	2.944713	3.9197663	2.95943	3.04009	3.023379	4.051536	4.056551	Up_tDCS
**Slit3**	-0.12479	7.52E-01	0.00E+00	15.70514	13.719182	17.75658	15.20045	14.1091	14.18038	14.19793	Down_tDCS
**Smad3**	-0.19781	6.94E-01	0.00E+00	8.83414	9.7994156	11.83772	8.106906	10.07793	9.115956	8.113103	Down_tDCS
**Smoc2**	0.211316	9.21E-01	0.00E+00	0	0.9799416	0.986477	1.013363	1.007793	1.012884	0	Up_tDCS
**Snta1**	0.028128	9.05E-01	0.00E+00	43.18913	43.117429	36.49963	42.56126	43.3351	37.47671	43.60793	Up_tDCS
**Snx10**	0.008771	9.57E-01	0.00E+00	76.56255	76.435442	80.89108	79.04233	74.57669	81.03072	79.10275	Up_tDCS
**Spg7**	0.021228	9.36E-01	0.00E+00	32.39185	37.237779	30.58077	35.46771	35.27276	33.42517	31.43827	Up_tDCS
**Sphk1**	-0.37165	7.91E-01	0.00E+00	1.963142	1.9598831	0.986477	2.026726	1.007793	1.012884	1.014138	Down_tDCS
**Spock1**	-0.02959	8.38E-01	0.00E+00	101.1018	100.93398	105.553	102.3497	99.77152	100.2755	99.38551	Down_tDCS
**Sptan1**	-0.07292	8.31E-01	0.00E+00	26.50242	18.61889	18.74305	21.28063	21.16366	19.2448	19.26862	Down_tDCS
**Sptbn1**	0.027578	8.49E-01	0.00E+00	102.0834	96.034273	115.4178	107.4165	107.8339	105.3399	105.4703	Up_tDCS
**Srr**	-0.37214	6.74E-01	0.00E+00	3.926285	3.9197663	3.945906	3.04009	2.015586	3.038652	4.056551	Down_tDCS
**Stc2**	-0.27295	7.30E-01	0.00E+00	4.907856	3.9197663	4.932383	4.053453	3.023379	4.051536	4.056551	Down_tDCS
**Stim1**	-0.02075	9.26E-01	0.00E+00	42.20756	45.077312	44.39144	44.58798	42.32731	42.54113	43.60793	Down_tDCS
**Stim2**	-0.13493	6.86E-01	0.00E+00	21.59456	20.578773	23.67544	20.26726	19.14807	21.27056	19.26862	Down_tDCS
**Stoml2**	0.198124	3.10E-01	0.00E+00	50.06013	63.696202	50.3103	61.81516	61.47538	63.81169	63.89068	Up_tDCS
**Stx17**	-0.23572	7.18E-01	0.00E+00	5.889427	4.8997078	8.878289	5.066816	6.046759	6.077304	5.070689	Down_tDCS
**Stx2**	-0.20746	6.73E-01	0.00E+00	10.79728	11.759299	9.864765	8.106906	11.08572	7.090188	11.15552	Down_tDCS
**Sulf1**	-0.24763	7.86E-01	0.00E+00	2.944713	2.9398247	4.932383	2.026726	3.023379	4.051536	3.042414	Down_tDCS
**Sulf2**	0.033626	8.49E-01	0.00E+00	68.70998	66.636026	64.12097	69.92206	68.52993	66.85035	66.9331	Up_tDCS
**Sumo1**	0.247848	1.14E-01	0.00E+00	79.50726	81.33515	76.94517	92.21606	89.69359	96.22398	98.37137	Up_tDCS
**Syt13**	-0.1218	4.78E-01	0.00E+00	78.52569	72.515676	81.87755	79.04233	69.53772	71.91477	64.90482	Down_tDCS
**Syt2**	-0.23449	3.08E-01	0.00E+00	47.11541	42.137487	57.21564	41.54789	45.35069	36.46383	42.59379	Down_tDCS
**Syt4**	0.005425	9.76E-01	0.00E+00	66.74684	64.676143	72.01279	73.97552	62.48317	70.90188	64.90482	Up_tDCS
**Tac1**	0.112986	7.67E-01	0.00E+00	12.76042	20.578773	15.78362	17.22718	18.14028	20.25768	15.21207	Up_tDCS
**Tacr1**	-0.51001	5.91E-01	0.00E+00	3.926285	2.9398247	3.945906	3.04009	2.015586	2.025768	3.042414	Down_tDCS
**Tbc1d9**	-0.08324	7.60E-01	0.00E+00	31.41028	29.398247	36.49963	31.41426	31.24159	31.3994	28.39586	Down_tDCS
**Tbxa2r**	-0.56483	6.19E-01	0.00E+00	2.944713	2.9398247	1.972953	2.026726	2.015586	1.012884	2.028276	Down_tDCS
**Tgfb1**	0.212673	6.44E-01	0.00E+00	8.83414	12.73924	9.864765	11.147	11.08572	13.16749	13.18379	Up_tDCS
**Tgm2**	0.300419	4.81E-01	0.00E+00	12.76042	11.759299	11.83772	11.147	18.14028	17.21903	13.18379	Up_tDCS
**Tgm3**	0.112472	9.30E-01	0.00E+00	0.981571	1.9598831	1.972953	2.026726	2.015586	2.025768	1.014138	Up_tDCS
**Thada**	-0.03155	9.65E-01	0.00E+00	4.907856	4.8997078	4.932383	4.053453	5.038966	5.06442	5.070689	Down_tDCS
**Thbd**	0.212284	7.86E-01	0.00E+00	3.926285	3.9197663	3.945906	4.053453	5.038966	5.06442	4.056551	Up_tDCS
**Thbs2**	-0.37337	8.09E-01	0.00E+00	0.981571	0.9799416	1.972953	1.013363	1.007793	1.012884	1.014138	Down_tDCS
**Tll1**	-0.37337	8.09E-01	0.00E+00	0.981571	0.9799416	1.972953	1.013363	1.007793	1.012884	1.014138	Down_tDCS
**Tmco1**	0.013402	9.49E-01	0.00E+00	48.09699	49.97702	49.32383	45.60135	47.36628	53.68285	51.72103	Up_tDCS
**Tmem165**	-0.02208	9.41E-01	0.00E+00	26.50242	25.478481	26.63487	24.32072	26.20262	26.33498	26.36758	Down_tDCS
**Tmtc2**	0.042506	9.47E-01	0.00E+00	5.889427	5.8796494	5.918859	6.080179	6.046759	6.077304	6.084827	Up_tDCS
**Tnf**	0.042505	9.79E-01	0.00E+00	0.981571	0.9799416	0.986477	1.013363	1.007793	1.012884	1.014138	Up_tDCS
**Tnnc1**	0.322696	5.68E-01	0.00E+00	5.889427	7.8395325	6.905336	7.093543	8.062345	11.14172	8.113103	Up_tDCS
**Tnnt1**	0.25568	7.55E-01	0.00E+00	2.944713	4.8997078	2.95943	4.053453	5.038966	4.051536	4.056551	Up_tDCS
**Tnnt2**	-0.19795	9.59E-01	0.00E+00	0.981571	0	0	1.013363	0	0	0	Down_tDCS
**Tnrc6a**	0.211228	8.57E-01	0.00E+00	0.981571	1.9598831	2.95943	2.026726	3.023379	3.038652	1.014138	Up_tDCS
**Tnrc6b**	-0.52487	4.56E-01	0.00E+00	6.870998	4.8997078	7.891812	7.093543	4.031172	4.051536	3.042414	Down_tDCS
**Tpcn1**	0.090851	7.23E-01	0.00E+00	34.35499	36.257838	30.58077	36.48108	36.28055	33.42517	37.5231	Up_tDCS
**Tpd52**	0.018343	9.13E-01	0.00E+00	76.56255	75.4555	69.05336	76.00224	73.5689	74.95342	74.03206	Up_tDCS
**Tph2**	-0.15023	9.02E-01	0.00E+00	1.963142	1.9598831	1.972953	2.026726	2.015586	1.012884	2.028276	Down_tDCS
**Tpm4**	0.151042	5.01E-01	0.00E+00	42.20756	43.117429	40.44554	41.54789	48.37407	48.61843	47.66448	Up_tDCS
**Trim24**	-0.09945	7.99E-01	0.00E+00	15.70514	16.659007	14.79715	15.20045	15.1169	14.18038	14.19793	Down_tDCS

**Trim36**	-0.2152	6.99E-01	0.00E+00	9.815711	6.8595909	8.878289	6.080179	8.062345	7.090188	8.113103	Down_tDCS
**Trpc1**	-0.12837	7.06E-01	0.00E+00	19.63142	19.598831	24.66191	19.2539	20.15586	19.2448	19.26862	Down_tDCS
**Trpc3**	-0.11387	7.56E-01	0.00E+00	15.70514	19.598831	19.72953	15.20045	17.13248	18.23191	17.24034	Down_tDCS
**Trpc5**	-0.45114	5.22E-01	0.00E+00	4.907856	4.8997078	8.878289	5.066816	4.031172	5.06442	4.056551	Down_tDCS
**Trpc6**	-0.10442	8.04E-01	0.00E+00	12.76042	12.73924	17.75658	10.13363	14.1091	14.18038	15.21207	Down_tDCS
**Trpm2**	0.042632	9.18E-01	0.00E+00	12.76042	14.699123	13.81067	12.16036	13.10131	14.18038	17.24034	Up_tDCS
**Trpm4**	0.627064	6.35E-01	0.00E+00	1.963142	0.9799416	0.986477	1.013363	3.023379	2.025768	2.028276	Up_tDCS
**Trpm7**	-0.26648	7.50E-01	0.00E+00	3.926285	2.9398247	5.918859	3.04009	4.031172	4.051536	3.042414	Down_tDCS
**Trpv1**	-0.15029	9.02E-01	0.00E+00	1.963142	1.9598831	1.972953	1.013363	2.015586	2.025768	2.028276	Down_tDCS
**Trpv3**	0.042505	9.79E-01	0.00E+00	0.981571	0.9799416	0.986477	1.013363	1.007793	1.012884	1.014138	Up_tDCS
**Trpv4**	0.623554	7.76E-01	0.00E+00	0	0	1.972953	1.013363	1.007793	2.025768	0	Up_tDCS
**Trpv6**	-0.10952	7.75E-01	0.00E+00	15.70514	16.659007	16.7701	16.21381	15.1169	15.19326	14.19793	Down_tDCS
**Tspo**	0.557136	3.07E-01	0.00E+00	6.870998	6.8595909	6.905336	8.106906	9.070138	14.18038	9.127241	Up_tDCS
**Txnip**	-0.04714	8.70E-01	0.00E+00	33.37342	32.338072	23.67544	32.42762	28.21821	28.36075	26.36758	Down_tDCS
**Unc13a**	-0.34701	3.88E-01	0.00E+00	18.64985	17.638948	18.74305	15.20045	15.1169	8.103072	19.26862	Down_tDCS
**Vamp7**	-0.06067	7.82E-01	0.00E+00	48.09699	43.117429	51.29678	45.60135	43.3351	48.61843	44.62207	Down_tDCS
**Vapb**	0.042547	8.02E-01	0.00E+00	72.63626	72.515676	70.03983	74.98888	72.5611	74.95342	73.01792	Up_tDCS
**Vcan**	-0.12096	8.42E-01	0.00E+00	6.870998	6.8595909	6.905336	7.093543	6.046759	6.077304	6.084827	Down_tDCS
**Vdac1**	0.081644	7.91E-01	0.00E+00	24.53928	25.478481	21.70248	25.33408	25.19483	24.30922	26.36758	Up_tDCS
**Vldlr**	-0.08554	8.10E-01	0.00E+00	20.61299	16.659007	20.71601	16.21381	21.16366	17.21903	18.25448	Down_tDCS
**Wfs1**	-0.03004	8.66E-01	0.00E+00	67.72841	70.555793	67.0804	70.93543	68.52993	67.86323	60.84827	Down_tDCS
**Wnt5a**	-0.11014	9.34E-01	0.00E+00	0.981571	1.9598831	1.972953	1.013363	2.015586	2.025768	1.014138	Down_tDCS
**Xk**	-0.46589	5.49E-01	0.00E+00	3.926285	4.8997078	6.905336	5.066816	3.023379	3.038652	4.056551	Down_tDCS
**Zmpste24**	-0.10048	7.87E-01	0.00E+00	17.66828	16.659007	17.75658	15.20045	16.12469	16.20614	17.24034	Down_tDCS

Supplementary Table S8: Expression profiles of 31 'calcium-related' leading-edge genes common to the two calcium-related pathways 'response to external stimuli' and ' immune system process' found to be significant by the GSEA.

Gene name	log2Fold Change	pvalue	padj	Control Sample16	Control Sample15	Control Sample13	250tDCS Sample8	250tDCS Sample7	250tDCS Sample6	250tDCS Sample5	UP/ DOWN_tDCS
**Apoe**	0.48737	2.32E-08	8.89E-07	814.704	848.62939	671.7905	1133.9535	1069.2685	1079.7344	1082.085	UP_tDCS
**Actg1**	0.47182	2.70E-07	8.69E-06	295.4529	303.78188	269.3081	409.39875	383.96918	406.1665	406.6693	UP_tDCS
**App**	0.42943	5.59E-04	3.69E-03	125.6411	121.51275	132.1879	166.19157	169.30924	170.16452	175.4458	UP_tDCS
**A2m**	0.67865	3.21E-01	0.00E+00	3.926285	4.8997078	4.932383	6.0801795	4.0311725	8.1030722	11.15552	UP_tDCS
**Aif1**	0.50832	3.96E-02	0.00E+00	32.39185	39.197663	33.5402	40.53453	43.335104	54.695738	60.84827	UP_tDCS
**Anxa3**	0.49451	3.31E-02	0.00E+00	36.31813	46.057253	34.52668	46.614709	50.389656	56.721506	65.91896	UP_tDCS
**Apobec1**	0.95114	5.95E-01	0.00E+00	0.981571	0.9799416	0	1.0133632	1.0077931	1.012884	2.028276	UP_tDCS
**Ccl19**	0.74379	4.84E-01	0.00E+00	1.963142	2.9398247	0.986477	5.0668162	3.0233793	3.0386521	2.028276	UP_tDCS
**Ccl2**	0.79706	5.65E-01	0.00E+00	0.981571	0.9799416	1.972953	1.0133632	1.0077931	6.0773042	1.014138	UP_tDCS
**Ccl3**	0.62822	7.54E-01	0.00E+00	0	0.9799416	0.986477	1.0133632	0	2.0257681	1.014138	UP_tDCS
**Ccl4**	0.94857	5.96E-01	0.00E+00	0.981571	0	0.986477	1.0133632	1.0077931	2.0257681	1.014138	UP_tDCS
**Ccl7**	1.21154	5.22E-01	0.00E+00	0.981571	0	0.986477	0	1.0077931	4.0515361	1.014138	UP_tDCS
**Ccr5**	0.73738	1.85E-01	0.00E+00	5.889427	6.8595909	5.918859	8.106906	9.070138	10.12884	14.19793	UP_tDCS
**Cd40**	0.43519	7.48E-01	0.00E+00	0.981571	1.9598831	0.986477	1.0133632	2.0155862	2.0257681	2.028276	UP_tDCS
**Cd84**	0.45832	6.27E-01	0.00E+00	0.981571	4.8997078	2.95943	3.0400897	3.0233793	3.0386521	7.098965	UP_tDCS
**Cxcl10**	2.85085	3.51E-02	0.00E+00	0.981571	0.9799416	0.986477	1.0133632	1.0077931	23.296333	3.042414	UP_tDCS
**Cxcl11**	3.29288	1.61E-01	0.00E+00	0	0	0	0	1.0077931	5.0644202	1.014138	UP_tDCS
**Cxcl13**	1.84574	3.62E-01	0.00E+00	0	0	0.986477	1.0133632	1.0077931	1.012884	2.028276	UP_tDCS
**Cxcl9**	1.48658	6.97E-01	0.00E+00	0	0	0	0	0	2.0257681	0	UP_tDCS
**Fos**	0.6354	1.18E-02	0.00E+00	30.42871	29.398247	31.56725	48.641436	37.288345	48.618433	54.76344	UP_tDCS
**Gch1**	0.62705	7.38E-01	0.00E+00	0	0.9799416	0.986477	1.0133632	1.0077931	1.012884	1.014138	UP_tDCS
**Lyn**	0.38665	4.32E-01	0.00E+00	8.83414	8.8194741	7.891812	10.133632	10.077931	12.154608	12.16965	UP_tDCS
**Myo1f**	0.62752	5.52E-01	0.00E+00	1.963142	1.9598831	1.972953	2.0267265	3.0233793	3.0386521	4.056551	UP_tDCS
**Plcg2**	0.43519	7.48E-01	0.00E+00	0.981571	1.9598831	0.986477	1.0133632	2.0155862	2.0257681	2.028276	UP_tDCS
**Rab32**	0.36468	7.41E-01	0.00E+00	1.963142	1.9598831	1.972953	2.0267265	2.0155862	3.0386521	3.042414	UP_tDCS
**S100a8**	0.43485	6.61E-01	0.00E+00	3.926285	1.9598831	1.972953	1.0133632	4.0311725	5.0644202	4.056551	UP_tDCS
**S100a9**	1.00597	2.24E-01	0.00E+00	5.889427	1.9598831	1.972953	2.0267265	8.0623449	9.1159563	7.098965	UP_tDCS
**Sell**	2.06963	4.88E-01	0.00E+00	0	0	0	0	1.0077931	1.012884	1.014138	UP_tDCS
**Trpm4**	0.62706	6.35E-01	0.00E+00	1.963142	0.9799416	0.986477	1.0133632	3.0233793	2.0257681	2.028276	UP_tDCS
**Trpv4**	0.62355	7.76E-01	0.00E+00	0	0	1.972953	1.0133632	1.0077931	2.0257681	0	UP_tDCS

Supplementary Table S9: Expression profiles of 14 'calcium-related' leading-edge genes common to the two calcium-related pathways 'neurotransmitter secretion' and 'synaptic membrane' found to be significant by the GSEA.

Gene name	log2Fold Change	pvalue	padj	Control Sample16	Control Sample15	Control Sample13	250tDCS Sample8	250tDCS Sample7	250tDCS Sample6	250tDCS Sample5	UP/ DOWN_tDCS
Adcy1	-0.32783	1.17E-01	0.00E+00	61.838981	52.916844	71.026311	52.69489	56.43641	43.55401	44.62207	DOWN_tDCS
Adra1a	-0.6368	5.05E-01	0.00E+00	3.9262845	1.9598831	5.9188592	3.04009	3.023379	2.025768	2.028276	DOWN_tDCS
Cacna1a	-0.1705	3.91E-01	0.00E+00	55.949555	57.816552	69.053357	55.73498	59.45979	52.66997	48.67862	DOWN_tDCS
Chrna7	-0.37336	6.26E-01	0.00E+00	3.9262845	3.9197663	7.8918123	4.053453	4.031172	4.051536	4.056551	DOWN_tDCS
Chrnb2	-0.17163	5.16E-01	0.00E+00	34.35499	32.338072	38.472585	32.42762	31.24159	30.38652	30.42414	DOWN_tDCS
Drd2	-0.29467	6.70E-01	0.00E+00	5.8894268	4.8997078	6.9053357	5.066816	4.031172	5.06442	5.070689	DOWN_tDCS
Htr1b	-0.25742	6.63E-01	0.00E+00	7.8525691	5.8796494	9.8647653	8.106906	6.046759	6.077304	6.084827	DOWN_tDCS
Htr2a	-0.47613	3.97E-01	0.00E+00	8.8341402	6.8595909	12.824195	8.106906	6.046759	7.090188	6.084827	DOWN_tDCS
Nlgn1	-0.19227	5.13E-01	0.00E+00	27.483992	26.458422	34.526679	26.34744	27.21041	25.3221	24.33931	DOWN_tDCS
Otof	-0.32967	5.07E-01	0.00E+00	9.8157113	9.7994156	12.824195	8.106906	8.062345	9.115956	9.127241	DOWN_tDCS
P2rx2	-2.09308	3.48E-01	0.00E+00	0.9815711	1.9598831	0.9864765	0	1.007793	0	0	DOWN_tDCS
Slc30a1	-0.16976	6.52E-01	0.00E+00	16.686709	17.638948	17.756578	15.20045	16.12469	14.18038	16.22621	DOWN_tDCS
Stx2	-0.20746	6.73E-01	0.00E+00	10.797282	11.759299	9.8647653	8.106906	11.08572	7.090188	11.15552	DOWN_tDCS
Unc13a	-0.34701	3.88E-01	0.00E+00	18.649852	17.638948	18.743054	15.20045	15.1169	8.103072	19.26862	DOWN_tDCS
